# Quintessential Synergy: Concurrent Transient Administration of Integrated Stress Response Inhibitors and BACE1 and/or BACE2 Activators as the Optimal Therapeutic Strategy for Alzheimer’s Disease

**DOI:** 10.3390/ijms25189913

**Published:** 2024-09-13

**Authors:** Vladimir Volloch, Sophia Rits-Volloch

**Affiliations:** 1 Department of Developmental Biology, Harvard School of Dental Medicine, Boston, MA 02115, USA; 2 Division of Molecular Medicine, Children’s Hospital, Boston, MA 02115, USA; 3 Department of Biological Chemistry and Molecular Pharmacology, Harvard Medical School, Boston, MA 02115, USA

**Keywords:** conventional and unconventional Alzheimer’s disease, neuronal integrated stress response (ISR), intraneuronal Aβ (*i*Aβ), AβPP-independent generation of *i*Aβ, amyloid cascade hypothesis 2.0 (ACH2.0), therapeutic strategies for conventional and unconventional Alzheimer’s disease, suppression of the neuronal ISR, depletion of *i*Aβ via the activation of BACE1 and/or BACE2, initiation of translation from the AUG codon encoding Met671 of the intact or 5′-truncated human AβPP mRNA, RNA-dependent asymmetric amplification of human AβPP mRNA

## Abstract

The present study analyzes two potential therapeutic approaches for Alzheimer’s disease (AD). One is the suppression of the neuronal integrated stress response (ISR). Another is the targeted degradation of intraneuronal amyloid-beta (*i*Aβ) via the activation of BACE1 (Beta-site Aβ-protein-precursor Cleaving Enzyme) and/or BACE2. Both approaches are rational. Both are promising. Both have substantial intrinsic limitations. However, when combined in a carefully orchestrated manner into a composite therapy they display a prototypical synergy and constitute the apparently optimal, potentially most effective therapeutic strategy for AD.

## 1. The Amyloid Cascade Hypothesis Theory of Alzheimer’s Disease Is No Longer Viable and Had to Be Replaced

Recently, a new theory of Alzheimer’s disease (AD), designated the Amyloid Cascade Hypothesis 2.0 (ACH2.0), has been introduced [1,2,3,4,5,6,7,8]. This followed the discreditation of the initial and long-standing Amyloid Cascade Hypothesis (ACH) formulated in 1992 [9]. The ACH postulated that the extracellular “deposition of amyloid β protein, the main component of the plaques, is the causative agent of Alzheimer’s pathology and that the neurofibrillary tangles, cell loss, vascular damage, and dementia follow as the direct result of this deposition” [9]. Since then, it has become apparent that extracellular amyloid beta (Aβ) is neither the causative agent nor the driver of the disease (reviewed in [4]). Indeed, despite some encouraging results in transgenic animal models exogenously overexpressing Aβ protein precursor (AβPP) [10,11,12], the depletion of extracellular Aβ in numerous human clinical trials had no beneficial effect whatsoever in advanced symptomatic AD [13,14]. The recently observed marginal effects of lecanemab and donanemab in slowing down the progression of AD at its early symptomatic stages [15,16,17,18,19] were apparently due to the timing of their administration and stemmed not from the interference with the progression of the pathology in AD-committed neurons (which proceeded uninterrupted), but rather from the transient protection of a small neuronal subpopulation where cellular AD pathology did not yet commence. In fact, any drug depleting extracellular Aβ (e.g., antibodies) or interfering with the proteolysis of AβPP (e.g., BACE1 inhibitors), including those that failed in the preceding clinical trials, is expected to yield similar results if administered similarly early in AD (for a detailed analysis see [3,6]). Moreover, the accumulated statistical data showed that there is no good correlation between the levels of extracellular Aβ and the occurrence of AD. Indeed, in a sizable proportion of the aging general population, levels of extracellular Aβ are similar to or even exceed those in AD patients yet the disease does not occur [20,21,22,23,24,25,26]. Inversely, in some AD patients there is no indication of excessive levels of extracellular Aβ [27].

## 2. Intraneuronal Rather than Extracellular Amyloid-Beta Is Indisputably Central in Alzheimer’s Disease 

Despite the above arguments, however, the centrality and, in many cases, the causative role of Aβ in Alzheimer’s disease is indisputable. The basis for this assertion is the observation that, without a single exception, all known mutations that either cause or protect from AD affect various aspects of Aβ only; these aspects include its structure, generation, and intraneuronal degradation. The first such mutation was discovered in 1991 [28]. It was adjacent to the gamma-cleavage site of AβPP, affected the production of Aβ, and segregated with AD. In the following decades, dozens of additional mutations within Aβ, or within AβPP adjacent to Aβ, or within presenilins (PSEN), that either cause the disease (reviewed in [29]) or protect from it [30,31], were detected. They affect exclusively the production, intraneuronal degradation, or structure of Aβ. The occurrence of these mutations, as well as the complete absence of any Aβ-unrelated AD-causing or AD-protecting mutations, constitute the strongest possible argument for the centrality of Aβ in the disease. The determination that extracellular Aβ is neither causative nor central in AD and the notion of the centrality of Aβ in AD are not incompatible. This is because, in addition to extracellular Aβ, there is another category of Aβ, namely intraneuronal Aβ (*i*Aβ), which was shown in numerous investigations to correlate with AD pathology much better than extracellular Aβ [32,33,34,35,36,37,38,39,40,41,42,43,44]. 

## 3. A New Theory of Alzheimer’s Disease Retains the Centrality of Aβ: Amyloid Cascade Hypothesis 2.0

It follows from the above considerations that any new theory of AD must retain the centrality of Aβ. This is what the Amyloid Cascade Hypothesis 2.0 does [1,2,3,4,5,6,7,8] (and what its designation reflects). In the ACH2.0, AD is driven by Aβ (*i*Aβ) produced independently of AβPP and retained within neuronal cells [1,2,3,4,5,6,7,8]. The logic of this assertion is based on the notion of the centrality of Aβ in AD, and is underpinned by the empirical data obtained in massive human clinical trials [13,14]. In a nutshell (reviewed in [1,4]), the removal of extracellular Aβ has no effect on the progression of the disease; therefore, the disease is propelled by intraneuronal Aβ (*i*Aβ). Likewise, a substantial suppression of the production of Aβ in the AβPP proteolytic pathway confers no benefits whatsoever on AD patients; it follows that, in the disease, Aβ is produced predominantly independently of AβPP and is retained intraneuronally as *i*Aβ [1,2,3,4,5,6,7,8]. The AβPP-independent *i*Aβ generation pathway is the active core of AD [1,2,3,4,5,6,7,8]. The ACH2.0 posits that the AβPP-independent *i*Aβ production pathway is activated by the integrated stress response (ISR) elicited in neuronal cells. How the neuronal ISR is elicited and maintained defines the type of Alzheimer’s disease [8]. In “conventional” AD, the neuronal ISR is elicited and the disease is triggered by *i*Aβ produced in the AβPP proteolytic pathway and accumulated over the critical threshold [1,2,3,4,5,6,7,8]. In “unconventional” AD, the integrated stress response in neuronal cells is elicited by stressors distinct from AβPP-derived *i*Aβ [8]. The two therapeutic strategies for AD, considered in the present study, operate distinctly differently in conventional and unconventional types of AD. Therefore, their application and projected effects in conventional and unconventional AD cases are analyzed separately. The first part of the present study describes briefly the mechanisms involved in conventional AD and considers the proposed therapeutic approaches for this form of the disease. The second part of the present study is concerned with the application of the proposed therapies in unconventional AD.

## 4. Conventional AD: From AβPP Proteolysis to the Elicitation of the Neuronal ISR by AβPP-Derived *i*Aβ

As mentioned above, in conventional Alzheimer’s disease, the neuronal ISR is elicited and AD is triggered by AβPP-derived *i*Aβ accumulated over the critical threshold. It follows that in this type of AD, AβPP-derived *i*Aβ causes the disease [8]. This does not imply, however, that intraneuronal accumulation of Aβ produced in the AβPP proteolytic pathway is pathological or a component of the disease. On the contrary, the intraneuronal accumulation of AβPP-derived *i*Aβ is a perfectly physiological process. It occurs in healthy individuals who will not develop AD within their lifetime as well as in future AD patients. As discussed (and illustrated) below, the only difference between these two groups is quantitative: the rate of AβPP-derived *i*Aβ accumulation and the extent of the critical neuronal ISR-triggering threshold. 

### 4.1. Intraneuronal Accumulation of AβPP-Derived iAβ Occurs Physiologically via Two Distinct Pathways

Proteolytically, Aβ is produced by two cleavages within its precursor, AβPP. The first cleavage, by beta-secretase, also known as BACE (Beta-site AβPP Cleaving Enzyme), occurs between residues 671 and 672 and forms the N-end of Aβ. This generates the C-terminal fragment of AβPP comprised of 99 amino acid residues and therefore designated C99. The second cleavage is carried out by gamma-secretase; it occurs within a small internal segment of C99 and results in Aβ molecules of various length, most often 40 or 42 amino acid residues. All proteolytic cleavages of AβPP take place on cellular membranes. The gamma-cleavage typically occurs on the plasma membrane and the resulting Aβ is secreted. A small proportion of gamma-cleavages take place on intraneuronal membranes within various organelles [45,46,47,48,49,50,51,52,53]; Aβ resulting from such cleavages is not secreted but is retained within neuronal cells as *i*Aβ. 

The retention of AβPP-derived Aβ formed on the intraneuronal membranes is one of two ways to generate *i*Aβ in the AβPP proteolytic pathway (reviewed in [1,4]). The other way is the internalization of secreted Aβ via its cellular uptake, which constitutes, in fact, a conversion of a fraction of secreted extracellular Aβ into *i*Aβ [54,55,56,57,58,59]. This process appears to require the oligomerization of Aβ prior to its uptake [58,59]. Therefore, the fact that secreted Aβ42 is internalized at the rate twice that of Aβ40 [59] is apparently due to the propensity of the former to form aggregates. Thus, the observed increased cellular toxicity of Aβ42 can be explained not only by its aggregation within the cell but also by a significantly increased rate of its cellular uptake [59], a process which involves a number of defined cellular receptors [60,61,62,63,64,65,66,67,68].

### 4.2. AβPP-Derived iAβ, Accumulated over the Critical Threshold, Elicits the Neuronal Integrated Stress Response via Activation of the eIF2α Kinases PKR and/or HRI 

The AβPP-derived *i*Aβ-triggered elicitation of the neuronal integrated stress response occurs via site-specific phosphorylation of the eukaryotic translation initiation factor 2 alpha (eIF2α) at the serine residue in position 51 (Ser51). Phosphorylation of eIF2α at Ser51 can be carried out by any of the members of the family of eIF2α kinases. This family includes the following four kinases: PKR (protein kinase double-stranded RNA-dependent), PERK (PKR-like ER kinase), HRI (heme-regulated inhibitor), and GCN2 (general control non-derepressible-2). When accumulated to sufficient levels, AβPP-derived *i*Aβ is capable of activating, via distinct pathways, two of these kinases, namely PKR [69,70,71,72,73,74,75] and HRI. Aβ-mediated activation of the PKR kinase was indeed demonstrated in cells overexpressing the former [69,70,71]. Even more importantly, the occurrence of the activated PKR was detected in neuronal cells of AD patients [72,73]. *i*Aβ-mediated activation of PKR may happen in multiple ways. One, shown in mouse models, involves TNFα [74]. Another, apparently mediated by the “PKR activator” (PACT), is indicated by the observed co-localization of PACT and active PKR within deteriorating neuronal cells of AD patients [75]. 

*i*Aβ-mediated activation of HRI, on the other hand, occurs as a consequence of mitochondrial distress. *i*Aβ-caused mitochondrial dysfunction is an early symptomatic manifestation of AD and, as such, has been studied in great detail [76,77,78,79,80,81,82,83,84,85,86,87,88,89,90,91,92,93]. Among the most important findings resulting from these studies is the determination that mitochondrial dysfunction activates the HRI kinase in a variety of cell types including neuronal cells [94,95]. This occurs in two stages. First, mitochondrial distress activates mitochondrial protease OMA1 [94,95]. In turn, activated OMA1 cleaves, in a site-specific manner, another mitochondrial protein, DELE1 [94,95]. Subsequently, one of the DELE1 fragments generated by OMA1 cleavage is transferred to the cytosol. This fragment has an affinity to HRI; following their binding, the kinase is activated [94,95]. When either PKR or HRI or both are activated in neuronal cells (following the accumulation of AβPP-derived iAβ to the critical threshold), eIF2α is phosphorylated at Ser51 and the elicitation of the neuronal integrated stress response ensues.

## 5. In Conventional AD, the Neuronal ISR Is the Bridge between *i*Aβ Derived by AβPP Proteolysis and *i*Aβ Generated in the AβPP-Independent Pathway

The cellular integrated stress response is an evolutionary preserved signaling pathway that is activated by a great variety of both pathological and environmental stressors stemming from inflammation, protein aggregation and/or mis-folding, bacterial and viral infections, deficiencies in protein homeostasis, and insufficient nutrition [96,97,98,99,100,101,102,103,104,105]. This type of cellular stress response is designated as “integrated” because the effects of all various stressors capable of eliciting it converge on a single occurrence, namely the phosphorylation of eIF2α at Ser51. As mentioned above, this event is mediated by four eIF2α kinases: PKR, PERK, GCN2, and HRI. The consequences of this event are rather dramatic. Both cellular transcription and translation are profoundly re-programmed. Transcriptionally, an array of new transcription factors is activated and, consequently, the gene expression pattern is amended. Translationally, the total cellular protein production is drastically suppressed. Simultaneously with the suppression of total cellular protein synthesis, however, the production of proteins from a small subset of mRNA species is initiated. 

The ACH2.0 posits that among the small subset of proteins whose production in the neuronal cells is enabled under the integrated stress response conditions are crucial components that are required for the operation of the AβPP-independent *i*Aβ production pathway and that are not present in the neurons under the regular (i.e., non-ISR) circumstances [4]. Cellular mechanisms capable of supporting the AβPP-independent *i*Aβ generation pathway are elaborated upon elsewhere [1,2,3,4,5,6,7,8] and also briefly discussed below (Section 19, Section 20, Section 21 and Section 22), but with any potential mechanism the entire output of the AβPP-independent production of Aβ is retained within the neurons as *i*Aβ. Consequently, following the elicitation of the neuronal ISR and the activation of the AβPP-independent production of *i*Aβ, the latter rapidly accumulates and reaches levels (unattainable by AβPP proteolysis alone) causing AD pathology and driving the disease.

It should be mentioned at this point that the operational AβPP-independent *i*Aβ generation pathway and, therefore, the occurrence of AD appear to be limited to humans or, at least, to be species-specific (reviewed in [7]). This is because, in addition to the availability of some essential components provided under the ISR conditions, the operation of the AβPP-independent *i*Aβ production pathway has certain specific requirements (discussed in Section 20 and Section 21) that are met in humans but not in other species such as mice. This is the reason why, despite acute exogenous overproduction of Aβ and substantial accumulation of AβPP-derived *i*Aβ, transgenic mouse models of AD do not develop the disease [7] (further discussed in Section 22 below). In these models, AβPP-derived *i*Aβ accumulates to the levels above the T1 threshold and triggers the elicitation of the neuronal integrated stress response. However, because the AβPP-independent *i*Aβ production pathway remains inoperative, *i*Aβ does not reach AD pathology-causing levels. Cognitive impairments observed in these models are due to deficiencies in neuronal plasticity, memory formation, and learning. All these processes require new protein synthesis, which is largely suppressed by the ISR. Thus, in transgenic mouse AD models, cognitive impairments are triggered not by AD but rather by the neuronal integrated stress response elicited by AβPP-derived *i*Aβ [106,107,108,109,110,111,112,113,114,115,116,117,118,119,120,121]. This conclusion is supported by the observations that when the ISR is prevented in these models, no cognitive impairments occur, and when the ISR is inhibited, cognitive impairments are abrogated [122,123,124,125,126,127,128,129,130]. Note that the present study addresses only the neuronal ISR; whenever the term “ISR” is used, it signifies the neuronal ISR.

## 6. The Dynamics of AβPP-Derived *i*Aβ Accumulation Leading to the Crossing of the Critical ISR-Triggering Threshold Determines the Occurrence and Timing of Conventional AD or the Absence Thereof

In view of the above considerations, the initial trigger of conventional Alzheimer’s disease is the crossing of the “critical threshold” by AβPP-derived *i*Aβ in the affected neurons. Such an occurrence initiates a cascade of molecular events, including activation of the defined eIF2α kinases, site-specific phosphorylation of eIF2α at the Ser51, elicitation of the neuronal integrated stress response, ignition of the AβPP-independent *i*Aβ generation pathway, phosphorylation of the tau protein at multiple positions, formation of neurofibrillary tangles (NFTs) [131,132,133,134], and, eventually, neuronal death by apoptosis or necroptosis [135]. The present section addresses various aspects defining the timing of the crossing of the critical ISR-eliciting threshold by AβPP-derived *i*Aβ (or the absence thereof) and, consequently, of the commencement of AD. The aspects of the operation of the neuronal ISR-activated AβPP-independent *i*Aβ production pathway, which underlies the rapid accumulation of *i*Aβ in AD-affected neurons and drives the disease, are discussed in Section 9 below.

### 6.1. Dynamics of AβPP-Derived iAβ Accumulation in Health and AD: Rate of Accumulation as a Decisive Variable

**Figure 1:**
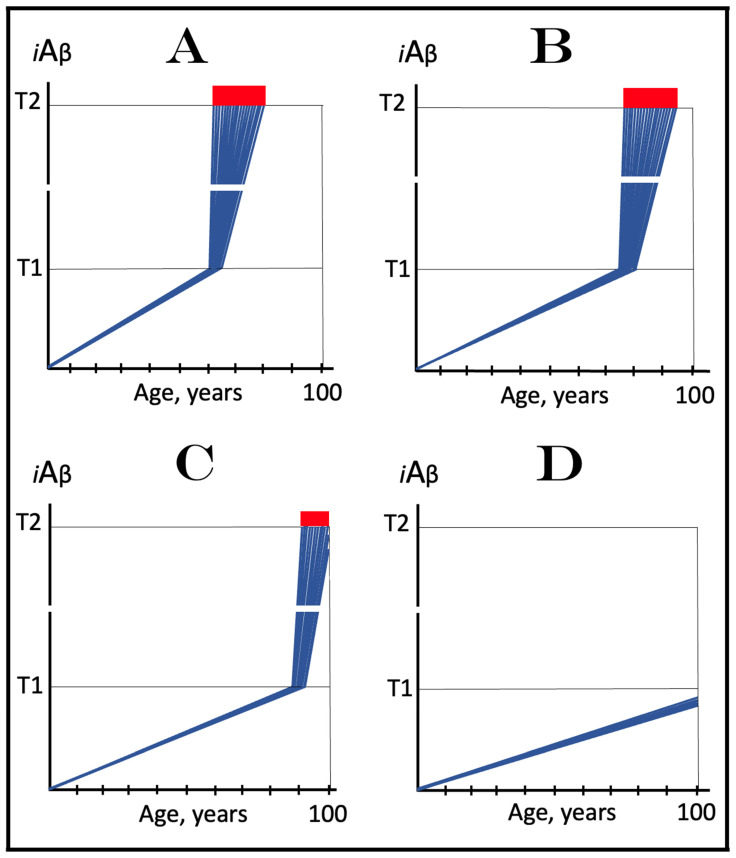
**Dynamics of AβPP-derived iAβ accumulation in health and AD: rate of accumulation as a decisive variable**. *i*Aβ: intraneuronal Aβ. ***T*1** *threshold*: levels of *i*Aβ that trigger the activation of the eIF2α kinases PKR and/or HRI, elicitation of the neuronal ISR, and initiation of the AβPP-independent production of *i*Aβ. ***T*2** *threshold*: levels of *i*Aβ, produced mainly in the AβPP-independent pathway, which trigger neuronal death. *Blue lines*: levels of *i*Aβ in individual neurons of a person. *Red Boxes*: apoptotic zone; the fraction of neurons that have committed apoptosis (or necroptosis) or are dead. The rate of accumulation of AβPP-derived *i*Aβ is the only variable in this figure; all other parameters are presumed constant. The occurrence of AD depends on a sufficient rate of accumulation of AβPP-derived *i*Aβ and its timing is inversely proportional to the latter. *Panel* (**A**): the rate of accumulation of AβPP-derived *i*Aβ is relatively high; the T1 threshold is crossed and, consequently, AD commences at about 65 years of age. *Panels* (**B**,**C**): the rate of accumulation of AβPP-derived *i*Aβ decreases and the timing of the T1 crossing (and of the commencement of AD) increases; in panel (**B**), the crossing occurs and the disease commences at about 80 and in panel (**C**) at about 90 years of age. *Panel* (**D**): the rate of accumulation of AβPP-derived *i*Aβ is such that neither is the T1 threshold crossed nor does AD occur within the lifetime of the individual. Note that with a sufficiently extended lifespan, both the T1 crossing and the occurrence of conventional AD would be inevitable at any rate of the accumulation of AβPP-derived *i*Aβ.

The role of the rate of accumulation of AβPP-derived *i*Aβ in the timing of reaching and crossing the critical ISR-eliciting threshold (designated T1) is considered in Figure 1. In this figure, the extent of the T1 threshold is assumed to be relatively low so that no cellular damages mediated by *i*Aβ occur prior to the T1 crossing (this choice reflects the fact, discussed and referenced below, that the low T1 threshold indeed predisposes to AD), and the only variable is the rate of AβPP-derived *i*Aβ accumulation. As shown in the figure, the timing of the crossing of the T1 threshold by AβPP-derived *i*Aβ is clearly a function of the rate of its accumulation, with the former inversely proportional to the latter. In panel A of Figure 1, this variable is such that the crossing occurs, and, consequently, the disease commences at about 65 years of age. As the rate of the accumulation of AβPP-derived *i*Aβ decreases, the timing of the T1 crossing and of the commencement of AD increases. In panel B, the rate of AβPP-derived *i*Aβ accumulation is such that the disease commences at about 80 years of age. In panel C, the rate is even lower; accordingly, the T1 crossing occurs and the disease commences at about 90 years of age. Inevitably, when the rate of accumulation of AβPP-derived *i*Aβ is sufficiently low, the T1 threshold is not reached within the lifetime of an individual and, accordingly, no AD occurs. This scenario is illustrated in panel D of Figure 1. This, in fact, is all that stands between health and conventional Alzheimer’s disease, namely the crossing of the T1 threshold by AβPP-derived *i*Aβ or the lack thereof. 

### 6.2. The Conditionality of Stage One of Alzheimer’s Disease

Within the framework of the ACH2.0, it is evident that the dynamics of accumulation of *i*Aβ is single-phased in health but double-phased in conventional AD. Phases of accumulation of *i*Aβ correspond to stages of AD. Thus, AD is a two-stage disease. In Stage One, *i*Aβ is derived solely by the proteolysis of AβPP. It accumulates via two distinct mechanisms, namely the cellular uptake of the secreted extracellular Aβ and the retention of Aβ (*i*Aβ) resulting from the AβPP processing on the intraneuronal membranes. The crossing of the T1 threshold by AβPP-derived *i*Aβ triggers Stage Two of AD. The AβPP-independent production of *i*Aβ is activated. The rapidly increasing levels of *i*Aβ drive AD pathology and eventually cause neuronal loss. Only at this stage of the disease do AD symptoms manifest. However, designating the sub-T1 accumulation of AβPP-derived *i*Aβ as the first stage of AD leads to an apparent paradox. This is because this accumulation occurs physiologically and, moreover, there is no disease at this stage. A clear example is presented in panel D of Figure 1. AβPP-derived *i*Aβ accumulates throughout the lifetime but it is obviously not indicative of Stage One of AD because it does not reach the T1 threshold and there is neither Stage Two of AD nor, evidently, the disease. It follows that Stage One of AD is actual only if the disease eventually occurs; in other words, “Stage One of AD” becomes such only retroactively. It is conditional on AβPP-derived *i*Aβ crossing the T1 threshold and triggering the AβPP-independent *i*Aβ production pathway. In the absence of the latter, the accumulation of the AβPP-derived iAβ is just a normal physiological process.

### 6.3. The Occurrence of Conventional AD Is a Function of Longevity: Inevitability of the Disease within the Unrestricted Lifespan

It follows from the above deliberations that conventional Alzheimer’s disease is inevitable provided the lifespan is sufficiently extended. Indeed, if levels of AβPP-derived *i*Aβ are steadily increasing (at any rate), linearly or otherwise, the crossing of the T1 threshold and the consequent commencement of AD is only a function of time and, as such, are inevitable given sufficient lifespan. Consider, for example, the situation depicted in panel D of Figure 1. In this panel, levels of AβPP-derived *i*Aβ are steadily increasing. They do not reach the T1 threshold by the time the lifespan expires as shown. This is the only reason why conventional AD does not occur in this scenario. If the lifespan shown in panel D of Figure 1 were to continue, the T1 threshold would be crossed and the disease would commence at about 115 years of age. Currently, the majority of the general population does not develop AD. This is simply because they run out of time, i.e., their lifetime. Only about 5% of 65-year-olds develop the disease. This proportion increases to about 15% at the age of 75 and to about 35% in 85-year-olds. At the time of Dr. Alois Alzheimer, the disease, which he first described in 1906, was a rarity because the life expectancy at that time was much shorter than currently (under 50 years in Germany; as an example, Dr. Alzheimer himself died at the age of 51 years in 1915). It can be predicted, therefore, with a great degree of certainty that the proportion of the population that develops AD will steadily increase with the increase in longevity until the disease becomes an eventuality rather than a possibility, and the only way to counteract this is to develop effective preventive and curative therapies.

## 7. Validation of the Notion That the Occurrence and Timing of Conventional AD Is a Function of the Rate of Accumulation of AβPP-Derived *i*Aβ: All Known Mutations Either Causing AD or Protecting from AD as Well as Factors Predisposing to AD Act via Alterations of the Rate of Accumulation of AβPP-Derived *i*Aβ

The notion introduced in the preceding section, namely that the occurrence and timing of AD is a function of the rate of accumulation of AβPP-derived *i*Aβ is, in fact, verifiable. The clear prediction of the considerations discussed above is that the increase of this rate should cause AD or, at least, accelerate its occurrence, and that the decrease of this rate should protect from the disease or delay its commencement. The best way to test this prediction is to analyze the effects of the mutations that either cause AD or protect from it and of the factors that predispose to the disease. The results of such analysis are overwhelming: ALL known mutations that cause AD, without an exception, as well as the factors predisposing to the disease increase the rate of AβPP-derived *i*Aβ accumulation, whereas the only mutation known to protect from AD (as well as from aging-associated cognitive decline, AACD) decreases it (reviewed in [4]). A good example is ApoE4, a major feature predisposing to AD and a recognized major risk factor for the occurrence of the disease. All ApoE isoforms are capable of facilitating the importation of extracellular Aβ, i.e., its conversion into *i*Aβ, but ApoE4 does it with much greater efficiency than other ApoE variants [37]. Thus, the occurrence of ApoE4 (rather than of other ApoE variants) causes the increased influx of AβPP-derived *i*Aβ, the greater rate of its accumulation, and the hastened crossing of the T1 threshold and commencement of AD, hence its role as a risk factor for the disease [37]. 

In another example, several PSEN mutations that cause the early onset of AD (familial AD, FAD) significantly increase the rate of generation and, subsequently, secretion of the Aβ42 variant [56]. On the other hand, Aβ42 is taken up by the cell with the efficiency twice that of the other major Aβ variant, Aβ40 [55]. These mutations, therefore, significantly increase the influx and thus the rate of accumulation of AβPP-derived *i*Aβ, hence their causative role in FAD [56]. Another class of FAD-causing mutations significantly increases the proportion of gamma-cleavages that occur on intraneuronal membranes within various cellular organelles. Those include certain PSEN mutations [136] as well as the Swedish Aβ mutation [137]. Aβ produced by these gamma-cleavages is retained intraneuronally as *i*Aβ. Consequently, the influx of AβPP-derived *i*Aβ and the rate of its accumulation are increased causing the early crossing of the T1 threshold and the early onset of AD, hence the carriers of these mutations develop FAD [136,137]. 

Another illustration involves an Aβ mutation that suppresses the rate of physiologically occurring cleavages within *i*Aβ (and within Aβ segment of AβPP and C99). As further discussed below, the physiological function of BACE2 appears to be the control of levels of *i*Aβ. BACE2 accomplishes this function by cleavages at positions 19 and 20 of Aβ (both phenylalanines). The Flemish FAD-causing Aβ mutation suppresses the rate of these cleavages [138]. This reduces the efflux of AβPP-derived *i*Aβ (and also increases its influx by suppressing cleavages within Aβ segment of AβPP and C99) and, accordingly, increases the rate of its accumulation, thus causing the early onset of AD. On the other hand, the mechanism of protection from AD (and from AACD) that is conferred to its carriers by the Icelandic Aβ mutation is diametrically opposite. The major activity of BACE1 is the cleavage between residues 671 and 672 of AβPP (designated the β-site); this cleavage generates the N-terminus of Aβ. The minor activity of BACE1 is the cleavage at the residue 10 of Aβ (designated the β’-site; this activity, apparently, also controls physiologically the levels of *i*Aβ). The Icelandic mutation increases the efficiency of BACE1 cleavage at the position Aβ10 [30,31]. This increase is not large: the efficiency of cleavage at the Aβ10 site is elevated only by about 30% in the carriers of the Icelandic Aβ mutation [30]. It is enough, nevertheless, to sufficiently suppress the rate of accumulation of AβPP-derived *i*Aβ and to protect from both AD and AACD (reviewed in [4]). Taken cumulatively, these observations validate the notion that the occurrence (and timing) of conventional AD is a function of the rate of accumulation of AβPP-derived *i*Aβ.

## 8. Effect of the Extent of the T1 Threshold on the Occurrence and Timing of Conventional AD and of AACD 

**Figure 2:**
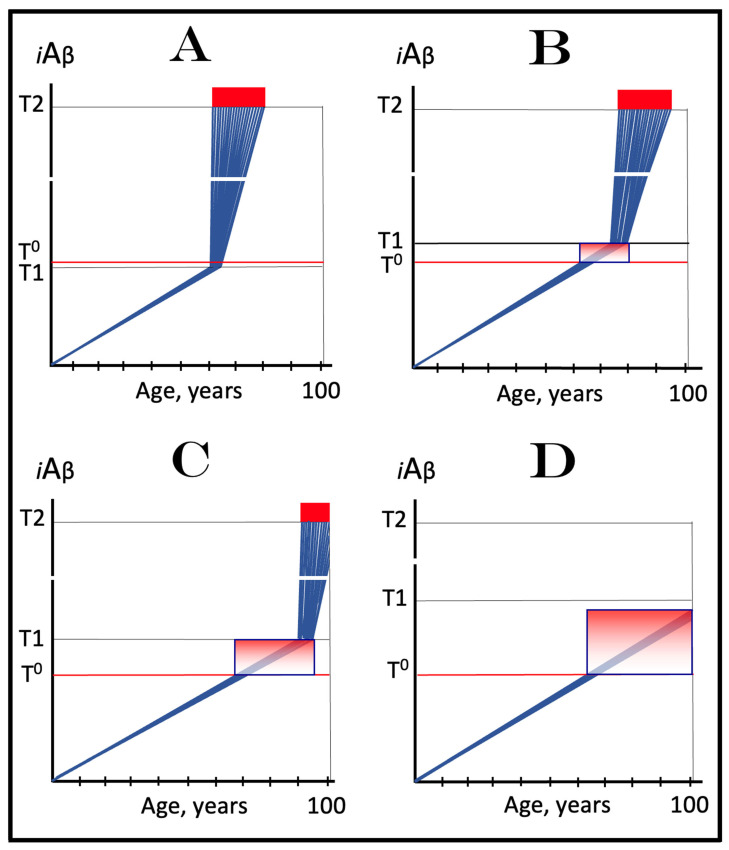
**The timing of AD and occurrence of AACD are functions of the extent of the T1 threshold**. *i*Aβ: intraneuronal Aβ. ***T*^0^** *threshold*: levels of AβPP-derived *i*Aβ that cause neuronal damage manifesting as AACD. ***T*1** *threshold*: levels of *i*Aβ that trigger the activation of the eIF2α kinases PKR and/or HRI, elicitation of the neuronal ISR, and initiation of the AβPP-independent production of *i*Aβ. ***T*2** *threshold*: levels of *i*Aβ, produced mainly in the AβPP-independent pathway, which trigger neuronal death. *Blue lines*: levels of *i*Aβ in individual neurons of a person. *Red Boxes*: apoptotic zone; the fraction of neurons that have committed apoptosis (or necroptosis) or are dead. *Pink* Boxes: zone of the occurrence of AACD; the condition commences with the crossing of the T^0^ threshold (provided it is lower that the T1 threshold) and morphs into AD following the T1 crossing. In this figure, the extent of the T1 threshold is the only variable; all other parameters (including the extent of the T^0^ threshold) are presumed constant. The occurrence of AD is a function of the extent of the T1 threshold and its timing is directly proportional to the latter. *Panel* (**A**): The extent of the T1 threshold is relatively low and below that of the T^0^ threshold. AD commences at about 65 years of age and no AACD occurs. *Panel* (**B**): The extent of the T1 threshold increases and is above that of the T^0^. AACD commences with the T^0^ crossing and morphs into AD when the T1 threshold is crossed. *Panel* (**C**): The extent of the T1 threshold increases further. AD commences later in life and the duration of AACD increases. *Panel* (**D**): The extent of the T1 threshold is such that neither is it crossed nor does AD occur within the lifespan of the individual. AACD, on the other hand, commences with the crossing of the T^0^ threshold and persists through the remaining lifetime.

The effect of a variable extent of the ISR-eliciting T1 threshold in the context of a given rate of the accumulation of AβPP-derived *i*Aβ is analyzed in Figure 2. In panel A of Figure 2, the T1 threshold is sufficiently low and no cellular damage occurs prior to its crossing by AβPP-derived *i*Aβ. With the given rate of accumulation of the latter, the T1 crossing and consequent commencement of AD occur at about 60 years of age. In panels B through D, the extent of the T1 threshold increases with the rate of accumulation of AβPP-derived *i*Aβ remaining constant. Accordingly, the timing of the crossing of the T1 threshold and of the commencement of AD also increases. In panel B of Figure 2, it occurs at about 75 years of age, and in panel C, with an even higher extent of the T1, at about 90 years of age. In panel D of Figure 2, the extent of the T1 threshold is such that at the given rate of its accumulation AβPP-derived *i*Aβ does not reach it within the lifetime of an individual. No T1 crossing takes place, no AβPP-independent *i*Aβ production pathway is activated, and no AD occurs in this scenario. In this context, therefore, the timing of the crossing of the T1 threshold by AβPP-derived *i*Aβ is clearly a function of the extent of the T1, with the former being directly proportional to the latter.

A high extent of the T1 threshold is clearly beneficial because it either delays or prevents AβPP-derived *i*Aβ from reaching and crossing it, and thus delays or prevents the occurrence of AD. However, it extracts a certain price. This price is aging-associated cognitive decline, AACD (reviewed in [4]). In terms of the ACH2.0, AACD is a condition that manifests as a result of the neuronal damage caused by concentrations of AβPP-derived *i*Aβ in the range between the thresholds T^0^ and T1 (pink boxes in Figure 2). When levels of AβPP-derived *i*Aβ cross the T1 threshold, AACD morphs into AD (reviewed in [4]). Thus, by definition, AACD occurs only when the extent of the T^0^ threshold is below that of the T1. In panel A of Figure 2, the extent of the T1 threshold is below that of the T^0^ threshold; accordingly, there is no AACD stage. In panel B of Figure 2, the relationship between the T^0^ and T1 is inversed. AACD commences with the crossing of the T^0^ threshold and morphs into AD with the crossing of the T1. In panel C of Figure 2, the distance between the T^0^ and T1 further increases and so does the duration of AACD prior to its morphing into AD. Finally, in panel D of Figure 2, the extent of the T1 is so high that it is not reached by AβPP-derived *i*Aβ within the lifespan of an individual. In this scenario AACD commences with the crossing of the T^0^ threshold and continues for the remaining lifetime. 

Statistically, the age of the onset of AACD is greater than the age of the onset of sporadic AD [139,140]. Since AACD occurs in a much greater proportion of the general population than AD, this implies that the T^0^ and, consequently, T1 thresholds in the majority of the population are higher than the T1 threshold in a fraction of the population that develops sporadic AD. This, in turn, indicates that the segment of the population that develops sporadic conventional AD is characterized by a low extent of the T1 threshold, which predisposes it to the disease. It follows, therefore, that the human subpopulation that develops sporadic Alzheimer’s disease is not random but rather is enhanced in individuals with the relatively low extent of the T1 threshold and/or the relatively high rate of accumulation of AβPP-derived *i*Aβ. It should be mentioned that the extent of the T1 threshold, i.e., levels of *i*Aβ requisite for the activation of PKR, is probably distinct from that triggering the activation of HRI; this, however, is inconsequential since either would phosphorylate eIF2α and elicit the neuronal ISR. Moreover, the extents of the T1 threshold are probably different in different types of neurons; this could contribute to or underlie the differential vulnerability of distinct types of neuronal cells in Alzheimer’s disease.

Since the aspects of AACD and therapeutic approaches for this condition are addressed extensively elsewhere [4,6], in the present study the discussion of this condition is restricted to the current section; when the term “disease” is used henceforth, it signifies AD rather than AACD. With regard to therapies for AACD, it suffices to mention that therapeutic strategies described below apply to AACD as well as to AD. 

## 9. *i*Aβ Generated in the AβPP-Independent Pathway Drives AD Pathology, Propagates the ISR, and Perpetuates Its Own Production: The AD Engine

The preceding Section 6, Section 7 and Section 8 discussed various aspects related to the physiologically occurring accumulation of AβPP-derived *i*Aβ. The present section addresses the basic principles regulating the operation of the AβPP-independent *i*Aβ production pathway, the active core driving Alzheimer’s disease. As discussed above, in conventional AD the sufficient accumulation of AβPP-derived *i*Aβ triggers the activation of the eIF2α kinases PKR and/or HRI in neuronal cells, and the phosphorylation of eIF2α at its Ser51 follows. This elicits the neuronal integrated stress response. Under the ISR conditions, both transcriptional and translational landscapes in affected neuronal cells are drastically transformed [96,97,98,99,100,101,102,103,104,105]. Cellular gene expression is reprogrammed with the activation of an array of new transcription factors. Total cellular protein synthesis is radically suppressed. Concurrently, translation of a small subset of mRNA species is activated. The cumulative effect is the appearance of a set of cellular proteins not present under regular (i.e., non-ISR) conditions. Presumably, it contains the crucial component(s) required for the operation of the AβPP-independent *i*Aβ generation pathway. With these components available, the AβPP-independent *i*Aβ production pathway is rendered operational. The entire Aβ output of this pathway (the potential mechanisms underlying its operation are discussed in Section 19, Section 20, Section 21 and Section 22 below) is retained within the neurons as *i*Aβ. Levels of *i*Aβ rapidly increase and it reaches cellular concentrations capable of driving the AD pathology and causing the formation of neurofibrillary tangles [131,132,133,134] and, eventually, neuronal death. Importantly, these cellular concentrations of *i*Aβ are unattainable if only the AβPP proteolytic pathway is operational [1,2,3,4,5,6,7,8].

**Figure 3:**
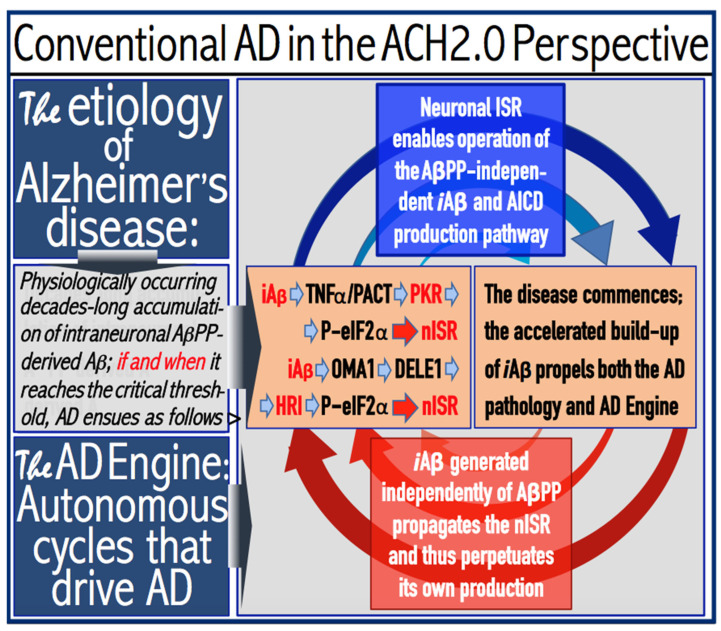
**Conventional Alzheimer’s disease: the ACH2.0 perspective.** *i*Aβ: intraneuronal Aβ. *eIF2α:* eukaryotic translation initiation factor 2 alpha. *PKR and HRI:* eIF2α–specific kinases. *TNFα*: tumor necrosis factor alpha; presumably, it activates the PKR kinase. *PACT*: activator of PKR. *OMA1:* mitochondrial dysfunction-activated mitochondrial protease. *DELE1:* mitochondrial substrate of OMA1; its cleavage results in the activation of HRI. *nISR:* neuronal integrated stress response. It is caused by phosphorylation of eIF2α and manifests in radical transcriptional and translational reprogramming; it provides “missing” components of the AβPP-independent *i*Aβ generation pathway, thus activating it. *AICD:* AβPP Intracellular domain; one of the products of the processing of C99. AβPP-derived *i*Aβ accumulates physiologically in a decades-long process. Conventional AD occurs only if and when AβPP-derived *i*Aβ crosses the critical ISR-eliciting threshold. At this point, PKR and/or HRI are activated, eIF2α is phosphorylated, and the ISR is elicited. Under the ISR conditions, the crucial components of the AβPP-independent *i*Aβ generation pathway are produced and the pathway is initiated. The entire Aβ output of this pathway is retained intraneuronally as *i*Aβ. It rapidly accumulates and reaches AD pathology-causing levels. It is, therefore, the driver of the disease. It also sustains the activity of PKR and HRI, maintains eIF2α in a phosphorylated state, propagates the ISR conditions, and thus perpetuates its own production in the AβPP-independent pathway, thus propelling the progression of AD. The repeated cycles of these activities, symbolized in the figure by the arched red and blue arrows, comprise the engine that drives AD, the “AD Engine”. Note that the increase in *i*Aβ produced independently of AβPP is accompanied by a proportional increase in levels of AICD. The AβPP intracellular domain was shown to be competent of interfering with AD-associated processes but its potential contribution to the disease remains to be determined [141,142,143,144,145,146,147,148,149,150,151,152,153,154,155,156].

Thus, the augmented production of *i*Aβ via its AβPP-independent generation enables the progression of AD. But this is only one of its two major functions. Its second major function is the perpetuation of the operation of the AβPP-independent *i*Aβ production pathway. Indeed, the operation of this pathway depends on the occurrence of the neuronal integrated stress response. If the ISR were to cease, so will the supply of the components necessary for the operation of the AβPP-independent *i*Aβ generation pathway, and its activity will stop. In conventional AD the elicitation of the ISR is mediated by *i*Aβ at the levels exceeding the T1 threshold. Accelerated production of *i*Aβ in the AβPP-independent manner ascertains that as long as the AβPP-independent *i*Aβ production pathway is operational, levels of *i*Aβ are substantially above the T1 threshold. Consequently, the activity of the eIF2α kinases is sustained, phosphorylation of eIF2α at its Ser51 persists, the ISR conditions are maintained, and the pathway generating *i*Aβ independently of AβPP remains operational. Thus, *i*Aβ produced independently of AβPP propagates the neuronal integrated stress response and perpetuates its own production in the AβPP-independent *i*Aβ generation pathway. As illustrated in Figure 3, this feedback cycle constitutes an engine that propels AD, the AD Engine. 

## 10. A Therapeutic Strategy for AD: An Intuitive Approach Targeting the Neuronal ISR 

The preceding discourse makes it apparent that the neuronal integrated stress response is the pivotal constituent of Alzheimer’s disease. In its absence, no crucial components are supplied, no AβPP-independent *i*Aβ production pathway is operational, and no disease can occur [1,2,3,4,5,6,7,8]. This suggests an intuitive therapeutic approach for AD: prevent the neuronal integrated stress response and you will prevent AD, suppress the neuronal ISR in Alzheimer’s patients and you will stop the disease. However, the analysis of this approach, presented below, indicates that this is not necessarily the case.

### 10.1. Inhibition of the Neuronal ISR in the Prevention of Conventional AD 

**Figure 4:**
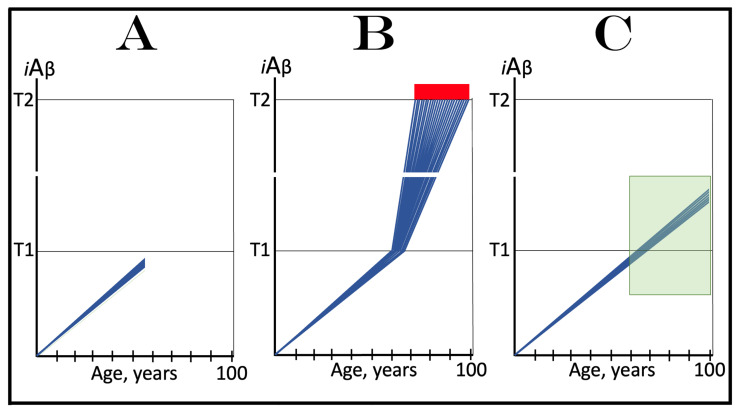
**Effect of suppression of the neuronal ISR in the prevention of conventional AD***. i*Aβ: intraneuronal Aβ. ***T*1** *threshold*: levels of iAβ that trigger the activation of the eIF2α kinases PKR and/or HRI, elicitation of the neuronal ISR, and initiation of the AβPP-independent production of *i*Aβ. ***T*2** *threshold*: levels of *i*Aβ, produced mainly in the AβPP-independent pathway, which trigger neuronal death. *Blue lines*: levels of *i*Aβ in individual neurons of a person. *Red Box*: apoptotic zone; the fraction of neurons that have committed apoptosis (or necroptosis) or are dead. *Green Box*: the duration of the presence of an inhibitor of the IRS. *Panel* (**A**): The initial state of the levels of AβPP-derived *i*Aβ in individual neurons of a healthy person. They are all sub-T1. *Panel* (**B**): Evolution of the initial state in the absence of the drug. AβPP-derived *i*Aβ reaches and crosses the T1 threshold in all affected neurons. The neuronal ISR is elicited and the AβPP-independent *i*Aβ generation pathway activated. Its *i*Aβ product rapidly accumulates, reaches AD pathology-causing levels, and eventually crosses the T2 threshold. As more and more neurons commit apoptosis, the disease enters its end stage. *Panel* (**C**): Evolution of the initial state in the presence of an ISR-inhibiting drug. The effect manifests only following the T1 crossing. The elicitation of the ISR is suppressed. The AβPP-independent *i*Aβ generation pathway remains inoperative. The accumulation of AβPP-derived *i*Aβ continues at the pre-T1 crossing rate. It does not reach AD pathology-causing levels and AD symptoms do not occur for the duration of the treatment.

The effect of the inhibition of the neuronal integrated stress response in the prevention of conventional AD is illustrated in Figure 4. Panel A of Figure 4 shows the initial state of the levels of AβPP-derived *i*Aβ in individual neurons of a healthy person. In all neurons, AβPP-derived *i*Aβ have not yet crossed the T1 threshold, the ISR was not elicited, and the AβPP-independent *i*Aβ production pathway remains inoperative. Panel B of Figure 4 depicts the evolution of the initial state in the absence of any treatment. In this panel, AβPP-derived *i*Aβ crosses the T1 threshold. The eIF2α kinases PKR and/or HRI are activated, eIF2α is phosphorylated at its Ser51, and the neuronal integrated stress response is elicited. Under the ISR conditions, the crucial components required for the operation of the AβPP-independent *i*Aβ generation pathway are produced and the pathway is activated. The entire Aβ output of this pathway remains within the neurons as *i*Aβ, its levels rapidly increase, and AD symptoms manifest. When the levels of *i*Aβ reach and cross the T2 threshold, cells commit apoptosis or necroptosis [1,4]. Panel C of Figure 4 illustrates the effect of suppression of the ISR by an ISR inhibitor such as ISRIB [125] (green box). Since the ISR is elicited only following the crossing of the T1 threshold, this is when the effect of its suppression is manifested. Prior to the T1 crossing, there is no difference between treated and untreated individuals. Following the T1 crossing, the difference is evident. The ISR is suppressed and the AβPP-independent *i*Aβ production pathway remains inoperative. The accumulation of *i*Aβ is supported solely by the influx of AβPP-derived *i*Aβ and it continues at the same rate as prior to the crossing of the T1 threshold for the duration of the treatment. At this rate, AβPP-derived *i*Aβ may not reach AD pathology-causing levels, and AD symptoms may not occur for the duration of the treatment. It should be mentioned that the protective effect of the ISR suppression does not extend to AACD. If the T^0^ threshold were crossed, AACD would commence in the presence of an ISR inhibitor. Since the preventive suppression of the neuronal ISR de facto eliminates the T1 threshold (as far as it applies to the AβPP-independent production of *i*Aβ and, consequently, to the commencement of AD), AACD would persist for the remaining portion of the individual’s lifetime. 

### 10.2. Inhibition of Neuronal ISR in the Treatment of Conventional AD 

**Figure 5:**
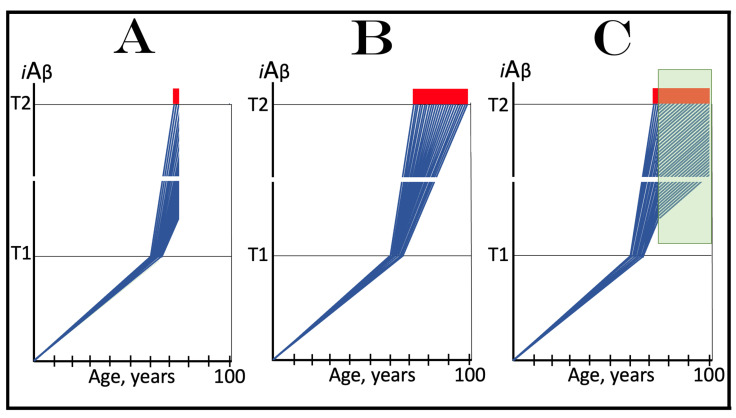
**Effect of inhibition of the neuronal ISR in the treatment of conventional AD***. i*Aβ: intraneuronal Aβ. ***T*1** *threshold*: levels of *i*Aβ that trigger the activation of the eIF2α kinases PKR and/or HRI, elicitation of the neuronal ISR, and initiation of the AβPP-independent production of *i*Aβ. ***T*2** *threshold*: levels of *i*Aβ, produced mainly in the AβPP-independent pathway, which trigger neuronal death. *Blue lines*: levels of *i*Aβ in individual neurons. *Red Box*: apoptotic zone; the fraction of neurons that have committed apoptosis (or necroptosis) or are dead. *Green Box*: the duration of the presence of an inhibitor of the IRS. *Panel* (**A**): The initial state of the levels of AβPP-derived *i*Aβ in individual neurons of a healthy person. In all affected neurons, *i*Aβ has crossed the T1 threshold, the integrated stress response has been elicited, and the AβPP-independent *i*Aβ production pathway has been activated. In a fraction of the neurons, *i*Aβ has crossed the T2 threshold and AD symptoms have manifested. *Panel* (**B**): Evolution of the initial state in the absence of the drug. *i*Aβ, produced mainly independently of AβPP, continues its accumulation unimpeded. Eventually, it crosses the T2 threshold in a sufficient fraction of the neurons and the disease enters its end stage. *Panel* (**C**): Evolution of the initial state in the presence of an ISR-inhibiting drug. With the suppression of the integrated stress response, the supply of components essential for the operation of the AβPP-independent *i*Aβ production pathway ceases and the pathway is disabled. At this point, the neurons that already crossed the T2 threshold are unredeemable, and in a large fraction of the remaining neurons *i*Aβ has reached AD pathology-causing levels. Moreover, the accumulation of AβPP-derived *i*Aβ in the viable neurons continues but at the slow, pre-T1 crossing rate. The progression of the disease would not be stopped but rather would continue at a reduced rate for the duration of the treatment.

The effect of the suppression of the integrated stress response in the treatment of conventional AD is illustrated in Figure 5. Panel A of Figure 5 shows the initial state of *i*Aβ levels in individual affected neurons of the AD patient prior to the administration of the treatment. In all affected neurons, *i*Aβ has crossed the T1 threshold, the integrated stress response has been elicited, and the AβPP-independent *i*Aβ production pathway has been activated. In a fraction of the neurons, *i*Aβ has crossed the T2 threshold and AD symptoms have manifested. Panel B of Figure 5 shows the evolution of the initial state in the absence of the treatment. Accumulation of *i*Aβ, produced mainly in the AβPP-independent *i*Aβ generation pathway, continues unimpeded, and when the T2 threshold is reached and crossed in a sufficient neuronal fraction, the disease enters its end stage. Panel C of Figure 5 depicts the evolution of the initial state in the presence of an ISR inhibitor. With the suppression of the integrated stress response, the production of components essential for the activity of the AβPP-independent *i*Aβ production pathway ceases and the pathway is rendered inoperative. At this point, the over-T2 neurons are either dead or unredeemable and in a large fraction of the sub-T2 neurons *i*Aβ remains at AD pathology-causing levels. Moreover, the accumulation of *i*Aβ in these neurons continues, albeit only in the AβPP proteolytic pathway and at the slow, pre-T1 crossing rate. Therefore, the crossing of the T2 threshold, the consequent neuronal loss, and the progression of the disease would all continue but at a reduced rate. Thus the effect of the ISR suppression in the treatment of conventional AD would be beneficial but limited to only slowing down the progression of the disease.

## 11. Long-Term Suppression of the Integrated Stress Response Is Not Feasible

The preceding section described the beneficial effects, in both the prevention and treatment of conventional Alzheimer’s disease, of the long-term administration of an inhibitor of the integrated stress response. However, the long-term suppression of the ISR is apparently unconceivable and unfeasible. The integrated stress response is a major tool, which is essential for cellular survival [96,97,98,99,100,101,102,103,104,105], and its removal or long-term suppression is bound to cause severe adverse effects. That this is likely the case is strongly suggested by the study of transgenic mice where serine in position 51 of eIF2α was replaced with alanine (eIF2α Ser51Ala KI mice [157]). This substitution has little or no effect on the patterns of cellular transcription and translation, but it prevents the elicitation of the integrated stress response [157]. Indeed, as was mentioned above, the ISR is “integrated” because a multitude of various stressors capable of eliciting it converge on a single “integration” event, namely phosphorylation of eIF2α at its Ser51. This event is, in fact, the core of the ISR, which ushers it in by causing a radical re-programming in both cellular transcription and translation. If the phosphorylation at this position were disabled, no ISR could be elicited. This is precisely what is achieved by the serine to alanine substitution in position 51 of eIF2α. Alanine in this position cannot be phosphorylated by the eIF2α kinases. Consequently, no ISR can be elicited in eIF2α Ser51Ala KI mice [157]. These mice, therefore, constitute a predictive model of the effect of long-term suppression of the ISR. What it forecasts is not encouraging because eIF2α Ser51Ala KI mice survived no longer than 18 h following their birth [157]. It appears, therefore, that the long-term suppression of the ISR in the prevention or treatment of AD (or, in fact, in any other application) is not feasible. It could be argued that the transient administration of ISR inhibitors may be a solution for AD prevention and treatment. However, as described in the following section, this is apparently not the case.

## 12. Transient Suppression of the ISR Would Be Ineffective in Both the Prevention and Treatment of Conventional AD 

**Figure 6:**
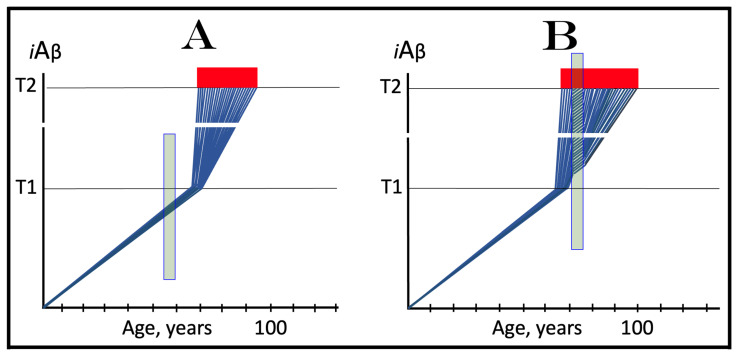
**Effect of transient inhibition of the neuronal ISR in the prevention and treatment of conventional AD***. i*Aβ: intraneuronal Aβ. ***T*1** *threshold*: levels of *i*Aβ that trigger the activation of the eIF2α kinases PKR and/or HRI, elicitation of the neuronal ISR, and initiation of the AβPP-independent production of *i*Aβ. ***T*2** *threshold*: levels of *i*Aβ, produced mainly in the AβPP-independent pathway, which trigger neuronal death. *Blue lines*: levels of *i*Aβ in individual neurons of a person. *Red Box*: apoptotic zone; the fraction of neurons that have committed apoptosis (or necroptosis) or are dead. *Green Boxes*: the duration of the presence of an inhibitor of the IRS. *Panel* (**A**): The transient treatment with the ISR-inhibiting drug is administered prior to the T1 crossing by AβPP-derived *i*Aβ. At this stage, the ISR has not yet been elicited and the administration of its inhibitors serves no purpose and does not affect the continuous accumulation of AβPP-derived *i*Aβ. When the T1 threshold is crossed, the ISR is elicited, the AβPP-independent *i*Aβ production pathway is activated, and AD progresses unimpeded. *Panel* (**B**): The transient ISR suppression treatment is administered when the AβPP-independent *i*Aβ generation pathway is operational and depends on the occurrence of the ISR. Inhibition of the latter disables the former. *i*Aβ continues to accumulate for the duration of the treatment due to the influx of the AβPP-derived *i*Aβ. This accumulation occurs at the slow pre-T1 crossing rate and so does the progression of the disease. When the drug is withdrawn, the ISR is re-elicited (due to over-T1 levels of *i*Aβ) and the AβPP-independent *i*Aβ production pathway re-activated. Both the *i*Aβ accumulation and the progression of AD resume at the fast pre-treatment rate. The transient ISR suppression provides only a short reprieve of slow progression of AD lasting no more than the duration of the treatment.

As concluded in the preceding section, the long-term administration of ISR inhibitors appears unfeasible. The present section analyzes effects of the transient administration of ISR inhibitors in the prevention and treatment of conventional Alzheimer’s disease. As illustrated in Figure 6, ISR suppression treatment is administered for only a limited duration (green boxes). In panel A of Figure 6, it is dispensed to a healthy individual preventively, i.e., when the levels of AβPP-derived *i*Aβ have not yet reached the T1 threshold. At this stage, however, there is no integrated stress response and administration of an ISR inhibitor serves no purpose whatsoever and would produce no effect, at least no beneficial effect. AβPP-derived *i*Aβ will continue to accumulate unimpeded. When it crosses the T1 threshold, the ISR would be elicited, the AβPP-independent *i*Aβ generation pathway activated, the T2 threshold eventually crossed, and the disease would enter the end stage. 

In panel B of Figure 5, the transient ISR suppression treatment is administered to a symptomatic AD patient. At the time of the treatment, AβPP-derived *i*Aβ has crossed the T1 threshold in all affected neurons. The ISR has been elicited and the AβPP-independent *i*Aβ generation pathway activated. Consequently, *i*Aβ, produced mainly in the AβPP-independent pathway and retained intraneuronally, is rapidly accumulating; a fraction of the neurons have crossed the T2 threshold and AD symptoms have manifested. Suppression of the integrated stress response at this stage would abrogate the production and availability of components essential for the activity of the AβPP-independent *i*Aβ generation pathway and the operation of this pathway would cease. However, levels of *i*Aβ would not decline. On the contrary, they would continue to increase, albeit at a low pre-T1 crossing rate, supported only by the influx of AβPP-derived *i*Aβ. The progression of AD would slow down, but only for the duration of the treatment. When, following the transient treatment, an ISR inhibitor is withdrawn, *i*Aβ levels in all affected neurons are well above the T1 threshold. The ISR conditions would be restored, the availability of the essential components re-established, and the AβPP-independent *i*Aβ production pathway re-activated. The rapid accumulation of *i*Aβ would resume, and the disease would progress at the pre-treatment rate. Thus, the transient administration of the ISR suppression treatment would provide only a short reprieve of slow progression of AD lasting no more than the duration of the treatment.

## 13. Depletion of *i*Aβ Can Be Highly Effective in the Prevention and Treatment of Conventional AD

The present study considers two potential therapeutic strategies for Alzheimer’s disease. One is the suppression of the neuronal integrated stress response. This strategy was considered in the preceding sections and, although intuitively promising, was found wanting. Another potential therapeutic strategy for AD is no less intuitive. It is the depletion of intraneuronal Aβ, *i*Aβ. Indeed, in the ACH2.0 paradigm AD pathology is driven by *i*Aβ at sufficiently high levels supported by its production in an AβPP-independent manner. Hence, the intuitive solution: deplete *i*Aβ and you will abolish the driving agent of the disease. But the depletion of *i*Aβ will do more than this. Whereas in conventional AD, the disease is caused by AβPP-derived *i*Aβ accumulated to levels sufficient to activate the eIF2α kinases PKR and/or HRI and thus trigger the elicitation of the neuronal integrated stress response, the AβPP proteolytic pathway alone cannot support accumulation of *i*Aβ to AD pathology-causing levels. The latter is accomplished by the AβPP-independent *i*Aβ generation pathway. Its entire Aβ output is retained within the neurons as *i*Aβ, thus enabling its accumulation to AD pathology-causing levels; in fact, AD commences only when the AβPP-independent *i*Aβ production pathway is rendered operational by the elicitation of the neuronal integrated stress response [1,4]. The neuronal ISR, in turn, is sustained by *i*Aβ at the levels over the T1 threshold. The operation of the AβPP-independent *i*Aβ production pathway ascertains that cellular concentrations of *i*Aβ are sufficient to propagate the neuronal integrated stress response and to perpetuate its own generation in an AβPP-independent manner [1,2,3,4,5,6,7,8]. Therefore, if *i*Aβ were depleted to the levels below the T1 threshold, this not only would deprive the disease of its driver and stop its progression, but would also cease the operation of its major producer, namely the AβPP-independent *i*Aβ generation pathway. Equally intuitive is the preventive application of the *i*Aβ depletion: deplete AβPP-derived *i*Aβ before it reaches the T1 threshold, prevent the T1 crossing (and consequent elicitation of the ISR and activation of the AβPP-independent *i*Aβ production pathway), and you will prevent conventional AD. This notion stems from the ACH2.0 theory of Alzheimer’s disease, but it certainly is not only a theoretical construct. Fortuitously, it has been confirmed physiologically, more than once, by nature, as described in the following section.

## 14. Degradation of *i*Aβ Is a Valid Therapeutic Strategy for Alzheimer’s Disease: Proof of Concept

The notion formulated at the conclusion of the preceding section is verifiable. Indeed, several cleavages within *i*Aβ (or within Aβ segment of AβPP or of C99) occur physiologically and thus cause its degradation (discussed in more detail below). This allows for two sharply defined predictions: 1. The augmentation of the rate of physiologically occurring cleavage within *i*Aβ (i.e., of the degradation of *i*Aβ) would protect from the occurrence of conventional AD; 2. The reduction in the rate of physiologically occurring intra-*i*Aβ cleavage (in other words, in the rate of *i*Aβ degradation) would accelerate the occurrence of AD, i.e., would cause early-onset AD. Both predictions were, in fact, tested by nature and proven to be correct. 

Proof of concept that degradation of intraneuronal Aβ has therapeutic effect in Alzheimer’s disease and the validation of the predictions formulated above come in the form of two naturally occurring mutations of Aβ (reviewed in [4]). One of these mutations affects the intra-*i*Aβ cleaving activity of BACE1, whereas another modifies the intra-*i*Aβ cleaving activity of BACE2. As follows from their designation (BACE stands for beta-site AβPP cleaving enzyme; β-site is a position between residues 671 and 672 of AβPP, and cleavage at this location generates the N-end of Aβ), both BACE1 and BACE2 are capable of cleaving AβPP at the β-site. Both are also capable of cleavages within *i*Aβ (and within Aβ segments of AβPP and of C99). Thus, BACE1 is also competent of cleaving at position 10 of Aβ, a location known as the Aβ10-site or the β’-site [30]. Cleavage at the β-site is the major activity of BACE1, whereas cleavage at the β’-site represents a minor activity of BACE1.

In the case of BACE2, in addition to the cleavage at the β-site, the enzyme is capable of cleaving at positions Aβ19 and Aβ20 (both phenylalanines) [158]. In contrast to BACE1, cleavages at Aβ19 and Aβ20 sites constitute the major activity of BACE2, whereas the cleavage at the β-site represents its minor activity. It appears that both the β’-site cleaving activity of BACE1 and the Aβ19 and Aβ20 site cleaving activities of BACE2 are physiological control mechanisms, possibly “release valves” that regulate and fine-tune the balance between the influx of *i*Aβ and its efflux; consequently, this defines the rate of *i*Aβ accumulation and determines its levels [31,159]. A shift toward the increased efflux of *i*Aβ via the augmentation of the efficiency of the intra-*i*Aβ cleavages would reduce the rate of its accumulation. In terms of the ACH2.0, this would delay or prevent the crossing of the T1 threshold and thus confer protection from AD. The opposite is true for a shift toward the decreased efflux of *i*Aβ via the reduction in the efficiency of the intra-*i*Aβ cleavages. Such a shift would increase the rate of *i*Aβ accumulation, accelerate the crossing of the T1 threshold, and thus cause the early onset of AD. The above considerations provide mechanistic underpinning of the connection between variations in the efficiency of intra-*i*Aβ cleavages by BACE1 and BACE2 and the protection from or causation of AD. Hence, it can be predicted that the increase in the efficiency of intra-*i*Aβ cleavages protects from AD whereas the decrease in the efficiency of intra-*i*Aβ cleavages causes AD. 

The Icelandic Aβ mutation causes an increase in the efficiency of the BACE1 cleavage at the β’-site [30]. The increase is not large, only about 30%. Yet, it is sufficient to borne out the prediction: even a relatively small increase in the efficiency of an intra-*i*Aβ cleavage at the β’-site protects not only from AD but also from aging-associated cognitive decline, AACD [30] (consistent with the nature of AACD defined in Section 8 above and elsewhere [4]).

The Flemish Aβ mutation substitutes the amino acid residue at the position Aβ21 [138]. This position is adjacent to the sites of the major BACE2 cleavage activity (Aβ19 and Aβ20). Consequently, the BACE2-mediated intra-*i*Aβ cleavages at the positions Aβ19 and Aβ20 are suppressed [138]. With the decrease in the efflux of *i*Aβ, its rate of accumulation increases. As predicted, this mutation causes early onset of AD with a mean age of onset at 46 years of age and with the range of the onset starting at the age of 35 years [160,161,162,163,164,165,166]. 

## 15. Activators of BACE1 and BACE2 Are the Prime and Proven Candidates for the Role of Therapeutic *i*Aβ Degradation Agents

In the light of the above discourse, any agent capable of the targeted degradation of *i*Aβ would constitute a potential AD drug. However, the prime candidates for this role are activators of the intra-*i*Aβ cleaving capabilities of BACE1 and BACE2. Indeed, both activities have been proven as effective modulators of Alzheimer’s disease (and of aging-associated cognitive decline). Moreover, the protective effect of the Icelandic Aβ mutation and the AD-causing capability of the Flemish Aβ mutation are not the only indications of the potential benefits of the increased efficiency of intra-*i*Aβ cleavages by BACE1 and BACE2. Thus, overexpression of BACE1 in model systems substantially increased the rate of cleavage at the position Aβ10, shifted the ratio of the intact Aβ to β’-site-cleaved Aβ toward the latter, and decreased deposition of Aβ in brains of transgenic animals [167,168,169,170]. Furthermore, in addition to the cleavage at the position Aβ10, BACE1 is also capable of cleaving *i*Aβ at the position Aβ34 [171,172,173,174], thus generating an intermediate in the *i*Aβ clearance process. In the case of BACE2, on the other hand, its suppression in model systems substantially increased the production of Aβ [159], reflective of the potential of its activation to significantly reduce the generation of *i*Aβ by increasing its degradation. The notion that the intra-*i*Aβ cleaving capability of BACE2 is beneficial and its deficit is detrimental is also supported by studies of brain organoids derived from human pluripotent cells [175]. In these studies, BACE2 activity protected the organoids from Aβ-mediated neuronal loss, whereas its deficit was associated with AD pathology [175]. Interestingly, the deficit of BACE2 activity was also associated with symptoms of Hirschsprung disease in this model system [175].

Even a relatively small increase in the efficiency of a minor activity of BACE1 (i.e., 30% higher efficiency of cleavage at the β‘-site) has a profound protective effect as seen in the Icelandic Aβ mutation carriers. This leaves a large room for improvement in its therapeutic utilization since suitable activators can potentially enhance the intra-*i*Aβ cleaving activity of BACE1 (at both β’ and Aβ34 sites) to extents significantly larger than 30%. Activation of BACE2 for the purposes of the degradation of *i*Aβ is even more promising because the cleavages within *i*Aβ are its major activity. Activation of either BACE1 or BACE2 (more precisely, of their intra-*i*Aβ cleaving capabilities) may be sufficient to achieve a meaningful therapeutic outcome for AD. For maximal efficiency, BACE1 and BACE2 could be activated concurrently. This would potentially produce a strong synergetic effect, not only because these enzymes target diverse sites within *i*Aβ (Aβ10 and Aβ34 in case of BACE1, and Aβ19 and Aβ20 in case of BACE2), but also because BACE1 and BACE2 are apparently localized in different (albeit overlapping) cellular compartments [176]; this would minimize the competition for and maximize the access to their targets. It is important to note that the hypothesis that BACE1 and BACE2 can be utilized as *i*Aβ degradation agents in symptomatic AD presumes that both are produced in neurons under ISR conditions. This subject is further discussed in Section 36 below.

## 16. Effect of Long-Term *i*Aβ Degradation Therapy in the Prevention of Conventional AD

**Figure 7:**
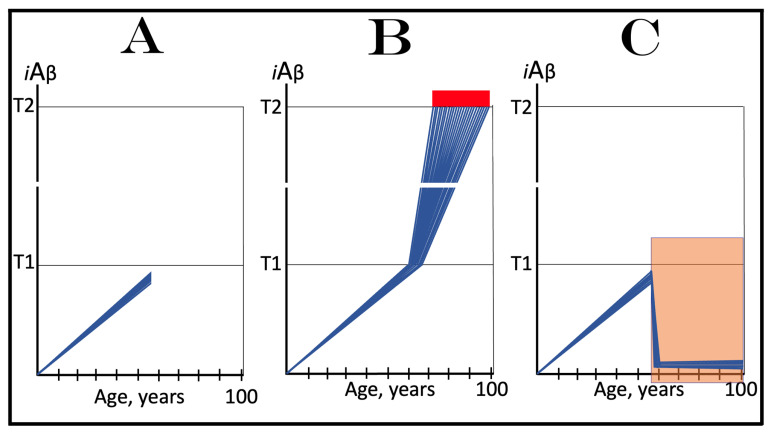
**Effect of long-term iAβ degradation therapy in the prevention of conventional AD***. i*Aβ: intraneuronal Aβ. ***T*1** *threshold*: levels of *i*Aβ that trigger the activation of the eIF2α kinases PKR and/or HRI, elicitation of the neuronal ISR, and initiation of the AβPP-independent production of *i*Aβ. ***T*2** *threshold*: levels of *i*Aβ, produced mainly in the AβPP-independent pathway, which trigger neuronal death. *Blue lines*: levels of *i*Aβ in individual neurons of a person. *Red Box*: apoptotic zone; the fraction of neurons that have committed apoptosis or are dead. *Orange Box*: the duration of the presence of activators of BACE1 and/or BACE2. *Panel* (**A**): The initial state of the levels of AβPP-derived *i*Aβ in individual neurons of a healthy individual. In all neurons, these levels are below the T1 threshold. *Panel* (**B**): Evolution of the initial state in the absence of the treatment. AβPP-derived *i*Aβ reaches and crosses the T1 threshold in all affected neurons. The PKR and/or HRI kinases are activated, eIF2α phosphorylated, the neuronal ISR elicited, and the AβPP-independent *i*Aβ generation pathway initiated. Its *i*Aβ product rapidly accumulates, reaches AD pathology-causing levels, and eventually crosses the T2 threshold. As more and more neurons commit apoptosis, the disease enters its end stage. *Panel* (**C**): Evolution of the initial state in the presence of a drug that activates BACE1 and/or BACE2. *i*Aβ is rapidly depleted. Its levels represent equilibrium between its degradation and the continuous influx of AβPP-derived *i*Aβ and are maintained at a low level for the duration of the treatment. The T1 threshold will not be reached, and conventional AD will not occur in the treated individual.

The effect of the long-term *i*Aβ degradation treatment in the prevention of conventional AD is considered in a scenario illustrated in Figure 7. In this scenario, the administration of activators of BACE1 and/or BACE2, or of any other suitable *i*Aβ degradation agent, commences when AβPP-derived *i*Aβ has not yet reached the T1 threshold. Panel A of Figure 7 depicts the initial state of the levels of *i*Aβ in the individual neurons of a person. At this point, the integrated stress response is not yet elicited, the AβPP-independent *i*Aβ generation pathway is inoperative, and *i*Aβ is produced solely in the AβPP proteolytic pathway. Panel B of Figure 7 shows the evolution of the initial state in the untreated individual. AβPP-derived *i*Aβ continues to accumulate and reaches the T1 threshold. The eIF2α kinases PKR and/or HRI are activated, and eIF2α is phosphorylated at its Ser51. This elicits the neuronal integrated stress response. The ISR-reprogrammed cellular translation provides the essential components of the AβPP-independent *i*Aβ production pathway, the pathway is activated, and AD commences. *i*Aβ produced in this pathway rapidly accumulates. When it reaches the T2 threshold in a fraction of the neurons, AD symptoms manifest; when a sufficient neuronal fraction crosses the T2 threshold, the disease enters its end stage. Panel C of Figure 7 illustrates the evolution of the initial stage in the presence of an *i*Aβ-degrading drug. The levels of *i*Aβ rapidly decrease and are maintained at a low level (in equilibrium with the influx of AβPP-derived *i*Aβ) in the presence of a drug. The T1 threshold will not be reached, and the disease will not occur in the treated individual.

## 17. Effect of Long-Term *i*Aβ Degradation Therapy in the Treatment of Conventional AD

**Figure 8:**
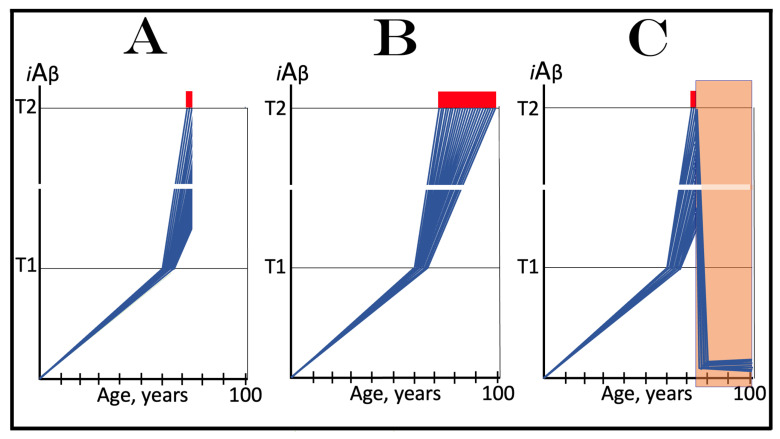
**Effect of long-term iAβ degradation therapy in the treatment of conventional AD***. i*Aβ: intraneuronal Aβ. ***T*1** *threshold*: levels of *i*Aβ that trigger the activation of the eIF2α kinases PKR and/or HRI, elicitation of the neuronal ISR, and initiation of the AβPP-independent production of *i*Aβ. ***T*2** *threshold*: levels of *i*Aβ, produced mainly in the AβPP-independent pathway, which trigger neuronal death. *Blue lines*: levels of *i*Aβ in individual neurons of a person. *Red Box*: apoptotic zone; the fraction of neurons that have committed apoptosis (or necroptosis) or are dead. *Orange Box*: the duration of the presence of activators of BACE1 and/or BACE2. *Panel* (**A**): The initial state of the levels of AβPP-derived *i*Aβ in individual neurons of a healthy person. In all affected neurons, *i*Aβ has crossed the T1 threshold, the integrated stress response has been elicited, and the AβPP-independent *i*Aβ production pathway has been activated. In a fraction of the neurons, *i*Aβ has crossed the T2 threshold and AD symptoms have manifested. *Panel* (**B**): Evolution of the initial state in the absence of the drug. The accumulation of *i*Aβ, produced predominantly independently in the AβPP-independent pathway, continues unimpeded. Eventually, it crosses the T2 threshold in a sufficient fraction of the neurons and the disease enters its end stage. *Panel* (**C**): Evolution of the initial state in the presence of the BACE1- and/or BACE2-activating drug. *i*Aβ is being degraded by the drug in the viable neurons and its levels rapidly decrease (provided that activated BACE1 and/or BACE2 are capable of such efficient *i*Aβ degradation; see Section 23 below). When they are below the T1 threshold, the ISR conditions cease and the AβPP-independent *i*Aβ generation pathway is rendered inoperative. The levels of *i*Aβ decrease further and are maintained at a low equilibrium with the influx of AβPP-derived *i*Aβ by the drug. Neither is the T1 threshold reached nor does AD resume for the duration of the treatment.

The effect of long-term *i*Aβ degradation therapy in the treatment of symptomatic AD patient is considered in a scenario depicted in Figure 8. Panel A of Figure 8 shows the initial state of the levels of *i*Aβ in individual neurons of the AD patient. AβPP-derived *i*Aβ has reached and crossed the T1 threshold in all affected neurons. Consequently, the neuronal integrated stress response has been elicited and the AβPP-independent *i*Aβ generation pathway activated. *i*Aβ produced in this pathway has rapidly accumulated, reached the T2 threshold in a fraction of the neurons, and AD symptoms have manifested. Panel B of Figure 8 illustrates the evolution of the initial state in the untreated patient. The production of *i*Aβ in the AβPP-independent pathway persists uninterrupted. *i*Aβ continues to rapidly accumulate and drives AD pathology. When the T2 threshold is crossed in a sufficient fraction of the neurons, the disease enters its end stage. Panel C of Figure 8 shows the evolution of the initial stage in the AD patient receiving the *i*Aβ-degrading therapy. *i*Aβ is being degraded by the drug in the viable neurons and its levels rapidly decrease (provided that activated BACE1 and/or BACE2 are capable of such efficient *i*Aβ degradation; see more on this subject in Section 23 below). When they are below the T1 threshold, the AβPP-independent *i*Aβ generation pathway is rendered inoperative. The levels of *i*Aβ decrease further and are maintained at a low level by the drug. Neither is the T1 threshold reached nor does AD resume for the duration of the treatment.

## 18. Effects of Transient *i*Aβ Degradation Therapy in the Prevention and Treatment of Conventional AD

The considerations outlined in the preceding section open up a tantalizing possibility, namely the transient, once-in-a-lifetime-only preventive or curative treatment of Alzheimer’s disease. Indeed, if the depletion of *i*Aβ by activated BACE1 and/or BACE2 or by any other suitable *i*Aβ degradation agent is as efficient as shown in the two preceding figures, a substantial reduction of its level, well below the T1 threshold, can be accomplished not by the long-term treatment but rather by the transient administration of a drug. Following such depletion, the accumulation of *i*Aβ would resume de novo. It would be supported solely by AβPP proteolysis. With only this pathway operational, AβPP-derived iAβ does not reach the T1 threshold within the lifetime of the majority of individuals, and even in cases of sporadic AD it crosses the T1 and triggers the disease only after several decades of accumulation. Thus, if following the transient treatment *i*Aβ is depleted deep enough and resumes its accumulation from a sufficiently low threshold, it would take decades for its levels to reach or to be restored to the T1 threshold, and since the T1 crossing by AβPP-derived *i*Aβ is a prerequisite for conventional AD [1,2,3,4,5,6,7,8], the disease would not occur or recur in the treated individuals within their lifetimes.

**Figure 9:**
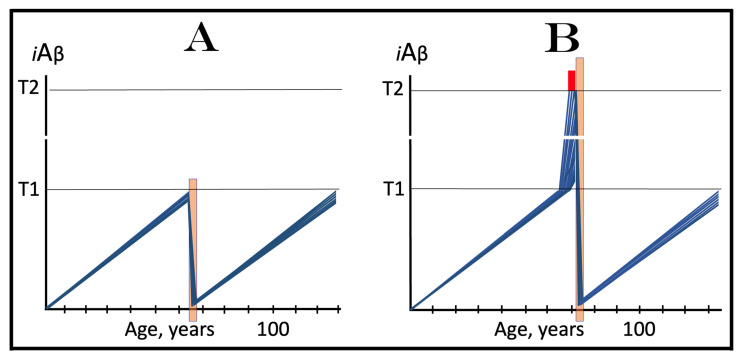
**Effects of the transient activation of BACE1 and/or BACE2 in the prevention and treatment of conventional AD***. i*Aβ: intraneuronal Aβ. ***T*1** *threshold*: levels of *i*Aβ that trigger the activation of the eIF2α kinases PKR and/or HRI, elicitation of the neuronal ISR and initiation of the AβPP-independent production of *i*Aβ. ***T*2** *threshold*: levels of *i*Aβ, produced mainly in the AβPP-independent pathway, which trigger neuronal death. *Blue lines*: levels of *i*Aβ in individual neurons. *Red Box*: apoptotic zone; the fraction of neurons that have committed apoptosis (or necroptosis) or are dead. *Orange Box*: the duration of the presence of activators of BACE1 and/or BACE2. *Panel* (**A**): The transient treatment with the BACE1- and/or BACE2-activating drug is administered prior to the T1 crossing by AβPP-derived *i*Aβ. Following the activation of BACE1 and/or BACE2, *i*Aβ is rapidly depleted. When the drug is withdrawn, the de novo accumulation of AβPP-derived *i*Aβ resumes from a low baseline at the pre-treatment rate. Its levels would not reach the T1 threshold and conventional AD would not occur within the lifetime of the treated individual; *Panel* (**B**): The transient treatment with the BACE1- and/or BACE2-activating drug is administered to a symptomatic AD patient. At the time of the treatment, *i*Aβ has crossed the T1 threshold in all affected neurons. The ISR has been elicited and the AβPP-independent *i*Aβ generation pathway activated. Its *i*Aβ product has rapidly accumulated, reached AD pathology-causing levels, crossed the T2 threshold in a fraction the neurons, and AD symptoms have manifested. Provided the activated BACE1 and/or BACE2 are sufficiently efficient in the degradation of *i*Aβ (see Section 23 below), its levels are rapidly reduced. When the drug is withdrawn, the AβPP-independent *i*Aβ production pathway is no longer operational. The de novo accumulation of AβPP-derived *i*Aβ resumes from a low baseline at the pre T1-crossing rate. The T1 threshold would not be reached and AD would not recur within the lifetime of the treated patient.

The above reasoning is illustrated in Figure 9. In panel A of Figure 9, the transient *i*Aβ degradation therapy is administered preventively, when the levels of AβPP-derived *i*Aβ have not yet reached the T1 threshold. Following the treatment, *i*Aβ is substantially depleted and its accumulation resumes de novo from a low baseline, supported solely by the cellular uptake of secreted AβPP-derived Aβ and by the retention of a fraction of Aβ resulting from the processing of AβPP on the intraneuronal membranes. Its levels would not reach the T1 threshold and AD would not occur within the lifetime of the treated individual.

In panel B of Figure 9, the transient *i*Aβ depletion therapy is administered curatively, to a symptomatic AD patient. At this stage, all affected neurons have already crossed the T1 threshold. The eIF2α kinases PKR and/or HRI have been activated, eIF2α has been phosphorylated, and the integrated stress response has been elicited in neuronal cells. As a result, the essential components of the AβPP-independent *i*Aβ generation pathway have been made available under the ISR conditions and the pathway has been activated. The rate of *i*Aβ accumulation has drastically increased, a fraction of the neurons crossed the T2 threshold, and AD symptoms have manifested. Provided the treatment is sufficiently effective in depleting *i*Aβ, its levels are substantially decreased. Since now they are well below the T1 threshold, the operation of the AβPP-independent pathway of *i*Aβ generation ceases and its accumulation resumes de novo, supported only by AβPP proteolysis. The levels of AβPP-derived *i*Aβ would not reach the T1 threshold and AD would not recur for the remaining portion of the treated patient’s lifetime.

It is highly plausible that transient *i*Aβ depletion via the activation of BACE1 and/or BACE2 (or, potentially, by any other suitable agent) would be effective in the prevention of conventional Alzheimer’s disease when the AβPP-independent *i*Aβ generation pathway is inoperative. This certainty is supported by the observation that an only 30% increase in the efficiency of BACE1-mediated cleavage at the position Aβ10 yields a therapeutically meaningful outcome and protects from AD and AACD, as seen in the carriers of the Icelandic Aβ mutation [30,31]. Potentially, with proper activators, the efficiency of BACE1 cleavages at the intra-iAβ positions Aβ10 and Aβ34 may be increased to much greater extents. The activation of the intra-*i*Aβ cleaving capabilities of BACE2 holds even greater potential. This is because cleavages at positions Aβ19 and Aβ20 are the major activity of BACE2, in contrast to cleavages at positions Aβ10 and Aβ34 being only a minor activity of BACE1.

It can be expected with considerable confidence, therefore, that one-time-only transient depletion of *i*Aβ by the activators of BACE1 and/or BACE2 or by any other suitable agent capable of the targeted degradation of *i*Aβ, administered to healthy individuals at mid-life, would protect from both conventional Alzheimer’s disease and aging-associated cognitive decline for the remaining portion of their lifespan.

On the other hand, there is no confidence in the outcome of *i*Aβ degradation therapy, either transient or long-term, in the treatment of symptomatic AD when the AβPP-independent *i*Aβ generation pathway is operational. It is highly possible that the best that could be attained in this case is the reduction in the rate of progression of the disease. This is because the rate of *i*Aβ production in the AβPP-independent pathway is apparently such (potentially orders of magnitude higher than in the AβPP proteolytic pathway) that the influx of *i*Aβ cannot be matched or exceeded by its targeted degradation via the activation of BACE1 and/or BACE2 or otherwise. To understand the reasons for this assertion and to evaluate the available options, we should make a brief detour and consider the mechanisms potentially underlying the operation of the AβPP-independent *i*Aβ generation pathway.

## 19. Potential Mechanisms Enabling the AβPP-Independent *i*Aβ Generation Pathway

### 19.1. Singular Position of the AUG Encoding Met671 of Human AβPP Suggests Its Possible Physiological Function

Several potential mechanisms capable of generating Aβ independently of AβPP are described below and elsewhere [1,2,3,4,5,6,7,8]. These mechanisms have one common attribute: in all, translation initiates from the AUG normally encoding Met 671 of human AβPP. The rationale for this notion is the following. In 1987, several research groups succeeded in cloning and sequencing the human AβPP cDNA [177,178,179]. Soon thereafter, two investigators, Breimer and Denny [180], noticed that a segment of the gene encoding the C99 fragment of AβPP, the immediate precursor of Aβ in the AβPP proteolytic pathway, is preceded contiguously and in-frame by the AUG codon [180]. They also noted that, for two reasons, this location is singular within the AβPP gene. First, the AUG in question is located within the optimal translation initiation context (Kozak consensus sequence, or Kozak motif). Second, there are 20 methionine-encoding AUG codons within the human AβPP gene. Of those, only the AUG encoding Met671 is situated within the optimal translation initiation context. Even the conventional translation-initiating AUG encoding Met1 of human AβPP is located in a non-optimal translation initiation context [180]. Breimer and Denny speculated that the singularity of the placement of the AUG in question reflects its additional physiological function (beside encoding Met671 of AβPP). They posited that in Alzheimer disease this AUG could be used for the internal initiation of translation within the intact AβPP mRNA, thus leading to the generation of Aβ independently of AβPP [180].

### 19.2. Internal Initiation of Translation of the Intact Human AβPP mRNA from the AUG Encoding Met671 Was Erroneously Ruled Out and Remains a Viable Option

The internal initiation of translation of the intact human AβPP mRNA is only one of the four potential mechanisms capable of generating Aβ independently of AβPP (the other three mechanisms are described below). Following its formulation, Breimer and Denny’s proposal attracted substantial attention and two research groups ventured to test it [181,182]. Both used the same rationale: if translation were initiated internally, modifications within the AβPP gene upstream from the presumed internal initiation site would not interfere with it. Accordingly, one group introduced frame-shifting mutations upstream from the AUG encoding Met671 of human AβPP [181], whereas another group inserted a translation stop-codon upstream from the AUG under discussion [182]. The constructs were transfected into cultured cells and their transient expression was analyzed. Both types of modification would prevent generation of Aβ in the AβPP proteolytic pathway (by preventing the generation of AβPP in the first place). Therefore, it was reasoned, the detection of newly synthesized Aβ would indicate the occurrence of the internal initiation of translation from the AUG in question. Both studies failed to detect newly synthesized Aβ, and both concluded that internal initiation of translation from the AUG encoding Met671 of AβPP could be ruled out. However, this conclusion (in both studies) is intrinsically erroneous. The internal initiation of translation at the AUG encoding Met671 of human AβPP was proposed to occur in AD-affected human neuronal cells [180]. Neither human cells nor, certainly, AD-affected neurons were employed in either study under discussion [181,182]. This renders the “ruling out” conclusions completely irrelevant to the subject. Thus, the proposal made in [180] remains a viable option and needs to be re-evaluated in an adequate model of AD such as that described in [7].

### 19.3. AβPP-Independent Generation of Aβ from 5′-Truncated AβPP mRNA Where the AUG Encoding Met671 of AβPP Becomes the First Translation Initiation Codon

In the remaining three potential mechanisms of the AβPP-independent production of *i*Aβ, translation initiates from the same AUG encoding Met671 of AβPP. However, in these mechanisms, translation occurs not from the intact but rather from severely 5′-truncated AβPP mRNA, where the AUG in question becomes the first, conventionally positioned, translation initiation codon and, accordingly, encodes Met1 of the primary translation product. The principal function of each of these three potential mechanisms is to generate suitably 5′-truncated AβPP mRNA, and each mechanism is defined by its specific way of fulfilling this function. In one of these potential mechanisms, 5′-truncated AβPP mRNA is generated by the internal site-specific initiation of transcription within the AβPP gene. This would require the occurrence of a specific transcription factor, or a co-factor presumably produced in neuronal cells under the ISR conditions. Another potential mechanism is a site-specific cleavage within the intact AβPP mRNA upstream from the AUG in question. This would require the presence of a specialized nuclease produced in the neuronal cells as a result of the ISR-triggered transcriptional/translational reprogramming. 5′-truncated AβPP mRNA produced by the internal initiation of transcription of the AβPP gene and by a site-specific cleavage within the intact AβPP mRNA would be conceptually identical; in each, the AUG under discussion would be the first, 5′-most translation initiation codon. They would be, nevertheless, distinguishable because whereas the former would be 5′ capped, the latter would be uncapped (but translatable under the ISR conditions).

The final potential mechanism is the chimeric RNA-dependent asymmetric amplification of AβPP mRNA. Its 5′-truncated AβPP mRNA end product would be distinguishable in that it would contain the antisense segment within its 5′ untranslated region (5′UTR), hence it would be a “chimeric” molecule. Of the four potential mechanisms of AβPP-independent production of *i*Aβ, its generation via asymmetric amplification of AβPP mRNA is the most plausible; it is supported by empirical data and it explains why AD occurs in humans but neither in other species nor in transgenic animal AD models. Due to its potential importance, this mechanism is considered in some detail below.

## 20. General Principles of Mammalian RNA-Dependent mRNA Amplification

Mammalian RNA-dependent mRNA amplification [183,184,185,186,187,188,189,190,191,192,193] was shown to occur physiologically in circumstances that require greatly increased production of a specific protein(s). It is exemplified by the orders-of-magnitude increased production of alpha and beta globins during terminal erythroid differentiation [184,185] and by the prodigious overproduction of extracellular matrix proteins during their deposition [186]. In this process, every conventionally gene-transcribed mRNA molecule serves repeatedly as a template for the production of “amplified” mRNA molecules. As described below, the mRNA product of the amplification process cannot serve anymore as the template for further amplification, and the amplification process is, therefore, linear. Nevertheless, it greatly increases the production capacity of the gene. Relatively abundant mRNA species can number in the hundreds or thousands conventionally produced molecules per cell. In the mRNA amplification process, each conventionally produced mRNA molecule is analogous to the gene in that it repeatedly directs synthesis of new mRNA molecules. Thus, mammalian RNA-dependent mRNA amplification is an equivalent of a massive, hundreds or thousands times, gene amplification, and can result in an equally massive increase in production of a specific polypeptide.

**Figure 10:**
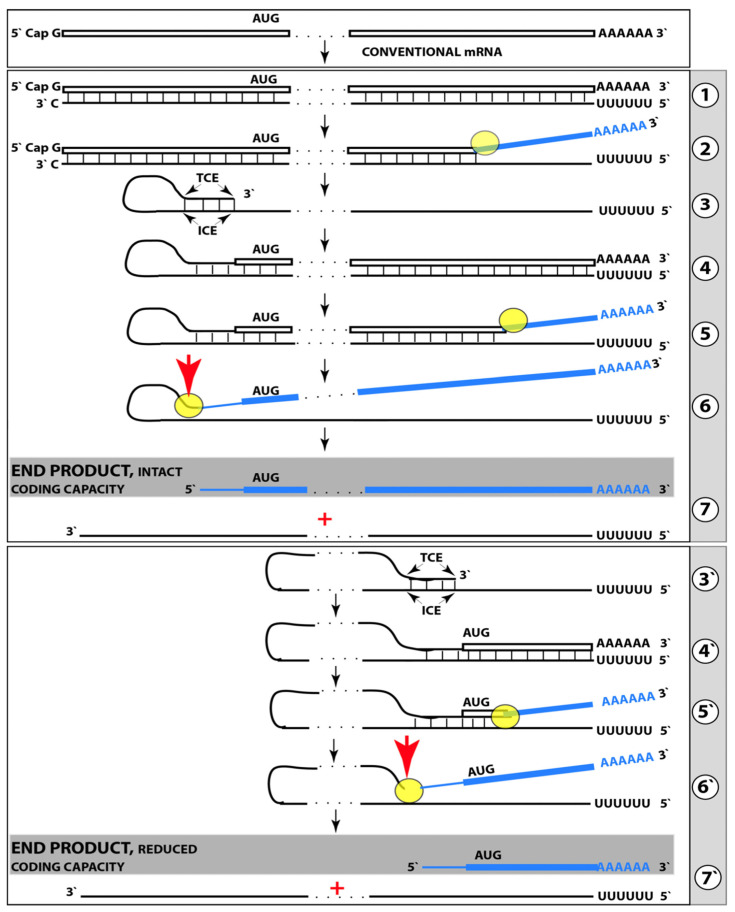
**Principal stages of mammalian RNA-dependent mRNA amplification.** *Boxed line*: sense RNA. *Single line*: antisense RNA. “*AUG*”: translation-initiating codon. “*TCE*”: 3′-terminal complementary element of the antisense RNA; “*ICE*”: internal complementary element of the antisense RNA. *Yellow circle*: enzymatic complex comprised of helicase, nucleotide-modifying activity, and nucleotide-cleaving activity. *Blue lines*: RNA molecules following their separation by the helicase-containing complex. *Red arrows*: location of the cleavage of the chimeric RNA intermediate by the helicase complex. (**Top panel**): conventionally genome-transcribed mRNA serves as the “progenitor” in the mRNA amplification process. (**Middle panel**): Principal stages of the “chimeric” pathway of RNA-dependent amplification of mammalian mRNA. Stage **1**: RdRp transcribes antisense complement from the progenitor mRNA template. Stage **2**: Separation of sense and antisense RNA. The helicase complex binds the 3′-terminal poly(A) component of the double-stranded structure and moves along the sense strand separating it from antisense RNA and modifying about every fifth nucleotide. Stage **3**: separated antisense RNA folds into a self-priming structure; TCE/ICE interaction is essential in this process. Stage **4**: self-primed antisense RNA is extended by RdRp; the resulting product forms a hairpin-like structure. Stage **5**: Complementary sense and antisense components of the hairpin-like structure are separated by the helicase complex. The helicase complex commences at the 3′-terminal poly(A) of the sense RNA; it introduces nucleotide modifications that presumably prevent the RNA strands from re-annealing. Stage **6**: when the single-stranded portion of the hairpin-like structure is reached, the helicase complex cleaves the chimeric RNA intermediate. Stage **7**: End products of the chimeric mRNA amplification pathway. Importantly, the ICE element is located within a segment of the antisense RNA corresponding to the 5′UTR of the mRNA progenitor; consequently, the chimeric RNA end product contains the intact coding region of the progenitor mRNA. (**Bottom panel**): The ICE is situated within a segment of the antisense RNA corresponding to the coding region of the mRNA progenitor. Accordingly, the chimeric mRNA end product contains only a 3′-portion of the coding region of the mRNA progenitor. In this scenario, the translational outcome is determined by the location of the first translation initiation codon. If it were within the remaining segment of the coding region and in-frame with the protein-coding nucleotide sequence, translation would yield the C-terminal fragment of the polypeptide encoded by the progenitor mRNA. Stages **3′**–**7′** correspond to stages **3**–**7**.

The stages of the “chimeric pathway” (the designation explained below) of mammalian RNA-dependent mRNA amplification are illustrated diagrammatically in the two upper panels of Figure 10. The top panel of Figure 10 depict the initial “progenitor” mRNA, namely the conventionally gene-transcribed mRNA molecule. The middle panel of Figure 10 illustrates various principal stages of the amplification process. In Stage 1, the progenitor mRNA is transcribed by the RNA-dependent RNA polymerase (RdRp). Transcription initiates within the 3′-terminal poly(A) segment of the progenitor mRNA, thus generating the 5′-terminal poly(U) of the antisense RNA strand, and terminates at the 3′ end with the “C”, a complement of the 5′ capG of the progenitor molecule; it results thus in a full-size antisense RNA in a double-stranded configuration with the progenitor mRNA. Following transcription, strands are separated by a helicase activity; separation begins at the 3′-terminal poly(A) segment (Stage 2). Upon separation, the progenitor molecule can be repeatedly re-used as a template for additional amplification rounds.

For the further processing of the antisense RNA strand, it has to fold into a self-priming structure shown in Stage 3 of Figure 10. The incidence of such folding is conditional upon the occurrence in the antisense RNA strand of two sufficiently complementary fragments [193]. One of those must be 3′-terminal, hence it is designated the terminal complementary element, TCE. Another fragment can potentially be located anywhere within the antisense RNA, hence it is denoted the internal complementary element, ICE. Importantly, just the occurrence of the TCE and ICE within an antisense RNA strand is not sufficient for the formation of a self-priming structure; these elements should also be topologically and thermodynamically compatible and mutually accessible, i.e., able to find each other and interact within the folded molecule.

If and when the self-priming structure is formed, its 3′ end is extended by RdRp into the sense RNA; the extension terminates with the 3′-terminal poly(A) segment, a transcript of the 5′-terminal poly(U) of the antisense RNA strand (Stage 4). The resulting molecule is comprised of covalently attached sense and antisense RNA strands in a hairpin configuration. This molecule is, therefore, chimeric and is referred to as a “chimeric RNA intermediate”. The site of conversion of the antisense RNA strand into sense orientation, i.e., the site of the initiation of extension of the self-primed antisense RNA is referred to as a “chimeric junction”. Thus, the entire mammalian RNA-dependent mRNA amplification process is defined by the occurrences of chimeric RNA molecules. Therefore, it is designated “the chimeric mRNA amplification pathway”. Upon completion of the extension process and formation of the hairpin structure, components of the double-stranded portion of this structure are separated by a helicase (Stage 5). It is the same helicase as invoked above, and separation is initiated at the 3′-terminal poly(A) complemented by the 5′-terminal poly(U). When the helicase reaches the single-stranded portion of the hairpin structure, it cleaves the RNA molecule (Stage 6). The cleavage may occur either at the 3′ end of the loop portion of the hairpin structure or at one of the TCE/ICE mismatches. If the latter occurs, it may lead to a “chimeric junction shift” (provided the 3’-cleaved antisense RNA is still capable of forming a self-priming structure), a complicated process that is discussed elsewhere [7], and to the repeted utilization of the antisense RNA as a self-primed template [7].

The chimeric mRNA amplification pathway results in two end products (Stage 7). One is the antisense RNA. It is truncated at the 3′ terminus. Its cleaved-off portion is either the complete TCE if the cleavage takes place at the 3′ end of the single-stranded hairpin loop, or a part of the TCE, if the cleavage takes place at a TCE/ICE mismatch. In either case, the antisense RNA end product cannot form the self-priming structure and therefore cannot anymore support the chimeric amplification pathway; it constitutes a “dead end” byproduct in the chimeric amplification pathway. Potentially, if polyadenylated at the 3′ end in conjunction with the cleavage in Stage 6, it can serve as a “progenitor” in another type of RNA amplification [191], but this scenario is outside of the scope of the current subject matter.

The second end product is the chimeric mRNA. Its sense-orientation portion is 5′-truncated. The truncation occurs within a segment corresponding to the ICE element of the antisense RNA strand. It also contains at its 5′ terminus a covalently connected portion of the antisense RNA, in fact its portion that was cleaved-off in Stage 6 (either complete TCF or a part thereof); hence, this is a chimeric RNA end product. Its antisense complement would lack the TCE and ICE elements; therefore, it cannot serve as a progenitor of the amplification process. On the other hand, it retains (as shown in Stage 7) the intact protein coding information of the mRNA progenitor and can be translated into a polypeptide identical to that produced conventionally. This is because both TCE and ICE elements of the antisense RNA are located in its portion corresponding to the 5′UTR of the progenitor mRNA. This, however, is only one of multiple possible outcomes.

## 21. Mammalian RNA-Dependent mRNA Amplification Can Produce a 5′-Truncated mRNA Encoding Only the C-Terminal Fragment of the Original Protein

The chimeric mRNA amplification pathway is of great interest and relevance to our subject matter, namely the production of the C-terminal fragment (CTF) of AβPP independently of AβPP, because it can generate a chimeric mRNA 5′-truncated within its coding region and thus encoding only a CTF of the original progenitor mRNA-encoded polypeptide. In the chimeric mRNA amplification process, the TCE element of the antisense RNA strand is by definition 3′-terminal and cannot be located anywhere else. In contrast, the ICE element can be situated anywhere within the antisense RNA. In the scenario considered in the preceding section, the ICE element is located in the portion of the antisense RNA corresponding to the 5′UTR of the progenitor mRNA. Consequently, the resulting chimeric mRNA end product retains the intact coding information and the capacity to produce the entire gene-encoded protein. However, the outcomes of the chimeric mRNA amplification process are potentially multiple and profoundly distinct from the one described in the preceding section (reviewed in [191]).

With respect to the protein-encoding capacity of the chimeric RNA end product of the mRNA amplification process, the translational outcome is defined by two factors. One is the position of the ICE element of the antisense RNA. Another factor is the position of the first, 5′-most functional translation initiation codon within the chimeric mRNA end product. In one scenario (which is discussed in the preceding section and plays out in the observed amplification of mRNA encoding globins and extracellular matrix proteins [184,185,186]), the ICE is located within segment of antisense RNA corresponding to the 5′UTR of the progenitor mRNA, and the same translation initiation codon is utilized in both the amplified and the progenitor mRNAs; the resulting proteins are identical. The present section considers scenario in which the ICE element of the antisense RNA is positioned in its portion corresponding to the coding region of the progenitor mRNA. This scenario is illustrated and analyzed in the bottom panel of Figure 10. Stages 3′ through 7′ are analogous to Stages 3 through 7; the only difference is that the ICE element of the antisense strand is positioned within the portion of the antisense RNA corresponding to the coding region of the progenitor mRNA. Therefore, due to the location of the site of initiation of self-primed extension, only the 3′-terminal portion of the sense RNA comprising only the 3′ segment of the coding region would be generated during the extension process. Subsequent processing of the chimeric intermediate would generate a chimeric mRNA end product that is 5′-truncated within its coding region. The translational outcome in this case would be determined by the location of the first, 5′-most translation initiation codon in the chimeric RNA end product. If it were positioned within the remaining segment of the coding region and in-frame with the protein-coding nucleotide sequence, translation would yield a C-terminal fragment of the polypeptide encoded by the mRNA progenitor. Such RNA-dependent mRNA amplification would be asymmetric because its chimeric mRNA end product would encode only a CTF of the conventionally produced protein.

## 22. Human AβPP mRNA Is Capable of Asymmetric RNA-Dependent Amplification Resulting in a Chimeric mRNA End Product Encoding the C100 Fragment of AβPP

The preceding section established a possibility of the occurrence of the asymmetric RNA-dependent mRNA amplification resulting in the chimeric mRNA end product yielding, upon its translation, only a CTF of the polypeptide encoded by the mRNA progenitor. Within the narrowly defined boundaries of the present discourse, the relevant questions are the following: Is human AβPP mRNA eligible for the asymmetric RNA-dependent amplification process and, if affirmative, can its amplification process produce chimeric mRNA end product where translation would initiate from the AUG encoding Met671 of AβPP? In topologically specific terms, the AUG encoding Met671 of human AβPP is located over two thousand nucleotides downstream from the 5′ end of human AβPP mRNA. Even if the TCE and ICE elements do occur within the antisense AβPP RNA, they would be separated by a similar distance, thus greatly complicating their potential topological compatibility and mutual accessibility. The above questions, therefore, are complex and seemingly difficult to answer. Propitiously, the occurrence of the TCE and ICE elements and their topological compatibility within the antisense strand of AβPP mRNA or, in fact, of any other mRNA can be assessed experimentally.

The experimental approach to evaluate the eligibility of an mRNA for RNA-dependent amplification simulates this process but utilizes RNA-dependent DNA polymerase, RdDp (reverse transcriptase), instead of RdRp. Reverse transcription of an mRNA is initiated within its 3′-terminal poly(A) segment with the help of oligo(T) primer and results in the antisense strand (cDNA). Following cDNA synthesis, its template, mRNA, is removed by RNase H activity, typically occurring in RdDp preparations (unless genetically modified). This situation is analogous to the one shown in Stages 3 and 4 of Figure 10. Indeed, here there is an antisense strand, separated from its mRNA template, and in the presence of an enzyme (RdDp) capable of extending the self-primed antisense structure. If the TCE and ICE elements were present within the antisense strand and if they were topologically compatible and mutually accessible, the antisense strand would form a self-priming structure and its 3′ terminus would be extended into the sense strand by RdDp. A simple size analysis would indicate if the extension occurred, and nucleotide sequencing would define the site of the initiation of extension and determine the TCE and ICE elements.

The assessment process outlined above was actually implemented, although inadvertently, in the case of human AβPP mRNA. As described above, in 1987 multiple research groups obtained the nucleotide sequence of human AβPP cDNA [177,178,179]. Soon thereafter another research group obtained nucleotide sequence of substantially larger human AβPP cDNA, which was apparently extended at its 3′ end [194]. Investigators assumed initially that the template for this cDNA was the correspondingly 5′-extended AβPP mRNA transcribed from a genomic site upstream from the transcription start site (TSS) defined in the preceding studies [177,178,179]. Subsequently, however, genomic sequence of the human AβPP locus was determined [195] and it transpired that the observed 3′ extension of human AβPP cDNA could not have originated from AβPP mRNA whose transcription was initiated upstream from the “normal” TSS and, accordingly, the authors corrected their paper and declared their finding an artifact [194].

However, the subsequent analysis of nucleotide sequence of the 3′-extended human AβPP cDNA observed in [194] demonstrated that the 3′-extension was, in fact, a segment of the sense strand of human AβPP [196,197,198]. Moreover, this analysis lucidly indicated that the additional 3′ segment was generated by a self-primed cDNA extension that occurred during the preparation of human AβPP cDNA precisely as described above. The nucleotide sequence analysis defined the site of the initiation of extension, which is indeed situated about two thousand nucleotides from the 3′ terminus of cDNA, and enabled the identification of the TCE and ICE elements [196,197,198]. These findings established that (a) human AβPP mRNA is eligible for amplification in the RNA-dependent process described above and (b) that such amplification would occur asymmetrically [196]. It also established that the first, 5’-most translation initiation codon within the observed self-primed extension of human AβPP cDNA is the ATG normally encoding methionine in position 671 of AβPP [196].

**Figure 11:**
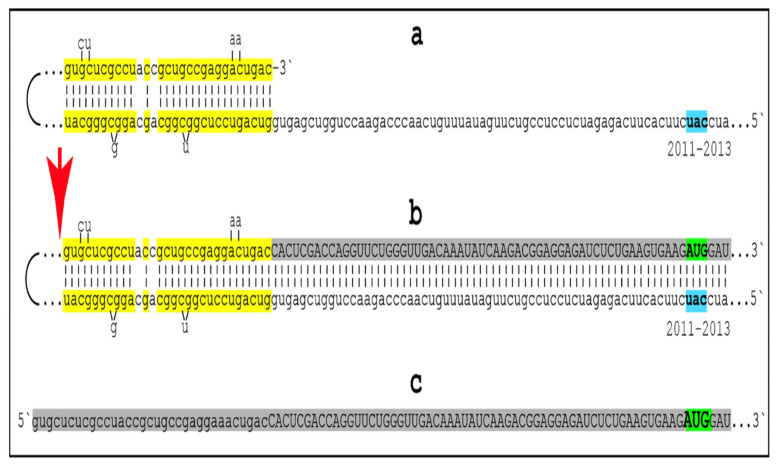
**Human AβPP mRNA is a potential progenitor in the asymmetric RNA-dependent amplification process; the resulting chimeric mRNA end product encodes the C100 fragment of AβPP**. *Small letters*: nucleotide sequences of the relevant segments of the antisense human AβPP RNA. *Capital letters*: nucleotide sequences of the sense human AβPP mRNA segments resulting from the extension of self-primed antisense RNA. Letters highlighted *in yellow*: the TCE (top) and the ICE (bottom) components of the human antisense AβPP RNA. *“2011–2013”*: nucleotide locations (counted from the 3′ end of the antisense AβPP RNA) of the “uac” (highlighted *in blue*), the complement of the “AUG” (highlighted *in green*) encoding Met671 in the human AβPP mRNA. Panels (**a**–**c**) correspond to Stages 3′, 4′, and 6′ of Figure 10. *Panel* (**a**): human antisense AβPP RNA folds into self-priming structure; TCE/ICE interaction is essential in this process. *Panel* (**b**): self-primed human antisense AβPP RNA is extended into a segment (highlighted *in gray*) of the human sense AβPP mRNA. *Red arrow*: when the helicase complex reaches the single-stranded portion of the chimeric hairpin-like structure, the cleavage occurs either at the 3′ end of the loop (shown) or at a mismatch within the TCE/ICE segment. *Panel* (**c**): The chimeric mRNA end product of RNA-dependent amplification of human AβPP mRNA (highlighted *in gray*). It consists of the antisense segment covalently attached to severely 5′-truncated AβPP mRNA. It retains only the 3′ portion of the coding region of the progenitor mRNA. Its first functional translation initiation codon is the AUG encoding Met671. Its translation would result in the C100 fragment of AβPP generated independently of the latter.

The projected folding of human antisense AβPP RNA, separated from its mRNA template, into a self-priming conformation and the sequences and interaction of the anticipated TCE and ICE elements are illustrated in Figure 11. Panels a–c of Figure 11 parallel panels 3′ through 7′ of Figure 10. The TCE and ICE elements are highlighted in yellow. They are indeed separated by about 2000 nucleotides but are apparently mutually accessible and capable of forming a self-priming structure (panel a of Figure 11). The TCE primer is extended and transcription of the 5′-terminal portion of the antisense RNA into its sense RNA complement (highlighted in gray) is completed by the production of the 3′-terminal poly(A) segment (panel b of Figure 11). Following separation of the sense and antisense strands by a helicase, the cleavage would occur either at the 5′ end of the TCE (indicated by the red arrow in Figure 11) or at one of mismatches within the TCE/ICE complex. The resulting chimeric mRNA end product (shown in panel c of Figure 11 and highlighted in gray) terminates at the 5′ end with the TCE element or a portion of the TCE element of the antisense RNA. It contains a severely 5′-truncated coding region of human AβPP mRNA. Its first, 5′-most translation initiation codon is the AUG encoding Met671 of AβPP and positioned within the optimal translation initiation context. Translation of the asymmetrically amplified AβPP mRNA would generate, therefore, the C100 fragment of AβPP independently of AβPP.

As mentioned above (Section 5), the AβPP-independent *i*Aβ generation pathway is inoperative in mice and in transgenic mouse models overexpressing human AβPP and AβPP mRNA [7]. Since mouse cells were shown to possess the RNA-dependent mRNA amplification machinery [183,184,185,186], the reason for this is that the antisense complements of both mouse AβPP mRNA and the exogenously expressed human AβPP mRNA lack the TCE and ICE elements [197,198]. Indeed, segments of mouse antisense AβPP RNA corresponding to the TCE and ICE elements of human antisense AβPP RNA exhibit no more than a random degree of complementarity [197,198]. As for exogenously overexpressed human AβPP RNA, its 5′ termini were heavily modified during construction of the AβPP transgenes thus eliminating the TCE element in the corresponding antisense AβPP RNA molecules and rendering them ineligible for the amplification process.

## 23. Matching the Influx of *i*Aβ Produced in the AβPP-Independent Pathway by Its Targeted Degradation via the Activation of BACE1 and/or BACE2 Could Be a Very Tall Order

The preceding three sections have been introduced to highlight the potential efficiency of the generation of *i*Aβ in the AβPP-independent pathway and to assist in assessing the potential effectiveness of *i*Aβ degradation therapy in the treatment of AD. Above, we have established that, with a substantial degree of certainty, *i*Aβ depletion therapy, via the activation of BACE1 and/or BACE2 or otherwise, would be highly effective in the prevention of conventional AD, when the AβPP-independent *i*Aβ generation pathway is inoperative. The basis for this conclusion is an observation that an only 30% increase in the efficiency of BACE1 cleavage at the β’-site (position Aβ10) is sufficient to prevent both AD and AACD. Potentially, the efficiency of BACE1 cleavages not only at the position Aβ10 but also at Aβ34 can be activated to a significantly greater extent. Even more potent in the depletion of *i*Aβ would be the enhancement in the rate of cleavages at positions Aβ19 and Aβ20, the major activity of BACE2. It should be reemphasized, however, that in the proposed prevention of conventional AD, only AβPP-derived *i*Aβ is being depleted; the AβPP-independent *i*Aβ generation pathway remains inoperative at this stage. To roughly assess what we are up against in the treatment of symptomatic AD, when the AβPP-independent *i*Aβ production pathway is operational, it is instructive to compare the efficiencies of generation of *i*Aβ in the AβPP proteolytic and AβPP-independent pathways. For the purposes of comparison, it can be assumed that probably no more than one tenth of Aβ produced by the AβPP proteolysis ends up as *i*Aβ, whereas, in contrast, the entire Aβ output of the AβPP-independent pathway is retained within the neurons as *i*Aβ [1,2,3,4,5,6,7,8]. This is already an order of magnitude greater efficiency for the latter. In the AβPP proteolytic pathway, a 771 amino acid residues-long precursor needs to be synthesized for every Aβ molecule to be produced. In the AβPP-independent pathway, only a C100 fragment needs to be synthesized for every *i*Aβ to be generated. Here is roughly another order of magnitude difference in efficiencies. Moreover, it is exceedingly plausible that the generation of *i*Aβ in the AβPP-independent manner in Alzheimer’s disease is enabled by the asymmetric RNA-dependent amplification of human AβPP mRNA. This, apparently, appends additional orders of magnitude difference in the efficiency of generation of *i*Aβ by the AβPP proteolysis versus the AβPP-independent pathway. This is because, as argued above, in its efficiency, the mammalian RNA-dependent mRNA amplification process is equivalent to a massive gene amplification in that amplified mRNA is produced from hundreds or even thousands of conventionally gene-transcribed mRNA templates. The bottom line is that the rate of the influx of *i*Aβ produced in the AβPP-independent pathway is potentially many orders of magnitude greater than that of *i*Aβ generated by AβPP proteolysis and could be so substantial that, practically, it cannot be matched, not to speak exceeded, by the targeted degradation of *i*Aβ via the activation of BACE1 and/or BACE2. In other words, BACE1 and/or BACE2 activators could be effective in situations where *i*Aβ is derived only by AβPP proteolysis, but would be inefficient on their own in circumstances involving the production of *i*Aβ in the AβPP-independent pathway.

## 24. Projected Effect of Targeted *i*Aβ Degradation via the Activation of BACE1 and/or BACE2 in the Treatment of Conventional Symptomatic Alzheimer’s Disease

**Figure 12:**
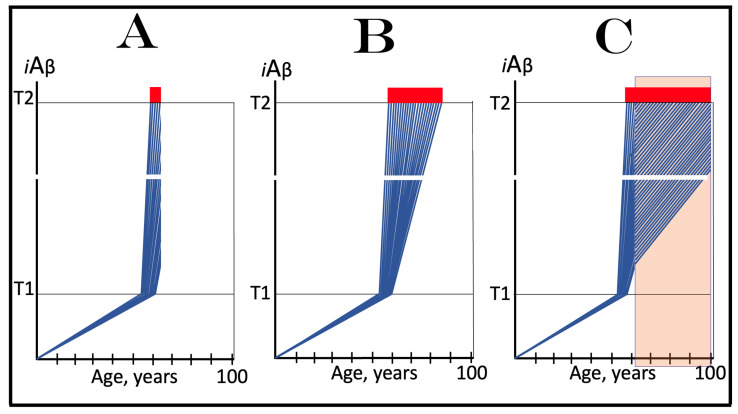
**Projected effect of targeted *i*Aβ degradation via the activation of BACE1 and/or BACE2 in the treatment of conventional symptomatic AD***. i*Aβ: intraneuronal Aβ. ***T*1** *threshold*: levels of *i*Aβ that trigger the activation of the eIF2α kinases PKR and/or HRI, elicitation of the neuronal ISR, and initiation of the AβPP-independent production of *i*Aβ. ***T*2** *threshold*: levels of *i*Aβ, produced mainly in the AβPP-independent pathway, which trigger neuronal death. *Blue lines*: levels of *i*Aβ in individual neurons of a person. *Red Box*: apoptotic zone; the fraction of neurons that have committed apoptosis (or necroptosis) or are dead. *Orange Box*: the duration of the presence of activators of BACE1 and/or BACE2. *Panel* (**A**): The initial state of the levels of AβPP-derived *i*Aβ in individual neurons of a healthy person. In all affected neurons, *i*Aβ has crossed the T1 threshold, the integrated stress response has been elicited, and the AβPP-independent *i*Aβ production pathway has been activated. In a fraction of the neurons, *i*Aβ has crossed the T2 threshold and AD symptoms have manifested. *Panel* (**B**): Evolution of the initial state in the absence of the drug. The accumulation of *i*Aβ, produced mainly independently of AβPP continues unimpeded. Eventually it crosses the T2 threshold in a sufficient fraction of the neurons and the disease enters its end stage. *Panel* (**C**): Evolution of the initial state in the presence of a BACE1- and/or BACE2-activating drug. The rate of degradation of *i*Aβ by activated BACE1 and/or BACE2 does not match that of the influx of *i*Aβ produced in the AβPP-independent pathway. The accumulation of the latter continues although at a reduced rate. The progression of the disease is not stopped but is merely slowed down for the duration of the treatment.

Figure 12 illustrates the effect of the targeted degradation of *i*Aβ via the activation of BACE1 and/or BACE2 or by any other suitable *i*Aβ degradation agent in conventional symptomatic AD in the light of considerations discussed in the preceding section. The present and the following sections consider “the worst case scenario”; namely, that the rate of the influx of *i*Aβ generated in the AβPP-independent pathway is such that it cannot be matched by the rate of the efflux of *i*Aβ via its targeted degradation (potential outcomes of alternative scenarios are reviewed in Section 36 below). Consequently, the best possible outcome of the *i*Aβ depletion therapy would be only a reduction in the rate of *i*Aβ accumulation rather than its reversal; the disease would persist and its progression would continue, albeit at a reduced rate.

Panel A of Figure 12 depicts the initial state of the levels of *i*Aβ in the individual neurons of a symptomatic AD patient at the commencement of the targeted *i*Aβ degradation. At this point, AβPP-derived *i*Aβ has crossed the T1 threshold in all affected neurons. The neuronal integrated stress response has been elicited and the AβPP-independent *i*Aβ generation pathway activated. *i*Aβ produced independently of AβPP rapidly accumulates, reaches AD pathology-causing levels, crosses the T2 threshold in a fraction of the neurons, and AD symptoms manifest. Panel B of Figure 12 shows the evolution of the initial state in the untreated patient. The rapid accumulation of *i*Aβ produced in the AβPP-independent pathway continues unimpeded. It drives AD pathology, propagates the neuronal integrated stress response, and perpetuates the operation of the AβPP-independent *i*Aβ generation pathway [4]. Additional neurons reach and cross the T2 threshold and eventually the disease enters its end stage. Panel C illustrates the evolution of the initial state in the symptomatic AD patient receiving the targeted *i*Aβ degradation therapy. As shown, the rate of the efflux of *i*Aβ caused by its targeted degradation via the activation of BACE1 and/or BACE2 cannot match the rate of the influx of *i*Aβ generated in the AβPP-independent pathway. As the result, the accumulation of *i*Aβ endures, the crossings of the T2 threshold persist, and the progression of the disease continues although at a decreased rate.

## 25. How to Treat Alzheimer’s Disease: Catch-22 and Its Resolution

### 25.1. Catch-22: For the Degradation of iAβ to Be Effective in the Inactivation of the AβPP-Independent iAβ Production Pathway, This Pathway Has to Be First Inactivated!

The effect of targeted *i*Aβ degradation therapy in symptomatic AD, described in the preceding section, is, of course, beneficial but falls short of the declared goal of such therapy, namely stopping the progression of the disease. The initial design of the attempted *i*Aβ depletion therapy in the treatment of symptomatic AD (discussed in Section 17 and Section 18 above), when the AβPP-independent *i*Aβ generation pathway is operational, was to reduce the levels of *i*Aβ to those below the T1 threshold [4,8]. This would render the AβPP-independent *i*Aβ production pathway inoperative. As argued above, it is highly plausible that the targeted *i*Aβ degradation therapy via the activation of BACE1 and/or BACE2 would be very effective when its influx is limited to AβPP-derived *i*Aβ, i.e., when levels of *i*Aβ are below the T1 threshold and the AβPP-independent *i*Aβ production pathway remains inoperative. To be effective in symptomatic AD, such a therapy has first to bring down the levels of *i*Aβ sufficiently (i.e., below the T1 threshold) to de-activate its production in the AβPP-independent pathway; it is apparently incapable of this. However, it would be perfectly capable of this if the influx of *i*Aβ produced independently of AβPP were stopped prior to the commencement of the *i*Aβ degradation therapy. Thus, the attempt to employ the targeted *i*Aβ degradation therapy alone in symptomatic AD faces a classic Catch-22 conundrum: To reduce *i*Aβ levels sufficiently to stop the operation of the AβPP-independent *i*Aβ generation pathway, this pathway should first be inactivated.

### 25.2. Resolution of Catch-22: Transient, ISR Inhibitor-Mediated Suppression of the AβPP-Independent Production of iAβ for the Duration of Its Concurrent Depletion below the T1 Threshold

The only way to resolve this (or, in fact, any other) Catch-22 situation is to bring into the play an additional factor. In this case, it means to first disable the AβPP-independent *i*Aβ generation pathway and to stop the influx of its *i*Aβ product by means other than targeted *i*Aβ degradation via the activation of BACE1 and/or BACE2. With the supply of *i*Aβ produced in this pathway discontinued, only the influx of AβPP-derived *i*Aβ would occur, and it is apparent (see above) that the activation of BACE1 and/or BACE2 would be sufficiently effective in depleting *i*Aβ in this situation. Moreover, the inactivation of the AβPP-independent *i*Aβ generation pathway in concert with the *i*Aβ degradation therapy can be transient rather than long-term. Indeed, it should persist only until levels of *i*Aβ are reduced to those below the T1 threshold. At this point, if the drug used to inactivate the AβPP-independent *i*Aβ production pathway were withdrawn, the pathway would nevertheless remain inactive due to insufficient levels of *i*Aβ and, because of the inactivity of this pathway, the effectiveness of the *i*Aβ depletion therapy would persist.

The question is what could this additional AβPP-independent *i*Aβ production-inactivating factor be? It could, in fact, be any suitable agent capable of interfering with the molecular mechanism underlying the AβPP-independent *i*Aβ production pathway. However, in the context of the present discourse, the answer is apparent: inhibitors of the integrated stress response. Indeed, as deliberated above, the suppression of the neuronal ISR would block the supply of essential components required for the activity of the AβPP-independent *i*Aβ production pathway, and, consequently, would disable its operation. Moreover, whereas, as discussed above, the ISR inhibitors cannot be employed long-term, their transient utilization is feasible. Indeed, the ISR inhibitor ISRIB was successfully utilized transiently to abrogate cognitive impairment in mouse models overexpressing Aβ without any significant adverse effects [125].

**Figure 13:**
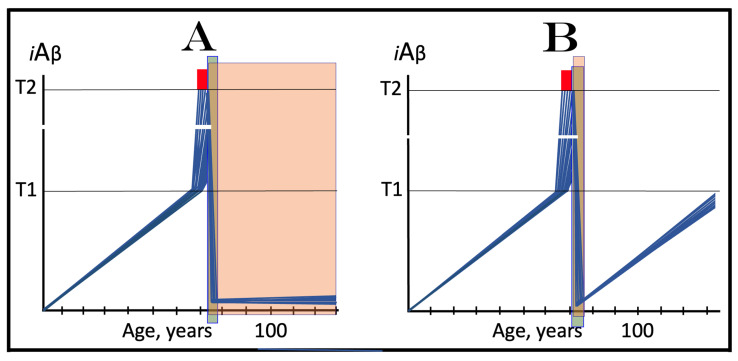
**Composite therapy for conventional symptomatic AD: transient, ISR inhibitor-mediated suppression of the AβPP-independent production of *i*Aβ for the duration of its concurrent, BACE activator-mediated depletion below the T1 threshold**. *i*Aβ: intraneuronal Aβ. ***T*1** *threshold*: levels of iAβ that trigger the activation of the eIF2α kinases PKR and/or HRI, elicitation of the neuronal ISR, and initiation of the AβPP-independent production of *i*Aβ. ***T*2** *threshold*: levels of *i*Aβ, produced mainly in the AβPP-independent pathway, which trigger neuronal death. *Blue lines*: levels of *i*Aβ in individual neurons of a person. *Red Box*: apoptotic zone; the fraction of neurons that have committed apoptosis (or necroptosis) or are dead. *Orange Boxes*: the duration of the presence of activators of BACE1 and/or BACE2. In both panels, by the time the composite therapy commences, AβPP-derived *i*Aβ has crossed the T1 in all affected neurons of an AD patient, the ISR has been elicited, and the AβPP-independent *i*Aβ production pathway has been rendered operational; *i*Aβ has rapidly accumulated, its levels have crossed the T2 threshold in a fraction of the neurons, and AD symptoms have manifested. *Panel* (**A**)**:** The transient administration of an ISR inhibitor overlaps concurrently with the long-term treatment with BACE activators. With the neuronal ISR suppressed, the AβPP-independent *i*Aβ generation pathway is disabled and the influx of its *i*Aβ product ceases. Activated BACE1 and/or BACE2 efficiently and rapidly deplete *i*Aβ to below-T1 levels. At this point, the ISR inhibitor can be withdrawn and the AβPP-independent *i*Aβ production pathway remains inoperative. The depletion of *i*Aβ continues until its levels reach equilibrium with the influx of AβPP-derived *i*Aβ. The levels of *i*Aβ would not be restored to the T1 threshold, the ISR would not be re-elicited, the AβPP-independent *i*Aβ production pathway would not be re-activated, and AD would not recur in the presence of BACE activators. *Panel* (**B**): Similar to panel A except for the duration of treatment with BACE activators. In this panel ISR inhibitors and BACE activators are administered concurrently and transiently. The ISR-inhibiting drug is withdrawn when *i*Aβ is depleted to below-T1 levels, and BACE1- and/or BACE2-activating drugs are removed when *i*Aβ is depleted to the desired low levels. Following the composite therapy, the accumulation of AβPP-derived *i*Aβ resumes de novo, from a low baseline and at the slow pre-T1 crossing rate. It will not reach the T1 threshold and AD will not recur within the remaining lifetime of the treated patient.

A composite concurrent utilization of transient ISR suppression and *i*Aβ depletion therapy in the treatment of symptomatic conventional AD is illustrated in Figure 13. In both panels of Figure 13, the treatment commences when AD symptoms have already manifested. By this time, AβPP-derived *i*Aβ has crossed the T1 in all affected neurons of the AD patient, the ISR has been elicited, and the AβPP-independent *i*Aβ production pathway has been rendered operational; *i*Aβ is rapidly accumulating and its levels have crossed the T2 threshold in a fraction of the neurons. In panel A of Figure 13, the commencement of administration of an ISR inhibitor (green box) is followed concurrently by the start of the *i*Aβ depletion therapy (orange box; the coordination between these two types of treatment is further discussed in the following section). The suppression of the ISR disables the operation of the AβPP-independent *i*Aβ generation pathway. With the influx of *i*Aβ produced independently of AβPP abolished, the *i*Aβ depletion via the activation of BACE1 and/or BACE2, or by any other suitable *i*Aβ depletion agent, is very effective and levels of *i*Aβ are rapidly reduced. When the ISR inhibitor is withdrawn, *i*Aβ levels are already below the T1 threshold, and the AβPP-independent *i*Aβ generation pathway remains inoperative. In the presence of the activator(s) of BACE1 and/or BACE2, the depletion of *i*Aβ continues until a low baseline equilibrium between the influx of AβPP-derived *i*Aβ and its efflux by depletion is established. The levels of *i*Aβ would not reach the T1 threshold, the AβPP-independent *i*Aβ generation pathway would not be re-activated, and AD would not recur for the duration of the *i*Aβ depletion treatment.

It is anticipated that that the depletion of *i*Aβ, carried out in combination with the inhibition of the ISR and, therefore, in the absence of the influx of *i*Aβ produced in the AβPP-independent pathway, would be very effective. In such a case, both treatments of the composite therapy, namely the inhibition of the ISR as well as the depletion of *i*Aβ, could be transient. This scenario is depicted in panel B of Figure 13. This panel is mostly identical to panel A. The only difference is that the *i*Aβ depletion treatment is transient rather than long-term. The inhibitor(s) of the ISR is removed when *i*Aβ levels are below the T1 threshold and the activator(s) of BACE1 and/or BACE2 is withdrawn when *i*Aβ is substantially depleted. Following the treatment, its accumulation resumes de novo from a low baseline. It is supported solely by the AβPP proteolysis and proceeds at the slow, pre-T1 crossing rate. Its levels would not be restored to the T1 threshold, the AβPP-independent *i*Aβ production pathway would not be reactivated, and the disease would not recur within the remaining lifetime of the treated patient. In this scenario, a single composite treatment would stop the progression of AD and potentially cure the disease. It should be emphasized that in both cases, namely long-term and transient treatments with BACE activators, the suppression of the ISR has to be maintained at least until *i*Aβ is depleted below the T1 threshold. At this point, when its inhibitor is withdrawn, the neuronal ISR cannot be re-elicited and the AβPP-independent *i*Aβ production pathway re-activated (if the inhibitor is removed prior to this point, the ISR would be re-established by over-T1 *i*Aβ, and the activity of the AβPP-independent *i*Aβ generation pathway would be re-initiated).

## 26. Orchestration of the Composite AD Therapy

### 26.1. Coordination of the ISR Inhibition and iAβ Depletion Treatments

To maximize the beneficial effect of the composite therapy for symptomatic conventional AD and to minimize its potential detrimental consequences, the administration of both types of drugs, namely inhibitors of the ISR and activators of BACE1 and/or BACE2, should be carefully coordinated. This requires at least approximate knowledge of the basic parameters of the effects of the drugs. First, we need to estimate the duration of a period between the dispensation of an ISR inhibitor and the cessation of the operation of the AβPP-independent *i*Aβ generation pathway. The suppression of the integrated stress response would stop the production of the essential components required for the operation of the AβPP-independent *i*Aβ generation pathway, but its activity would continue until the preexisting components are physiologically degraded. Only when the operation of the AβPP-independent *i*Aβ production pathway has ceased should an *i*Aβ depletion drug be administered. The second parameter is the timing of the withdrawal of the ISR inhibitor. This parameter is important because the ISR inhibitor should, due to its potential deleterious effect, be removed as soon as its presence is no longer required. It is determined by the rate of depletion of *i*Aβ. The ISR inhibitor should be withdrawn when the levels of *i*Aβ have been reduced below the T1 threshold. Operationally, it means that *i*Aβ levels are sufficiently low for the ISR not to be re-elicited and for the operation of the AβPP-independent *i*Aβ production pathway not to resume when the ISR inhibitor is withdrawn. The third parameter is the duration of the administration of the *i*Aβ depletion drug. This would be determined by the time period required to bring the levels of *i*Aβ within the desired range.

All these parameters can be evaluated and approximated in the human neuronal cells-based AD model described in [7]. The third parameter (of the three mentioned above) can be approximated simply by measuring the levels of *i*Aβ at different time points during the treatment and comparing them with the baseline level of regular human neuronal cells. The approximation of the first two parameters is more complex. It requires the determination of the activity of the AβPP-independent *i*Aβ generation pathway. This is feasible because, as discussed below, the primary product of this pathway, the C100 fragment of AβPP and the resulting *i*Aβ are distinguishable from C99 and Aβ generated by AβPP proteolysis.

### 26.2. Detection of the Activity of the AβPP-Independent iAβ Production Pathway: C100 and Aβ Generated Independently of AβPP Are Distinguishable from Their Counterparts Produced by AβPP Proteolysis

When the utilization of the AUG encoding Met671 of human AβPP in the initiation of translation was first proposed [180], it was presumed that its primary product would be C99, identical in all respects to and indistinguishable from C99 produced by proteolysis of AβPP. This was due to the assumption that, as typically occurs during eukaryotic translation, the translation-initiating Met would be removed co-translationally by N-terminal methionine amino peptidase, MAP. However, subsequent studies determined that this is not the case [199,200,201,202,203,204]. Methionine is utilized in the initiation of translation of the majority of cellular proteins. Typically, it is removed by MAP co-translationally, while the process is still ongoing, and thus the amino acid residue following the translation-initiating Met forms the N-terminus of a resulting polypeptide. For the MAP cleavage to occur, the translation-initiating Met as well as the following amino acid residue must fit within the active site of the enzyme. This, however, is not always achievable and depends on the dimensions of the second amino acid residue [200,201,202,203]. Only seven smallest residues can fit together with the N-terminal Met into the active site of MAP. In the order of increasing size, they are the following: Gly, Ser, Cys, Thr, Pro, and Val [199,200,201,202,203,204]. When the N-terminal translation-initiating methionine is followed by any other residue, the MAP-mediated cleavage does not occur, and the translation-initiating Met is retained at the N-terminus of the primary product of translation [199,200,201,202,203,204].

Met 671 of human AβPP is followed by Asp. The Met/Asp combination cannot fit into the MAP active site. Therefore, if translation were initiated at the AUG encoding Met671 of human AβPP (regardless of the underlying mechanism), the translation-initiating Met would not be removed by MAP and the primary translation product would be the C100 fragment of AβPP (i.e., Met-C99) rather than C99. Moreover, the gamma-cleavage of C100 would generate Met-Aβ rather than Aβ. In cases when MAP is unable to remove the translation-initiating Met, the latter is usually cleaved-off by one of numerous cellular amino peptidases with a broad specificity [204]. Thus, eventually, C100 and Met-Aβ would be converted into C99 and Aβ. Importantly, this would take place post-translationally. This is because only MAP is capable of the co-translational removal of the N-terminal Met; all other cellular amino peptidases do it following the termination of translation. Consequently, if the AUG encoding Met671 of human AβPP were utilized in the initiation of translation, both C100 and Met-Aβ should be present in the AD-affected neurons or in the human neuronal cells-based AD model [7,205] (it should be noted that neither would occur in postmortem samples because in dying cells protein synthesis stops long before proteolysis does; without the influx of C100, the conversion of preexisting C100 and Met-Aβ into C99 and Aβ would be complete). Thus, C100 and Met-Aβ can serve as the reporters of the activity of the AβPP-independent *i*Aβ generation pathway. The occurrence of either would signify that the pathway is operational and the absence of both would indicate that it is inoperative or has been disabled.

## 27. Unconventional Alzheimer’s Disease: Elicitation of the Neuronal ISR by Stressors Distinct from AβPP-Derived *i*Aβ

The preceding sections analyzed the applications of the ISR-suppressing and BACE-activating therapies in the prevention and treatment of conventional AD. The present and following sections address the utilization of these approaches in unconventional Alzheimer’s disease. In both conventional and unconventional categories of AD, the disease is driven by *i*Aβ generated in the AβPP-independent pathway [8]. What distinguishes these two categories is only the causation of the disease, i.e., the manner of the activation of the AβPP-independent *i*Aβ generation pathway [8]. Conventionally, Alzheimer’s disease is caused by AβPP-derived *i*Aβ accumulated physiologically over the neuronal ISR-triggering T1 threshold. At these levels, AβPP-derived *i*Aβ mediates the activation of the PKR and/or HRI eIF2α kinases, eIF2α is phosphorylated at its Ser51, and the neuronal integrated stress response is elicited. Under the ISR conditions, cellular transcription and translation are radically re-programmed. Total protein synthesis is severely suppressed, whereas the production of a small subset of cellular proteins is activated. Among those, apparently, are the components essential for the activity of the AβPP-independent *i*Aβ generation pathway. When these components become available, the pathway is rendered operational and AD ensues.

It follows that the pivotal regulatory event in the development of AD is the elicitation of the neuronal integrated stress response. If this is indeed correct, and apparently it is, the essence of AD (in either of its forms) can be summarized thus: elicit the ISR in the neuronal cells, and the disease will follow. It is only logical to specify it even more precisely: elicit the neuronal integrated stress response IN ANY WAY and AD will follow. Accordingly, unconventional AD is defined as the disease triggered by the neuronal ISR elicited by stressors distinct from AβPP-derived *i*Aβ [8]. Conditions that can trigger unconventional AD via the elicitation of the neuronal ISR are both various and numerous. They include mechanical causes such as traumatic brain injury [206,207,208] and chronic traumatic encephalopathy [209], as well as bacterial and viral infections [210,211,212,213,214,215,216,217,218]. In a sufferer of viral encephalitis, for example, the probability of developing AD increases over thirty times [210,211,212,213,214] and a ten-fold increase in the occurrence of AD is seen in certain chronic bacterial infections [215,216,217,218]. Neuroinflammation, rheumatoid arthritis, osteoarthritis, and systemic inflammation in general are all strongly associated with AD [219,220,221,222,223,224,225,226,227,228,229,230]. Dementia associated with these conditions is often referred to as ADLD (AD-like dementia) or ADRD (AD-related dementia). In the framework of the ACH2.0, however, both types belong firmly in the category of unconventional AD [8].

**Figure 14:**
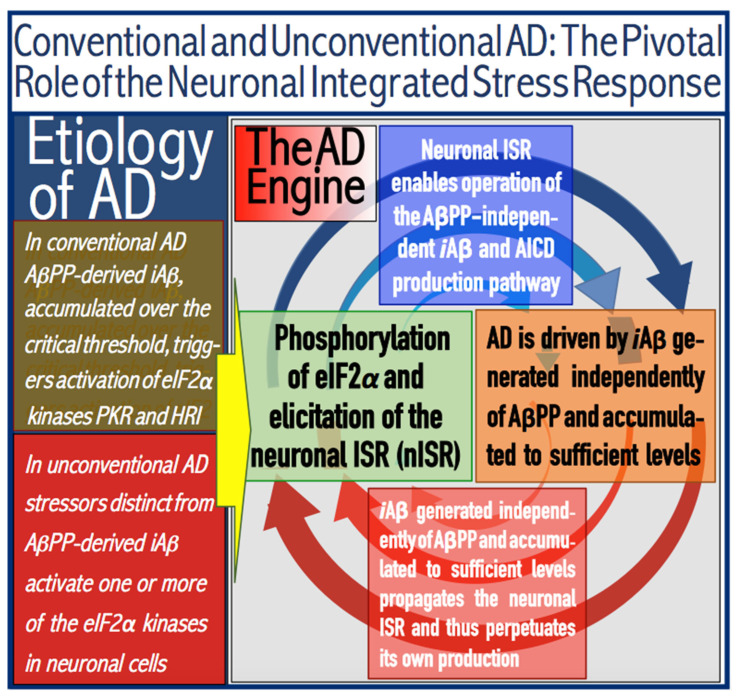
**Unconventional AD is caused by the neuronal ISR elicited by stressors distinct from AβPP-derived iAβ.** intraneuronal Aβ. *eIF2α:* eukaryotic translation initiation factor 2 alpha. *PKR and HRI:* eIF2α–specific kinases. *nISR:* neuronal integrated stress response. It is caused by phosphorylation of eIF2α at its Ser51 and manifests in radical transcriptional and translational reprogramming; it provides “missing” components of the AβPP-independent *i*Aβ generation pathway thus activating it. *AICD:* AβPP intracellular domain; one of the products of the gamma-cleavage. *The AD Engine*: escalating cycles of self-stimulated production of *i*Aβ in the AβPP-independent pathway (symbolized by arched red and blue arrows). In conventional AD, the activation of the PKR and HRI kinases in neuronal cells is triggered by AβPP-derived *i*Aβ accumulated over the T1 threshold “conventional stressor”). In unconventional AD, the activation of the eIF2α kinases in the neurons is accomplished by stressors distinct from AβPP-derived *i*Aβ (“unconventional stressors”). Diverse pathways leading to conventional and unconventional forms of AD conflate at the stage of the phosphorylation of eIF2α. The ensuing elicitation of the neuronal ISR is the pivotal point and realizes the uniform function in both conventional and unconventional forms of AD: it supplies the essential components of the AβPP-independent *i*Aβ generation pathway and thus enables its operation. *i*Aβ produced in this pathway fulfills two functions. One, it drives AD pathology and, two, it sustains the activity of PKR and HRI, maintains eIF2α in the phosphorylated state, propagates the ISR conditions, and thus perpetuates its own production in the AβPP-independent pathway, thus propelling the progression of AD. Note that the activation of the AβPP-independent *i*Aβ generation pathway equates with the commencement of AD in conventional but not unconventional cases of the disease. AD (both conventional and unconventional) commences when the AβPP-independent *i*Aβ production pathway becomes self-sustainable, i.e., the levels of *i*Aβ are above the T1 threshold. In conventional AD, this self-sustainability is attained from the instance of the activation of the pathway, which is triggered by the over-T1 levels of AβPP-derived *i*Aβ. In unconventional AD, the AβPP-independent *i*Aβ generation pathway is activated below the T1 threshold and the disease is triggered only when its *i*Aβ product accumulates to sufficient (i.e., above T1) levels. Hence, “…and accumulated to sufficient levels…” stated in the figure in relation to *i*Aβ generated independently of AβPP is relevant to the cases of unconventional AD only; in conventional AD, the AβPP-independent production of *i*Aβ (and the disease) initiates when the levels of AβPP-derived *i*Aβ are already sufficiently, over-T1, high (see Figure 3).

The ways in which the above conditions transmit their signals and cause the elicitation of the neuronal integrated stress response (thus causing unconventional AD) are likely as various as the conditions that produce them. There are, however, at least two prime candidates apparently common to all of these conditions. One is the impairment of the blood–brain barrier (BBB) shown to be compromised in all conditions listed above [231]. A compromised BBB can facilitate neuronal penetration by various stressors capable of eliciting the ISR. Another prime candidate is the reduction of cerebral blood flow, CBF, considered for a long time as a potential cause of AD [232,233,234,235,236,237,238]. The reduction of CBF can potentially act in many different ways in the elicitation of the neuronal ISR. There is, however, one obvious way described in the preceding sections, namely the activation of the HRI kinase. Indeed, the reduction of CBF was shown to cause mitochondrial dysfunction in neurons [239,240]. This leads, via the activation of OMA1, cleavage of DELE1, and exportation of one of DELE1 fragments to the cytosol, to the activation of HRI [94,95], phosphorylation of eIF2α, and elicitation of the neuronal ISR. Interestingly, it was suggested [8] that conventional AD could self-transform into unconventional AD by compromising the BBB and reducing CBF via, for example, the deposition of extracellular Aβ around cerebral blood vessels. This necessitates considering every case of symptomatic AD (for the purposes of its treatment) as, potentially, the unconventional disease. It should be emphasized that from the instance of the activation of the neuronal integrated stress response, conventional and unconventional AD are mechanistically identical; both are propelled by *i*Aβ generated independently of AβPP, which not only drives AD pathology but, following the T1 crossing, also propagates neuronal ISR conditions and thus perpetuates its own production. The concept of unconventional AD where the ISR elicited by stressors distinct from AβPP-derived *i*Aβ enables the operation of the self-sustainable AβPP-independent *i*Aβ generation pathway, and the comparative etiological relationship between conventional and unconventional forms of the disease, are illustrated in Figure 14.

## 28. Dynamics of *i*Aβ Accumulation in Unconventional AD

### 28.1. Effect of Long-Term Unconventional Activation of the AβPP-Independent iAβ Generation Pathway

**Figure 15:**
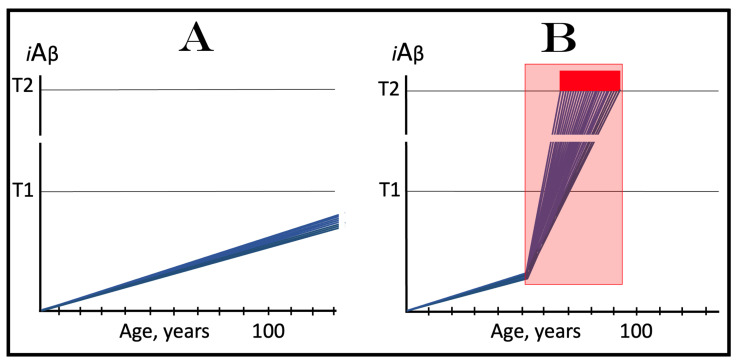
**Dynamics of iAβ accumulation in unconventional AD: Effect of long-term unconventional activation of the AβPP-independent *i*Aβ generation pathway**. *i*Aβ: intraneuronal Aβ. ***T*1** *threshold*: levels of *i*Aβ that trigger the activation of the eIF2α kinases PKR and/or HRI, elicitation of the neuronal ISR and initiation of the AβPP-independent production of *i*Aβ. ***T*2** *threshold*: levels of *i*Aβ, produced mainly in the AβPP-independent pathway, which trigger neuronal death. *Blue lines*: levels of *i*Aβ in individual neurons of a person. *Red Box*: apoptotic zone; the fraction of neurons that have committed apoptosis (or necroptosis) or are dead. *Pink Box*: duration of the occurrence of unconventional stressors capable of eliciting the neuronal ISR. *Panel* (**A**): Dynamics of accumulation of AβPP-derived *i*Aβ in a healthy individual. *i*Aβ would not reach the T1 threshold and AD would not occur unless the neuronal ISR is elicited by an unconventional stressor and, consequently, the AβPP-independent *i*Aβ production pathway is activated unconventionally. *Panel* (**B**): An event occurs or a condition develops that cause the long-term occurrence of unconventional stressors. The neuronal ISR is elicited and the AβPP-independent *i*Aβ generation pathway is activated unconventionally and is sustained by a life-long presence of unconventional stressors. *i*Aβ produced in this pathway rapidly accumulates, crosses the T1 threshold, renders the pathway self-sustainable, and unconventional AD ensues.

The dynamics of *i*Aβ accumulation in unconventional AD differs significantly from that in the conventional disease. In the latter, the slow phase of AβPP-derived *i*Aβ accumulation in individual neurons morphs into the phase of rapid *i*Aβ accumulation due to the activation of the AβPP-independent *i*Aβ generation pathway following the crossing of the T1 threshold. In the former, the elicitation of the neuronal ISR by unconventional stressors and, consequently, the unconventional activation of the AβPP-independent *i*Aβ production pathway and switch to the rapid *i*Aβ accumulation phase in the individual neurons occur always at the levels of AβPP-derived *i*Aβ below the T1 threshold (otherwise, the conventional AD would be triggered by the T1 crossing and the occurrence of the unconventional stressor at this stage would be inconsequential). This dynamic is illustrated in Figure 15. Panel A of Figure 15 depicts the accumulation of *i*Aβ in individual neurons of a healthy person. *i*Aβ is derived only by AβPP proteolysis and does not cross the T1 threshold within the lifetime of the individual. In panel B of Figure 15, an event occurs or a condition develops that cause the elicitation of the neuronal integrated stress response by stressor(s) distinct from AβPP-derived *i*Aβ; in the depicted scenario (defined below as “the worst case scenario”), this unconventional stressor(s) is present through the remaining portion of the lifetime. This triggers the unconventional activation of the AβPP-independent *i*Aβ production pathway and the switch to the rapid phase of *i*Aβ accumulation. When the levels of *i*Aβ reach and cross the T1 threshold, the AβPP-independent *i*Aβ generation pathway becomes self-sustainable (i.e., sustained by the *i*Aβ product of this pathway, see Section 9 above) and AD commences.

### 28.2. Effect of Transient Unconventional Activation of the AβPP-Independent iAβ Generation Pathway

**Figure 16:**
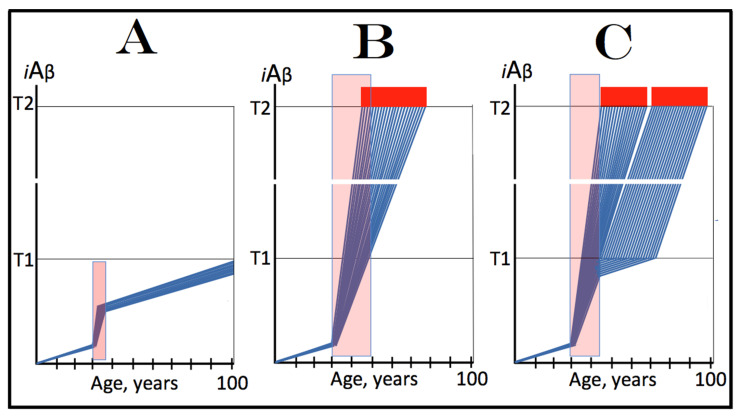
**Dynamics of iAβ accumulation in unconventional AD: effect of transient unconventional activation of the AβPP-independent *i*Aβ generation pathway**. *i*Aβ: intraneuronal Aβ. ***T*1** *threshold*: levels of *i*Aβ that trigger the activation of the eIF2α kinases PKR and/or HRI, and elicitation of the neuronal ISR. ***T*2** *threshold*: levels of *i*Aβ which trigger neuronal death. *Blue lines*: levels of *i*Aβ in individual neurons. *Red Box*: apoptotic zone; the fraction of neurons that have committed apoptosis or are dead. *Pink Boxes*: duration of the occurrence of unconventional stressors capable of eliciting the neuronal ISR and of unconventionally activating the AβPP-independent *i*Aβ production pathway. *Panel* (**A**): The duration of the occurrence of unconventional stressors is insufficient to cause the T1 crossing within the lifetime of the individual. Unconventional stressors elicit the neuronal ISR, unconventionally activate the AβPP-independent *i*Aβ generation pathway, and its *i*Aβ product rapidly accumulates. When unconventional stressors are no longer present, operation of the AβPP-independent *i*Aβ production pathway ceases. AβPP-derived *i*Aβ resumes its accumulation at a pre-unconventional stressors occurrence rate. It does not reach the T1 threshold and no AD occurs within the lifetime of the individual. *Panel* (**B**): The duration of the occurrence of unconventional stressors is sufficient for *i*Aβ produced in the unconventionally activated AβPP-independent *i*Aβ generation pathway to cross the T1 threshold in all neurons. Following the T1 crossing, this pathway becomes self-sustainable. The withdrawal of unconventional stressors at this stage would affect neither the operation of the AβPP-independent *i*Aβ production pathway nor the progression of AD. *Panel* (**C**): At the time of withdrawal of unconventional stressors, *i*Aβ crossed the T1 in only a fraction of the neurons where the AβPP-independent *i*Aβ production pathway remains operational and the AD pathology progresses unimpeded. In sub-T1 neurons, the AβPP-independent *i*Aβ production pathway is rendered inoperative but the accumulation of AβPP-derived *i*Aβ continues. When it crosses the T1 threshold, the AβPP-independent *i*Aβ production pathway is activated and the progression of the AD pathology commences.

Unconventional activation of the AβPP-independent *i*Aβ generation pathway, occurring prior to the crossing of the T1 threshold by *i*Aβ, could be transient. In such a case, after certain duration an unconventional stressor is removed and can no longer sustain the ISR conditions and support the activity of the AβPP-independent *i*Aβ production pathway. The outcome of such transient unconventional activation of the AβPP-independent *i*Aβ generation pathway would depend on the extent of accumulation of *i*Aβ produced in this pathway prior to the withdrawal of the unconventional stressor. Figure 16 illustrates three potential outcomes. The first two are rather trivial. In panel A of Figure 16, the duration of the unconventional activity of AβPP-independent *i*Aβ generation pathway is short and the extent of accumulation of *i*Aβ produced in this pathway is relatively small and inconsequential (except it can enable or hasten the crossing of the T^0^ threshold and the occurrence of AACD). When the unconventional stressor is withdrawn, the accumulation of AβPP-derived *i*Aβ resumes at a slow rate. It would not reach the T1 threshold within the lifetime of the individual and no AD would occur. In panel B of Figure 16, the duration of the presence of unconventional stressors is sufficient for *i*Aβ produced in the unconventionally activated AβPP-independent *i*Aβ generation pathway to cross the T1 threshold and for the pathway to become self-sustainable in all affected neurons. The withdrawal of the unconventional stressor at this point would not affect the continuous operation of the AβPP-independent *i*Aβ generation pathway (sustained now by over-T1 levels of *i*Aβ) and the progression of AD. In panel C of Figure 16, by the time of withdrawal of unconventional stressors, *i*Aβ reached the T1 threshold only in a fraction of the affected neurons. In these (over-T1) neurons, the AβPP-independent *i*Aβ generation pathway is self-sustainable and remains operational. In sub-T1 neurons, the withdrawal of unconventional stressors renders the AβPP-independent *i*Aβ production pathway inoperative. However, the accumulation of *i*Aβ continues, and upon crossing of the T1 threshold the AβPP-independent *i*Aβ generation pathway is conventionally activated and cellular AD pathology commences. In these individuals, the progression of AD is slow but inevitable.

**Figure 17:**
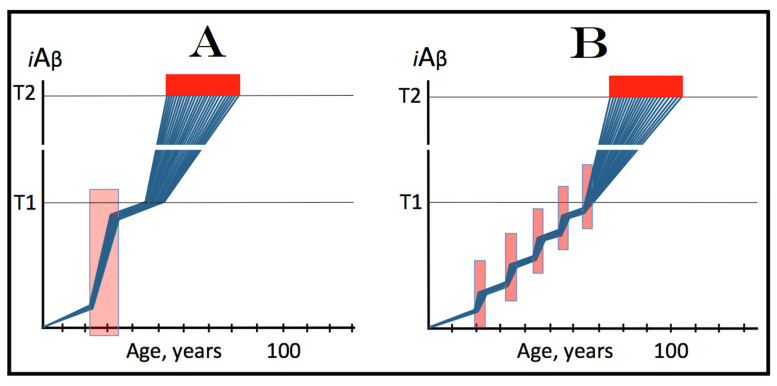
**Transient unconventional activation of the AβPP-independent *i*Aβ generation pathway may cause unconventional AD in a delayed manner**. *i*Aβ: intraneuronal Aβ. ***T*1** *threshold*: levels of *i*Aβ that trigger the activation of the eIF2α kinases PKR and/or HRI, elicitation of the neuronal ISR, and initiation of the AβPP-independent production of *i*Aβ. ***T*2** *threshold*: levels of *i*Aβ, produced mainly in the AβPP-independent pathway, which trigger neuronal death. *Blue lines*: levels of *i*Aβ in individual neurons of a person. *Red Box*: apoptotic zone; the fraction of neurons that have committed apoptosis (or necroptosis) or are dead. *Pink Boxes*: duration of the occurrence of unconventional stressors capable of eliciting the neuronal ISR and, consequently, of unconventionally activating the AβPP-independent *i*Aβ production pathway. *Panel* (**A**): The delayed effect of a single transient unconventional activation of the AβPP-independent *i*Aβ production pathway. The transient occurrence of unconventional stressors elicits the neuronal ISR and unconventionally activates the AβPP-independent *i*Aβ generation pathway. Its operation ceases when stressors are no longer present; its transiently produced *i*Aβ product has accumulated to a substantial extent but its levels are still below the T1 threshold. The accumulation of AβPP-derived *i*Aβ resumes at a slow, pre-unconventional stressors occurrence rate but from a significantly elevated baseline. Consequently, the T1 crossing occurs much sooner than it would in the absence of the unconventional activity of the AβPP-independent *i*Aβ generation pathway. At this point, the pathway is re-activated and sustained conventionally by over-T1 levels of *i*Aβ, and AD ensues. *Panel* (**B**): The delayed effect of the recurrent rounds of the unconventional transient activity of the AβPP-independent *i*Aβ generation pathway. The transient unconventionally activated operation of the AβPP-independent *i*Aβ generation pathway occurs recurrently. Each round results in a pulse of rapid accumulation of *i*Aβ and after each round the slow accumulation of AβPP-derived *i*Aβ resumes from an elevated baseline, i.e., in an incremental stepwise manner, until the T1 threshold is crossed, the activity of the AβPP-independent *i*Aβ production pathway becomes self-sustainable, and AD commences. In both scenarios, the transient event/events cause AD not immediately but after a considerable delay required for the accumulation of AβPP-derived *i*Aβ from the elevated baselines to the T1 threshold at a slow pre-*i*Aβ baseline-elevating event rate (without *i*Aβ baseline-elevating events, the T1 crossing would have occurred much later or not at all).

Figure 17 illustrates two additional scenarios, where unconventional stressors are withdrawn prior to the crossing of the T1 threshold, that are more complex. In panel A of Figure 17, the extent of the accumulation of *i*Aβ produced during the transient unconventional operation of the AβPP-independent *i*Aβ generation pathway is significant. Following the withdrawal of the unconventional stressor, the accumulation of AβPP-derived *i*Aβ resumes from a substantially elevated but still sub-T1 baseline. Due to the proximity of the T1 threshold, its crossing by AβPP-derived *i*Aβ would occur much sooner than in the absence of the *i*Aβ baseline-elevating event. The AβPP-independent *i*Aβ generation pathway would be re-activated, now in a conventional manner (via the neuronal ISR elicited by over-T1 levels of *i*Aβ), and AD would commence. In this scenario, the occurrence of AD is enabled by the transient unconventional activity of the AβPP-independent *i*Aβ generation pathway, and this type of the disease can be considered, therefore, unconventional. It is best exemplified by AD occurring in sufferers of a traumatic brain injury not immediately following the event but a decade or two after the event (the lag period is required for the rapid accrual of *i*Aβ produced independently of AβPP followed by the accumulation of AβPP-derived *i*Aβ from the elevated baseline to the T1 threshold; in the absence of the *i*Aβ baseline-elevating event, the T1 crossing would have occurred much later or not at all). 

The scenario illustrated in panel B of Figure 17 is conceptually similar but rapid elevation of the levels of *i*Aβ occurs in an incremental stepwise manner and results from the recurrent rounds of the unconventional transient elicitation of the neuronal ISR and, consequently, of the unconventional transient activity of the AβPP-independent *i*Aβ generation pathway. After each round of the rapid accumulation of *i*Aβ produced independently of AβPP, the slow accumulation of AβPP-derived *i*Aβ resumes from an elevated baseline until the T1 threshold is crossed, the activity of the AβPP-independent *i*Aβ production pathway becomes self-sustainable, and AD commences. In this scenario, as in the preceding one, the occurrence of AD is enabled by a series of unconventional transient activations of the AβPP-independent *i*Aβ generation pathway. Therefore, this type of the disease can also be considered unconventional. It is best exemplified by traumatic chronic encephalopathy, and can be caused by the recurrent rounds of systemic inflammation or viral/bacterial infection.

## 29. Inhibition of the Neuronal Integrated Stress Response in the Prevention and Treatment of Unconventional AD

The present and the following sections address therapeutic strategies for unconventional AD. In the light of the preceding considerations and for the purposes of brevity, only the “worst case scenario” of unconventional AD will be discussed below. It entails the continuous presence (from the time of its appearance for the remaining lifetime) of an unconventional stressor(s) capable of eliciting the neuronal integrated stress response. Consequently, the AβPP-independent *i*Aβ generation pathway would operate uninterruptedly from the instance of its unconventional activation at the low levels of AβPP-derived *i*Aβ for the remaining portion of the lifespan. Potential outcomes of alternative scenarios are reviewed in Section 36 below.

### 29.1. Long-Term Inhibition of the Neuronal ISR in the Prevention and Treatment of Unconventional AD

**Figure 18:**
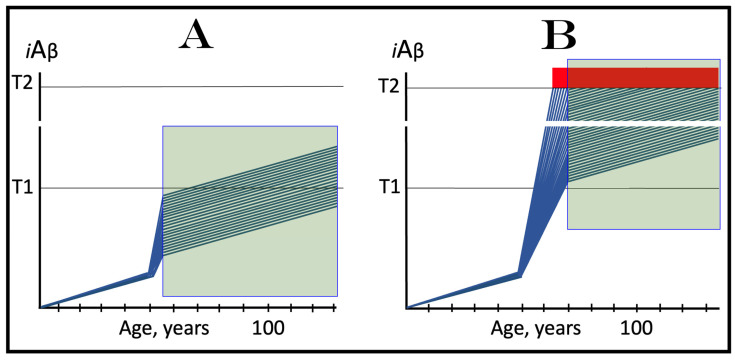
**Effect of the long-term inhibition of the neuronal ISR in the prevention and treatment of unconventional AD.** *i*Aβ: intraneuronal Aβ. ***T*1** *threshold*: levels of *i*Aβ that trigger the activation of the eIF2α kinases PKR and/or HRI, elicitation of the neuronal ISR, and initiation of the AβPP-independent production of *i*Aβ. ***T*2** *threshold*: levels of *i*Aβ, produced mainly in the AβPP-independent pathway, which trigger neuronal death. *Blue lines*: levels of *i*Aβ in individual neurons of a person. *Red Box*: apoptotic zone; the fraction of neurons that have committed apoptosis (or necroptosis) or are dead. *Green Boxes*: the duration of the presence of an inhibitor of the IRS. *Panel* (**A**): The long-term treatment with the ISR-inhibiting drug is commenced prior to the T1 crossing. By this time, the neuronal ISR has been elicited by unconventional stressors and the AβPP-independent *i*Aβ generation pathway has been unconventionally activated; its *i*Aβ product has rapidly accumulated but is still below the T1 threshold. Following the drug’s administration, the neuronal ISR is suppressed and, consequently, the AβPP-independent *i*Aβ production pathway is disabled for the duration of the treatment. The accumulation of AβPP-derived *i*Aβ continues but at a reduced rate. Eventually, it crosses the T1 threshold, but the AβPP-independent *i*Aβ generation pathway remains inoperative. AβPP-derived *i*Aβ is unlikely to reach AD pathology-causing levels and AD symptoms are unlikely to occur for the duration of the treatment. *Panel* (**B**): Effect of long-term ISR suppression in the treatment of unconventional AD. By the time of the implementation of the ISR suppression therapy, the neuronal ISR has been elicited by unconventional stressors and the AβPP-independent *i*Aβ generation pathway has been unconventionally activated. Its *i*Aβ product has rapidly accumulated and crossed the T1 threshold. The pathway was rendered self-sustainable and AD commenced. The rapid accumulation of *i*Aβ has continued, reached AD pathology-causing levels, and crossed the T2 threshold in a fraction of the neurons, and AD symptoms have manifested. The suppression of the ISR disables the AβPP-independent *i*Aβ generation pathway. However, the levels of *i*Aβ remain high and its accumulation continues via the influx of AβPP-derived *i*Aβ. The crossings of the T2 threshold and the progression of AD persist at a reduced rate for the duration of the treatment.

The effects of the long-term inhibition of the neuronal integrated stress response in the prevention and treatment of unconventional Alzheimer’s disease are illustrated in Figure 18. Panel A of Figure 18 depicts the effect of an ISR inhibitor in the prevention of unconventional AD. By the commencement of the drug’s administration (green box), the neuronal ISR has been elicited by unconventional stressor(s) and the AβPP-independent *i*Aβ generation pathway has been unconventionally activated at the levels of AβPP-derived *i*Aβ below the T1 threshold. *i*Aβ produced in this pathway has been rapidly accumulating but is still below the T1 threshold. The administration of the drug suppresses the integrated stress response. The components essential for the operation of the AβPP-independent *i*Aβ generation pathway are no longer supplied and in their absence the pathway is rendered inoperative. The accumulation of *i*Aβ continues but at a slow rate and supported only by the AβPP proteolysis. Eventually, the levels of *i*Aβ cross the T1 threshold but this event is inconsequential since the ISR cannot be elicited and the AβPP-independent *i*Aβ generation pathway cannot be re-activated in the presence of the drug. The accumulation of AβPP-derived *i*Aβ proceeds at a slow rate for the duration of the treatment. AD pathology-causing *i*Aβ levels would not be reached for the remaining lifetime of the treated individual.

Panel B of Figure 18 shows the effect of an ISR inhibitor in the treatment of unconventional AD. By the time of the administration (green box) of the drug, the AβPP-independent *i*Aβ generation pathway has been unconventionally activated at the levels of AβPP-derived *i*Aβ below the T1 threshold. *i*Aβ produced in this pathway has rapidly accumulated, crossed the T1 threshold, and the pathway became self-sustainable. *i*Aβ continued its rapid accumulation, reached AD pathology-causing levels, crossed the T2 threshold in a fraction of the neurons, and AD symptoms have manifested. Suppression of the neuronal ISR by the drug at this stage discontinues the supply of the essential components of the AβPP-independent *i*Aβ generation pathway and disables its operation. The levels of *i*Aβ, however, remain in the AD-pathology-causing range. Moreover, the accumulation of AβPP-derived *i*Aβ continues and the T2 threshold is being crossed; the progression of AD has not been stopped, it continues albeit at a reduced rate.

### 29.2. Transient Inhibition of the Neuronal ISR in the Prevention and Treatment of Unconventional AD

**Figure 19:**
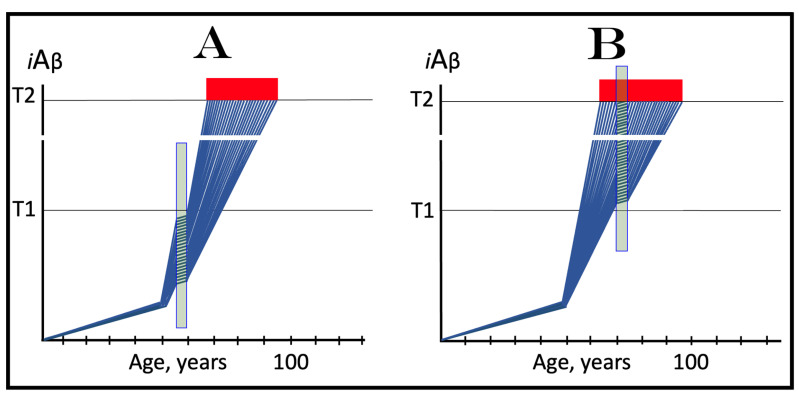
**Effect of the transient inhibition of the neuronal ISR in the prevention and treatment of unconventional AD.** *i*Aβ: intraneuronal Aβ. ***T*1** *threshold*: levels of iAβ that trigger the activation of the eIF2α kinases PKR and/or HRI, elicitation of the neuronal ISR, and initiation of the AβPP-independent production of *i*Aβ. ***T*2** *threshold*: levels of *i*Aβ, produced mainly in the AβPP-independent pathway, which trigger neuronal death. *Blue lines*: levels of *i*Aβ in individual neurons of a person. *Red Box*: apoptotic zone; the fraction of neurons that have committed apoptosis (or necroptosis) or are dead. *Green Boxes*: the duration of the presence of an inhibitor of the IRS. *Panel* (**A**): The transient treatment with the ISR-inhibiting drug is administered prior to the T1 crossing. By this time, the neuronal ISR has been elicited by unconventional stressors and the AβPP-independent *i*Aβ generation pathway has been unconventionally activated; its *i*Aβ product has rapidly accumulated but is still below the T1 threshold. Following the drug’s administration, the neuronal ISR is suppressed and, consequently, the AβPP-independent *i*Aβ production pathway is disabled for the duration of the treatment. The accumulation of AβPP-derived *i*Aβ continues but at a slow rate. When the drug is withdrawn, the neuronal ISR is re-elicited by unconventional stressors, the AβPP-independent *i*Aβ generation pathway is unconventionally re-activated, and the rapid accumulation of iAβ resumes. The T1 crossing renders the AβPP-independent *i*Aβ production pathway self-sustainable, AD commences (delayed by the duration of the treatment) and progresses uninterrupted. *Panel* (**B**): Effect of the transient ISR suppression on treatment of unconventional AD. By the time of the implementation of the ISR suppression therapy, the neuronal ISR has been elicited by unconventional stressors and the AβPP-independent *i*Aβ generation pathway has been unconventionally activated. Its *i*Aβ product has rapidly accumulated and crossed the T1 threshold. The pathway was rendered self-sustainable and AD commenced. The rapid accumulation of *i*Aβ has continued, it reached AD pathology-causing levels and crossed the T2 threshold in a fraction of the neurons, and AD symptoms have manifested. The suppression of the ISR disables the AβPP-independent *i*Aβ generation pathway. However, AβPP-derived *i*Aβ continues to accumulate and the disease progresses, albeit at a reduced rate. Following the withdrawal of the drug, the accumulation of *i*Aβ and the progression of AD resume at the pre-treatment rate. The transient ISR suppression treatment drug would provide only transient reprieve of slow progression of the disease lasting no more than the duration of the treatment.

Thus, long-term suppression of the neuronal ISR could potentially be beneficial in both, the prevention and treatment of unconventional AD. However, for the reasons discussed in Section 11 above, the long-term administration of inhibitors of the integrated stress response appears unfeasible. On the other hand, as was mentioned above, the transient utilization of an ISR inhibitor caused no noticeable adverse effects in experimental mouse models [125]. It is of interest, therefore, to consider the possible effect of the transient administration of ISR inhibitors in the prevention and treatment of unconventional AD (Figure 19). As discussed above, the transient utilization of ISR inhibitors for the prevention of conventional AD serves no purpose whatsoever because the ISR has not yet been elicited at this stage (see Section 12 above). This is not the case with unconventional AD. In panel A of Figure 19, the initial state of the levels of *i*Aβ in individual neurons at the commencement of the treatment is the same as in panel A of the preceding figure. The AβPP-independent *i*Aβ generation pathway has been unconventionally activated but the levels of *i*Aβ are still below the T1 threshold. The transient administration of the drug (green box) would suppress the neuronal ISR and thus disable the AβPP-independent *i*Aβ generation pathway. The accumulation of *i*Aβ would continue at a reduced rate, supported only by AβPP proteolysis. Following the withdrawal of the drug, with the unconventional stressor present, the ISR would be re-elicited and the AβPP-independent *i*Aβ production pathway re-activated, rendered self-sustainable upon the T1 crossing, and AD would commence. The transient suppression of the ISR would, thus, delay the occurrence of the disease by no more than the duration of the treatment.

In panel B of Figure 19, the initial state of the levels of *i*Aβ in the individual neurons at the commencement of the treatment is also the same as in panel B of the preceding figure. The transient administration of the drug (green box) at this stage would suppress the neuronal integrated stress response and render the AβPP-independent *i*Aβ generation pathway inoperative. However, the influx of AβPP-derived *i*Aβ would continue at a slow rate, and likewise so would the progression of the disease. When the drug is withdrawn, the accumulation of *i*Aβ and the progression of AD would resume at the pre-treatment rate. Thus, the transient administration of the ISR-suppressing drug would provide only a transient reprieve of slow progression of the disease lasting no more (actually less because of the continuous accumulation of *i*Aβ during the treatment period) than the duration of the treatment.

## 30. Effect of the Long-Term Targeted Degradation of *i*Aβ via the Activation of BACE1 and/or BACE2 in the Prevention of Unconventional AD

**Figure 20:**
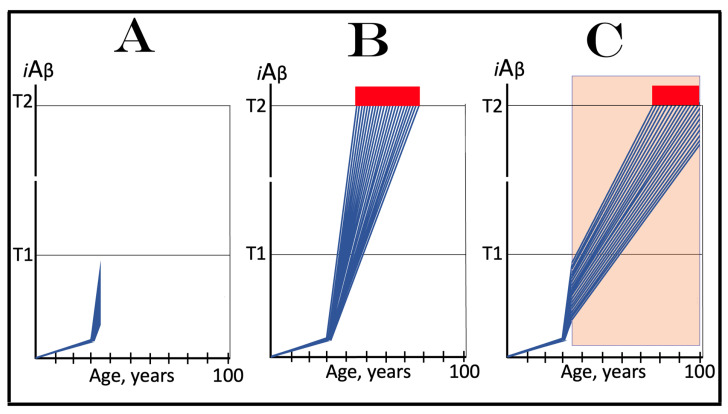
**Effect of the long-term targeted degradation of *i*Aβ via the activation of BACE1 and/or BACE2 in the prevention of unconventional AD**. *i*Aβ: intraneuronal Aβ. ***T*1** *threshold*: levels of *i*Aβ that trigger the activation of the eIF2α kinases PKR and/or HRI, elicitation of the neuronal ISR, and initiation of the AβPP-independent production of *i*Aβ. ***T*2** *threshold*: levels of *i*Aβ, produced mainly in the AβPP-independent pathway, which trigger neuronal death. *Blue lines*: levels of *i*Aβ in individual neurons of a person. *Red Box*: apoptotic zone; the fraction of neurons that have committed apoptosis (or necroptosis) or are dead. *Orange Box*: the duration of the presence of activators of BACE1 and/or BACE2. *Panel* (**A**): The initial state of the levels of AβPP-derived *i*Aβ in individual neurons of a person. The ISR has been elicited by an unconventional stressor at low levels of AβPP-derived *i*Aβ. The AβPP-independent *i*Aβ generation pathway has been unconventionally activated and its *i*Aβ product has rapidly accumulated but is still below the T1 threshold. *Panel* (**B**): The evolution of the initial state in the untreated individual. *i*Aβ continues its rapid accumulation and crosses the T1 threshold in all affected neurons. The AβPP-independent *i*Aβ generation pathway becomes self-sustainable and AD commences and progresses unimpeded. *Panel* (**C**): The evolution of the initial state in the presence of a BACE1- and/or BACE2-activating, *i*Aβ-degrading drug. The rate of the influx of *i*Aβ produced in the AβPP-independent pathway exceeds the rate of its degradation by the drug. The accumulation of *i*Aβ continues at a reduced rate but from elevated baselines for the duration of the treatment. The T1 threshold is eventually crossed, and the AβPP-independent *i*Aβ generation pathway is rendered self-sustainable. AD commences but its progress is slower than in the absence of the *i*Aβ-depleting drug. The drug delays the commencement of the disease and slows down its progression when it occurs.

The analysis presented in Section 18 above has indicated that *i*Aβ depletion via the activation of BACE1 and/or BACE2 would be highly effective in the prevention of conventional AD. Moreover, it suggested that once-in-a-lifetime-only transient administration of the *i*Aβ depletion therapy could be sufficient to protect from both conventional AD and AACD for the remaining lifetime of an individual. This, however, is not the case in the prevention of unconventional AD. The effect of the long-term *i*Aβ degradation therapy in the prevention of unconventional AD is illustrated in Figure 20. Panel A of Figure 20 depicts the initial state of the levels of *i*Aβ in the individual neurons of a person. At this point, the ISR has already been elicited by an unconventional stressor at low levels of AβPP-derived *i*Aβ. The AβPP-independent *i*Aβ generation pathway has been unconventionally activated and its *i*Aβ product has rapidly accumulated but is still below the T1 threshold. Panel B of Figure 20 shows the evolution of the initial state in the untreated individual. *i*Aβ continues its rapid accumulation and crosses the T1 threshold in all affected neurons. The AβPP-independent *i*Aβ generation pathway becomes self-sustainable, and AD commences and rapidly progresses. 

Panel C of Figure 20 illustrates the evolution of the initial state in the presence of an *i*Aβ-degrading drug (orange box). As argued in Section 23 above, the rate of the targeted degradation of *i*Aβ mediated by the drug cannot match the rate of the influx of *i*Aβ produced in the AβPP-independent pathway. The accumulation of *i*Aβ continues in the presence of the drug, albeit at a reduced rate, but from a relatively high baseline. The T1 threshold is eventually crossed, and the AβPP-independent *i*Aβ generation pathway is rendered self-sustainable. AD commences but its progress is slower than in the absence of the *i*Aβ-depleting drug. The drug, therefore, delays the commencement of the disease and slows down its progression when it occurs.

## 31. Effect of the Long-Term Targeted Degradation of iAβ via the Activation of BACE1 and/or BACE2 in the Treatment of Unconventional AD

**Figure 21:**
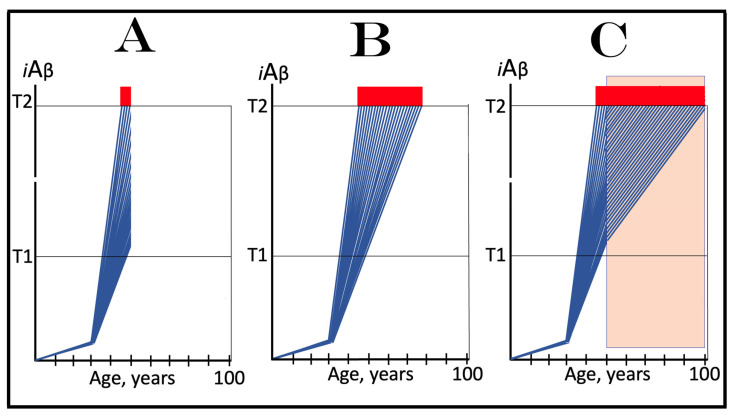
**Effect of the long-term targeted degradation of iAβ via the activation of BACE1 and/or BACE2 in the treatment of unconventional AD**. *i*Aβ: intraneuronal Aβ. ***T*1** *threshold*: levels of *i*Aβ that trigger the activation of the eIF2α kinases PKR and/or HRI, elicitation of the neuronal ISR, and initiation of the AβPP-independent production of *i*Aβ. ***T*2** *threshold*: levels of *i*Aβ, produced mainly in the AβPP-independent pathway, which trigger neuronal death. *Blue lines*: levels of *i*Aβ in individual neurons of a person. *Red Box*: apoptotic zone; the fraction of neurons that have committed apoptosis (or necroptosis) or are dead. *Orange Box*: the duration of the presence of activators of BACE1 and/or BACE2. *Panel* (**A**): The initial state of the levels of *i*Aβ in the individual neurons of an unconventional AD patient at the commencement of the *i*Aβ degradation therapy. The ISR has been elicited by an unconventional stressor and the AβPP-independent *i*Aβ generation pathway has been unconventionally activated. Its *i*Aβ product has rapidly accumulated and crossed the T1 threshold. The AβPP-independent *i*Aβ generation pathway was rendered self-sustainable and AD commenced. *i*Aβ continued its rapid accumulation, reached AD pathology-causing levels and crossed the T2 threshold in a fraction of the affected neurons, and AD symptoms manifested. *Panel* (**B**): The evolution of the initial state in the untreated patient. The rapid accumulation of *i*Aβ produced in the AβPP-independent pathway continues unimpeded, additional neurons cross the T2 threshold and commit apoptosis or necroptosis, and the disease enters its end stage. *Panel* (**C**): The evolution of the initial state in the presence of the BACE1- and/or BACE2-activating, *i*Aβ-degrading drug. The rate of the influx of *i*Aβ produced in the AβPP-independent pathway exceeds the rate of its degradation by the drug. The accumulation of *i*Aβ and the progression of the disease continue but at a reduced rate.

Effects of the targeted *i*Aβ degradation therapy in the prevention of conventional and unconventional AD are drastically different because the processes driving the accumulation of *i*Aβ prior to the crossing of the T1 threshold in these two types of the disease are also distinctly different. In conventional AD, sub-T1 *i*Aβ is produced solely in the AβPP proteolytic pathway whereas in unconventional AD it is generated also in the unconventionally activated AβPP-independent pathway. On the other hand, over-T1 *i*Aβ, which drives the disease, is generated mainly in the AβPP-independent pathway in both conventional and unconventional types of AD. Therefore, the effect of the targeted *i*Aβ degradation therapy in the treatment of unconventional AD, illustrated in Figure 21, is similar to that projected for the treatment of conventional AD (see Section 24 above). Panel A of Figure 21 shows the initial state of the levels of *i*Aβ in the individual neurons of an unconventional AD patient at the commencement of the *i*Aβ degradation therapy. The integrated stress response has been elicited by an unconventional stressor and the AβPP-independent *i*Aβ generation pathway has been activated unconventionally at the low levels of AβPP-derived *i*Aβ. *i*Aβ produced independently of AβPP has rapidly accumulated and crossed the T1 threshold. The AβPP-independent *i*Aβ generation pathway became self-sustainable and AD commenced. *i*Aβ continued its rapid accumulation, crossed the T2 threshold in a fraction of the affected neurons, and AD symptoms manifested. Panel B of Figure 21 depicts the evolution of the initial state in the untreated patient. The rapid accumulation of *i*Aβ produced in the AβPP-independent pathway continues unimpeded, additional neurons cross the T2 threshold and commit apoptosis or necroptosis, and the disease enters its end stage. Panel C of Figure 21 illustrates the evolution of the initial state in the presence of the BACE1- and/or BACE2-activating drug (orange box). The drug-mediated targeted degradation of *i*Aβ cannot match its influx in the AβPP-independent pathway; its accumulation continues, and the T2 threshold is being crossed by the additional neurons. The progression of the disease is not stopped; it persists, but at a reduced rate.

## 32. Effects of the Transient Activation of BACE1 and/or BACE2 in the Prevention and Treatment of Unconventional AD

**Figure 22:**
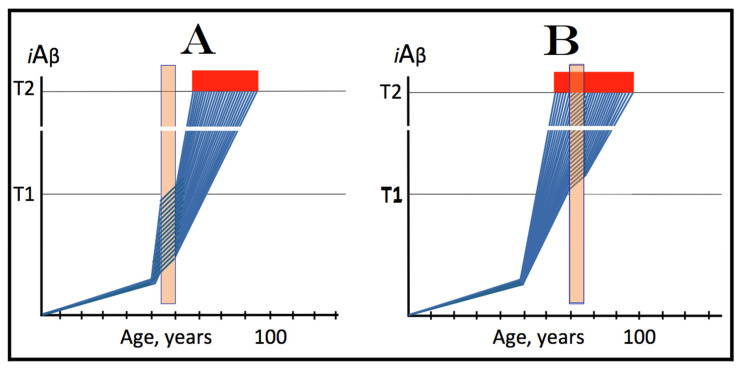
**Effects of the transient activation of BACE1 and/or BACE2 in the prevention and treatment of unconventional AD***. i*Aβ: intraneuronal Aβ. ***T*1** *threshold*: levels of iAβ that trigger the activation of the eIF2α kinases PKR and/or HRI, elicitation of the neuronal ISR, and initiation of the AβPP-independent production of *i*Aβ. ***T*2** *threshold*: levels of *i*Aβ, produced mainly in the AβPP-independent pathway, which trigger neuronal death. *Blue lines*: levels of *i*Aβ in individual neurons of a person. *Red Box*: apoptotic zone; the fraction of neurons that have committed apoptosis (or necroptosis) or are dead. *Orange Boxes*: the duration of the presence of activators of BACE1 and/or BACE2. *Panel* (**A**): The transient treatment with the BACE1- and/or BACE2-activating drug is administered prior to the T1 crossing. The neuronal ISR has already been elicited by an unconventional stressor, the AβPP-independent *i*Aβ generation pathway has been unconventionally activated, and its iAβ product has been rapidly accumulating but still below the T1 threshold at the commencement of treatment. The rate of degradation of *i*Aβ by activated BACE1 and/or BACE2 cannot match that of its influx in the AβPP-independent pathway, and its accumulation continues although at a reduced rate. When the drug is withdrawn, the accumulation of *i*Aβ resumes at the pre-treatment rate. It crosses the T1 threshold, the AβPP-independent *i*Aβ generation pathway is rendered self-sustainable, and AD commences. The effect of the preventive transient targeted *i*Aβ degradation therapy via the activation of BACE1 and/or BACE2 is only a delay in the occurrence of the disease, which is no longer than the duration of the treatment. *Panel* (**B**): Activator(s) of BACE1- and/or BACE2 are administered transiently to a symptomatic AD patient. Since the rate of the influx of *i*Aβ produced in the AβPP-independent pathway is greater than the rate of its efflux via degradation by activated BACE1 and/or BACE2, *i*Aβ accumulation and, consequently, the progression of AD in the presence of the drug continue, although at a reduced rate. When the drug is withdrawn, the *i*Aβ accumulation, the T2 crossings, and the progression of AD all resume at the pre-treatment rate. The transient *i*Aβ degradation therapy, therefore, would provide only a short reprieve of slow progression of the disease for no longer than the duration of the treatment.

The following sections below consider the effects of a composite therapy for unconventional AD, which includes transient *i*Aβ degradation treatment. For the purposes of comparison, the present section analyses the potential effects of the transient *i*Aβ degradation treatment alone. These effects are illustrated in Figure 22. In panel A of Figure 22, the transient *i*Aβ degradation therapy commences when the levels of *i*Aβ in the individual neurons of a person are below the T1 threshold. By this time, the neuronal integrated stress response has already been elicited by an unconventional stressor, the AβPP-independent *i*Aβ generation pathway has been unconventionally activated, and *i*Aβ produced in this pathway has been rapidly accumulating. The drug-mediated targeted degradation of *i*Aβ (orange box) cannot match its influx in the AβPP-independent pathway, and its accumulation continues although at a slower rate. When the drug is withdrawn, the accumulation of *i*Aβ resumes at the pre-treatment rate. It crosses the T1 threshold, the AβPP-independent *i*Aβ generation pathway is rendered self-sustainable, and AD commences. Thus, the effect of the preventive transient targeted *i*Aβ degradation therapy via the activation of BACE1 and/or BACE2 is only a delay in the crossing of the T1 threshold and, consequently, in the commencement of the disease; this delay is, in fact, shorter that the duration of the treatment (due to the continued accumulation of *i*Aβ during the treatment).

In panel B of Figure 22, AD symptoms have already manifested. By this stage, the neuronal integrated stress response has been activated by an unconventional stressor at the levels of AβPP-derived *i*Aβ below the T1 threshold. Consequently, the AβPP-independent *i*Aβ generation pathway has been unconventionally activated and the levels of *i*Aβ have rapidly increased. When they crossed the T1 threshold, the AβPP-independent *i*Aβ generation pathway became self-sustainable and AD commenced. *i*Aβ continued to rapidly accumulate and by the time of administration of the drug (orange box) has crossed the T2 threshold in a fraction of the affected neurons. Since the rate of the influx of *i*Aβ produced in the AβPP-independent pathway is greater than the rate of its efflux (i.e., its drug-mediated targeted degradation via the activation of BACE1 and/or BAE2), the accumulation of *i*Aβ and, consequently, the progression of AD in the presence of the drug continue, albeit at a slower rate than in the absence of the drug. When the drug is withdrawn, the *i*Aβ accumulation, the T2 crossings, and the progression of AD all resume at the pre-treatment rate. Thus, the transient administration of the targeted *i*Aβ degradation therapy via the activation of BACE1 and/or BACE2 in symptomatic unconventional AD would provide only a short reprieve of slow progression of the disease for no longer than the duration of the treatment.

## 33. Composite Therapy for the Prevention and Treatment of Unconventional AD: (1) Combination of Transient ISR Suppression and Overlapping Long-Term Targeted *i*Aβ Degradation via the Activation of BACE1 and/or BACE2

The present and two following sections consider composite therapeutic strategies for the prevention and treatment of unconventional Alzheimer’s disease. The rationale for such strategies is conceptually the same as formulated in Section 25 above for conventional AD. Briefly, the rate of the targeted degradation of *i*Aβ via the activation of BACE1 and/or BACE2 apparently cannot match the rate of the influx of *i*Aβ produced in the AβPP-independent pathway. The apparent solution is to disable the AβPP-independent *i*Aβ generation pathway by the transient administration of inhibitors of the neuronal integrated stress response and thus to stop the influx of its *i*Aβ product for the duration of its depletion. In such circumstances, it is anticipated that the concurrent activation of BACE1 and/or BACE2 would be sufficient to rapidly deplete *i*Aβ to the low baseline levels and either to prevent/delay the commencement of AD or to stop the progression of the disease.

**Figure 23:**
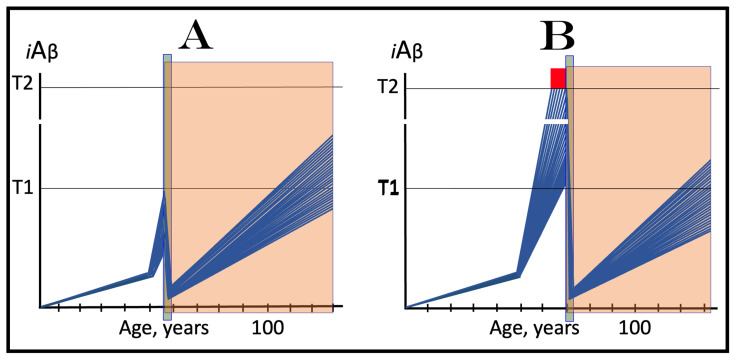
**Effects of the transient suppression of the ISR and the overlapping long-term targeted *i*Aβ degradation via the activation of BACE1 and/or BACE2 in the prevention and treatment of unconventional AD***. i*Aβ: intraneuronal Aβ. ***T*1** *threshold*: levels of *i*Aβ that trigger the activation of the eIF2α kinases PKR and/or HRI, elicitation of the neuronal ISR, and initiation of the AβPP-independent production of *i*Aβ. ***T*2** *threshold*: levels of *i*Aβ, produced mainly in the AβPP-independent pathway, which trigger neuronal death. *Blue lines*: levels of *i*Aβ in individual neurons of a person. *Red Box*: apoptotic zone; the fraction of neurons that have committed apoptosis (or necroptosis) or are dead. *Green Boxes*: the duration of the presence of ISR inhibitors. *Orange Boxes*: the duration of the presence of activators of BACE1 and/or BACE2. *Panel* (**A**): Effect of the transient suppression of the neuronal ISR and the overlapping long-term activation of BACE1 and/or BACE2 in the prevention of unconventional AD. By the commencement of the composite therapy, the neuronal IRS has been elicited by an unconventional stressor and the AβPP-independent *i*Aβ generation pathway has been unconventionally activated. Its *i*Aβ product has rapidly accumulated but has not yet reached the T1 threshold. The administration of an inhibitor of the IRS disables the AβPP-independent *i*Aβ generation pathway and stops the influx of its *i*Aβ product. The concurrent dispensation of the activator(s) of BACE1 and/or BACE2 results in the targeted degradation of *i*Aβ and its rapid depletion. The ISR inhibitor is withdrawn when *i*Aβ is depleted to a low baseline level. At this point, the neuronal ISR is re-elicited and the AβPP-independent *i*Aβ generation pathway is unconventionally re-activated due to the continuous presence of unconventional stressors, and the accumulation of *i*Aβ resumes de novo. However, because it commences at the low baseline and proceeds at a reduced rate (due to the continuous presence of BACE activators), the T1 threshold may not be reached within the remaining lifetime of the individual, and even if the T1 threshold is crossed, *i*Aβ levels may not reach the AD pathology-causing range for the duration of the treatment with the BACE-activating drug. *Panel* (**B**): The composite therapy is implemented when AD symptoms have already manifested. By this time, the neuronal ISR has been elicited by an unconventional stressor and the AβPP-independent *i*Aβ generation pathway has been unconventionally activated. Its *i*Aβ product has rapidly accumulated and crossed the T1 threshold. The AβPP-independent *i*Aβ generation pathway was rendered self-sustainable and AD commenced. The rapid accumulation of *i*Aβ continued; it reached AD pathology-causing levels and crossed the T2 threshold in a fraction of the neurons. Following the administration of the ISR inhibitor, the operation of the AβPP-independent *i*Aβ generation pathway and the influx of its *i*Aβ product cease. The concurrently administered activators of BACE1 and/or BACE2 mediate the targeted degradation of *i*Aβ and cause its rapid depletion. When the ISR-suppressing drug is withdrawn, the AβPP-independent *i*Aβ generation pathway resumes its operation and the accumulation of *i*Aβ re-commences from a low baseline and proceeds at a reduced rate (because of the continuous presence of the BACE-activating drug). *i*Aβ may not reach the T1 threshold and AD may not recur for the remaining lifespan of the treated individual. Even if the T1 threshold were crossed, the levels of *i*Aβ may not reach the AD pathology-causing range.

The effects of such composite therapy consisting of the transient suppression of the neuronal ISR and the long-term administration of a drug mediating the targeted degradation of *i*Aβ via the activation of BACE1 and/or BACE2 are illustrated in Figure 23. In panel A of Figure 23, the neuronal integrated stress response has been elicited by an unconventional stressor and, consequently, the AβPP-independent *i*Aβ generation pathway has been unconventionally activated at the low levels of AβPP-derived *i*Aβ. Due to its production independently of AβPP, *i*Aβ has rapidly accumulated but has not yet reached the T1 threshold. The administration of an inhibitor of the integrated stress response (green box) disables the AβPP-independent *i*Aβ generation pathway and stops the influx of its *i*Aβ product. The concurrent dispensation of the activator(s) of BACE1 and/or BACE2 results in the targeted degradation of *i*Aβ and its rapid depletion. The ISR inhibitor is withdrawn when *i*Aβ is depleted to a low baseline level. At this point, the neuronal ISR is re-elicited and the AβPP-independent *i*Aβ generation pathway is unconventionally re-activated due to the continuous presence of unconventional stressors, and the accumulation of *i*Aβ resumes de novo. However, because it commences at the low baseline and proceeds at a reduced rate (due to the continuous presence of BACE activators), the T1 threshold may not be reached within the remaining lifetime of the individual, and even if the T1 threshold is crossed, *i*Aβ levels may not reach the AD pathology-causing range for the duration of the treatment with the BACE-activating drug.

In panel B of Figure 23, the composite therapy is implemented when AD symptoms have already manifested. By this time, the neuronal ISR has been elicited by an unconventional stressor and the AβPP-independent *i*Aβ generation pathway has been unconventionally activated at the low levels of AβPP-derived *i*Aβ. Consequently, *i*Aβ rapidly accumulated and crossed the T1 threshold. The AβPP-independent *i*Aβ generation pathway was rendered self-sustainable and AD commenced. The rapid accumulation of *i*Aβ continued and it reached AD pathology-causing levels and crossed the T2 threshold in a fraction of the neurons. Following the administration of the ISR inhibitor, the operation of the AβPP-independent *i*Aβ generation pathway and, consequently, the influx of its *i*Aβ product cease. The concurrently administered activators of BACE1 and/or BACE2 mediate the targeted degradation of *i*Aβ and cause its rapid depletion. When the ISR-suppressing drug is withdrawn, the neuronal ISR is re-elicited by unconventional stressors, the AβPP-independent *i*Aβ generation pathway resumes its operation, and the accumulation of *i*Aβ re-commences from a low baseline and proceeds at a reduced rate (because of the continuous presence of the BACE-activating drug). *i*Aβ may not reach the T1 threshold and AD may not recur for the remaining lifespan of the treated individual. Even if the T1 threshold were crossed, the levels of *i*Aβ may not reach the AD pathology-causing range.

Importantly, in the composite therapeutic strategies, both preventive and curative, considered in the present section and those discussed in the following two sections, the duration of the transient ISR suppression should be such as to maximize the depletion of the *i*Aβ by BACE activators (which occurs only when the ISR is inhibited and, consequently, the AβPP-independent *i*Aβ production pathway is disabled; otherwise, the accumulation of *i*Aβ continues albeit at a reduced rate). This would maximize, upon the withdrawal of the ISR inhibitor, the time period required for *i*Aβ to reach the T1 threshold or AD pathology-causing levels, which in turn would maximize the benefits of the treatment.

## 34. Composite Therapy for the Prevention and Treatment of Unconventional AD: (2) Combination of Transient ISR Suppression and the Concurrent Transient Targeted Degradation of *i*Aβ via the Activation of BACE1 and/or BACE2

As discussed in the preceding section, a composite therapy consisting of the transient suppression of the integrated stress response and long-term activation of BACE1 and/or BACE2 would deliver a substantial beneficial value. However, the long-term administration of any drug could be potentially problematic. Thus, for example, long-term utilization of BACE1 inhibitors in an attempted AD therapy resulted in massive and severe adverse effects [13,14]. This is probably not the case with the BASE activators and almost certainly not the case with the activators of BACE1. Indeed, in the carriers of the Icelandic mutation, the efficiency of the β’-site cleavage by BACE1 is significantly enhanced (precisely the effect that we would like to achieve with BACE-activating drugs). This causes no adverse effects; on the contrary, the effects are highly beneficial, namely the protection from both AD and AACD [30,31]. Nevertheless, if the need arises, there is an alternative to the long-term administration of BACE activators. As described in the present and the following sections, BACE-activating drugs can be also utilized transiently in the prevention and treatment of unconventional AD.

**Figure 24:**
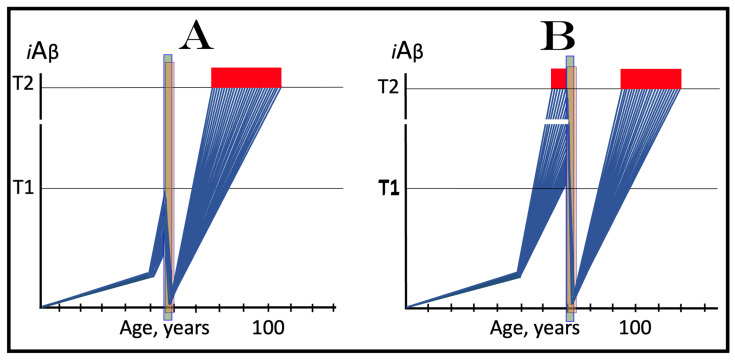
**Effects of the transient suppression of the ISR and the concurrent transient activation of BACE1 and/or BACE2 in the prevention and treatment of unconventional AD***. i*Aβ: intraneuronal Aβ. ***T*1** *threshold*: levels of *i*Aβ that trigger the activation of the eIF2α kinases PKR and/or HRI, elicitation of the neuronal ISR and initiation of the AβPP-independent production of *i*Aβ. ***T*2** *threshold*: levels of *i*Aβ, produced mainly in the AβPP-independent pathway, which trigger neuronal death. *Blue lines*: levels of *i*Aβ in individual neurons of a person. *Red Box*: apoptotic zone; the fraction of neurons that have committed apoptosis (or necroptosis) or are dead. *Green Boxes*: the duration of the presence of ISR inhibitors. *Orange Boxes*: the duration of the presence of activators of BACE1 and/or BACE2. *Panel* (**A**): Effect of the transient suppression of the neuronal ISR and the concurrent transient activation of BACE1 and/or BACE2 in the prevention of unconventional AD. At the commencement of the composite therapy, the neuronal IRS has been elicited by an unconventional stressor and the AβPP-independent *i*Aβ generation pathway has been unconventionally activated. Its *i*Aβ product has rapidly accumulated but has not yet reached the T1 threshold. The administration of an inhibitor of the IRS disables the AβPP-independent *i*Aβ generation pathway and stops the influx of its *i*Aβ product. The concurrent targeted degradation of *i*Aβ via the activation of BACE1 and/or BACE2 substantially depletes the levels of *i*Aβ. When both the ISR inhibitor and BACE activators are withdrawn, the accumulation of *i*Aβ resumes de novo from a low baseline. Because unconventional stressors are still present, the ISR is re-elicited and the AβPP-independent *i*Aβ generation pathway is unconventionally re-activated. *i*Aβ accumulates rapidly and unimpeded; when it crosses the T1 threshold, the AβPP-independent *i*Aβ production pathway becomes self-sustainable and AD commences and progresses. Thus, the concurrent transient administration of both ISR inhibitors and BACE activators would delay the occurrence of AD but the reprieve would be relatively short. *Panel* (**B**): Effect of the transient composite therapy in symptomatic unconventional AD. By the commencement of the composite therapy, the neuronal ISR has been elicited by unconventional stressors and the AβPP-independent *i*Aβ generation pathway has been unconventionally activated. Its *i*Aβ product has rapidly accumulated, crossed the T1 threshold, and reached AD pathology-causing levels and crossed the T2 threshold in a fraction of the neurons. Following the administration of the ISR inhibitor, the operation of the AβPP-independent *i*Aβ generation pathway and the influx of its *i*Aβ product cease. The concurrently administered activators of BACE1 and/or BACE2 mediate the targeted degradation of *i*Aβ and cause its rapid depletion. When both drugs are withdrawn, the neuronal ISR is re-elicited, the AβPP-independent *i*Aβ generation pathway re-activated, and the accumulation of *i*Aβ re-commences from a low baseline and proceeds unimpeded. It crosses the T1 threshold, reaches AD pathology-causing levels, and the progression of AD resumes. The composite therapy would stop the progression of AD only transiently. The reprieve would be short-lived; *i*Aβ levels would be relatively rapidly restored and the disease would recur. However, in both prevention and treatment applications, the duration of the reprieve would still be measured, probably in years.

The effects of a composite therapy for the prevention and treatment of unconventional AD consisting of the transient suppression of the ISR and the concurrent transient activation of the BACE1 and/or BACE2 are illustrated in Figure 24. In panel A of Figure 24, the neuronal integrated stress response has been elicited by an unconventional stressor at the low levels of AβPP-derived *i*Aβ. Consequently, the AβPP-independent *i*Aβ generation pathway has been unconventionally activated; *i*Aβ produced in this pathway has been rapidly accumulating but has not yet crossed the T1 threshold. The administration of the ISR-suppressing drug at this point disables the AβPP-independent *i*Aβ generation pathway and stops the influx of its *i*Aβ product. The concurrent implementation of the targeted degradation of *i*Aβ via the activation of BACE1 and/or BACE2 substantially depletes the levels of *i*Aβ. When both the ISR inhibitor and BACE activators are withdrawn, the accumulation of *i*Aβ resumes de novo from a low baseline. Because unconventional stressors are still present, the neuronal ISR is re-elicited and the AβPP-independent *i*Aβ generation pathway is unconventionally re-activated. As the result, *i*Aβ accumulates rapidly and unimpeded. When it crosses the T1 threshold, the AβPP-independent *i*Aβ production pathway becomes self-sustainable and AD commences. When it reaches AD pathology-causing levels, AD symptoms manifest. When it crosses the T2 threshold in a sufficient fraction of the neurons, the disease enters the end stage. Thus, the concurrent transient administration of both ISR inhibitors and BACE activators would delay the crossing of the T1 threshold and the occurrence of AD but, due to the high rate of the de novo accumulation of *i*Aβ, the reprieve would be relatively short but still measured probably in years.

In panel B of Figure 24, the drugs are administered when AD symptoms have already manifested. By this time, the neuronal ISR has been elicited by an unconventional stressor(s) and the AβPP-independent *i*Aβ production pathway has been unconventionally activated at the low levels of AβPP-derived *i*Aβ. As a result, *i*Aβ has rapidly accumulated and crossed the T1 threshold. The AβPP-independent *i*Aβ generation pathway has been rendered self-sustainable and AD has commenced. *iAβ* has continued its rapid accumulation. By the time of the drugs’ administration, it reached AD pathology-causing levels and crossed the T2 threshold in a fraction of the neurons. The dispensation of the ISR-suppressing drug at this stage abrogates the supply of the essential components of the AβPP-independent *i*Aβ generation pathway and thus disables it. The influx of *i*Aβ produced in this pathway ceases. This enables the efficient depletion of *i*Aβ by the concurrently administered BACE1- and/or BACE2-activating drug. When both the ISR inhibitors and the BACE activators are removed, the accumulation of *i*Aβ resumes de novo from a low baseline. The withdrawal of the drugs causes the re-elicitation of the neuronal ISR (due to the continuous presence of unconventional stressors) and the unconventional re-activation of the AβPP-independent *i*Aβ production pathway. *i*Aβ rapidly accumulates (due to the absence of BACE activators) and crosses the T1 threshold. The AβPP-independent *i*Aβ production pathway becomes self-sustainable and AD commences. *i*Aβ reaches AD pathology-causing levels and when it crosses the T2 threshold in a sufficient neuronal fraction, the disease enters its end stage. Thus, this composite transient therapy would transiently stop the progression of AD. This reprieve, however, would be short-lived (yet still measured probably in years); *i*Aβ levels would be restored and the disease would recur.

## 35. Composite Therapy for the Prevention and Treatment of Unconventional AD: (3) the Recurrent Simultaneous Transient Administration of Inhibitors of the Neuronal ISR and Activators of BACE1 and/or BACE2

As elaborated in the preceding section, the relief provided by the composite therapy for the prevention and treatment of unconventional AD, whereupon both ISR inhibitors and BACE activators are administered concurrently and transiently, is only of a relatively short duration. However, the effects of such composite therapy, in both the preventive and curative applications, can be amplified. As seen in the preceding figure, in both types of application of this composite therapy, following the withdrawal of the drugs the de novo accumulation of *i*Aβ commences from a low baseline. Therefore, despite the operation of the AβPP-independent *i*Aβ generation pathway, the disease-free time it takes for *i*Aβ to accumulate to the T1 threshold (in the preventive application of the therapy) or to AD pathology-causing levels (in the curative application of the therapy) is not insignificant and is measured probably in years. With the second, appropriately timed, transient administration of this composite therapy the disease-free duration can be doubled, and it can be repeated recurrently many times, thus keeping the treated individuals disease-free for the remaining portion of their lifetime.

**Figure 25:**
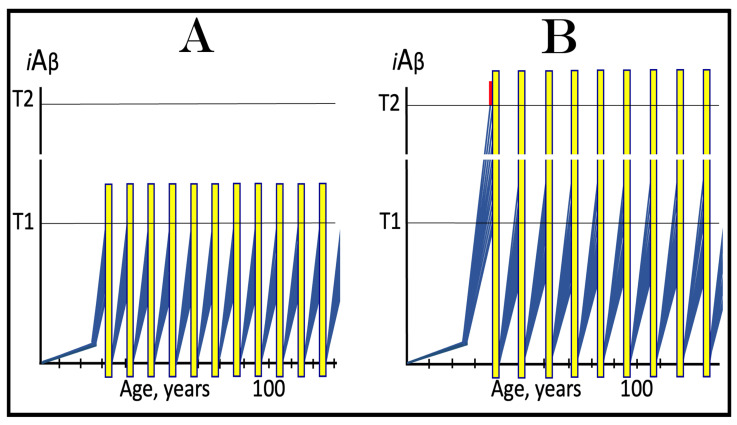
**Effects of the recurrent simultaneous transient administration of inhibitors of the neuronal ISR and activators of BACE1 and/or BACE2 in the prevention and treatment of unconventional AD***. i*Aβ: intraneuronal Aβ. ***T*1** *threshold*: levels of *i*Aβ that trigger the activation of the eIF2α kinases PKR and/or HRI, elicitation of the neuronal ISR, and initiation of the AβPP-independent production of *i*Aβ. ***T*2** *threshold*: levels of *i*Aβ, produced mainly in the AβPP-independent pathway, which trigger neuronal death. *Blue lines*: levels of *i*Aβ in individual neurons of a person. *Red Box*: apoptotic zone; the fraction of neurons that have committed apoptosis (or necroptosis) or are dead. *Yellow Boxes*: concurrent transient administration of inhibitor(s) of the ISR and activator(s) of BACE1 and/or BACE2 (shown as a single box due to space limitation). *Panel* (**A**): Effect of the recurrent transient implementation of the composite therapy in prevention of unconventional AD. At the commencement of the initial round of the composite therapy, the neuronal IRS has been elicited by an unconventional stressor and the AβPP-independent *i*Aβ generation pathway has been unconventionally activated. Its *i*Aβ product has rapidly accumulated but has not yet reached the T1 threshold. The administration of an inhibitor of the IRS disables the AβPP-independent *i*Aβ generation pathway and stops the influx of its *i*Aβ product. The concurrent activation of BACE1 and/or BACE2 substantially depletes the levels of *i*Aβ. When both ISR inhibitors and BACE activators are withdrawn, the neuronal ISR is re-elicited by unconventional stressors and the AβPP-independent *i*Aβ generation pathway is unconventionally re-activated; the accumulation of *i*Aβ resumes de novo from a low baseline but at a high rate and continues for certain duration before approaching the T1 threshold. At this stage, the composite therapy is implemented second time and then repeated recurrently as needed for the remaining lifetime. The T1 threshold is not crossed, and AD does not occur for the duration of the therapy. *Panel* (**B**): Effect of the recurrent transient implementation of the composite therapy in the treatment of unconventional AD. By the commencement of the initial round of the transient composite therapy, the neuronal ISR has been elicited by an unconventional stressor and the AβPP-independent *i*Aβ generation pathway has been unconventionally activated. Its *i*Aβ product has rapidly accumulated, crossed the T1 threshold, and reached AD pathology-causing levels and crossed the T2 threshold in a fraction of the neurons. The initial transient composite therapy results in a substantial depletion of *i*Aβ. Following the withdrawal of both ISR-suppressing and BACE-activating drugs, the neuronal ISR is re-elicited, the AβPP-independent *i*Aβ production pathway is unconventionally re-initiated, and the de novo accumulation of *i*Aβ resumes from a low baseline but at a high rate and proceeds unimpeded. At the appropriate time, defined by the appearance of suitable biomarkers, before *i*Aβ levels have reached the AD pathology-causing range, the second composite transient treatment is implemented and afterwards repeated recurrently, as needed, for the remaining lifetime of the patient. Following the initial composite treatment, no AD pathology-causing levels of *i*Aβ are reached and no symptomatic AD recurs for the duration of the therapy.

The effects of the recurrent administration of such composite transient therapy for the prevention and treatment of unconventional AD are illustrated in Figure 25. In panel A of Figure 25, at the time of the initial implementation of the composite therapy the neuronal ISR has been elicited by unconventional stressors at the low levels of AβPP-derived *i*Aβ. Consequently, the AβPP-independent *i*Aβ generation pathway has been unconventionally activated. As a result, *i*Aβ has rapidly accumulated but has not yet reached the T1 threshold. Following the administration of its inhibitor, the neuronal ISR is suppressed. Consequently, the supply of the essential components required for the operation of the AβPP-independent *i*Aβ generation pathway is discontinued; the pathway is disabled and the influx of its *i*Aβ product ceases. The concurrent administration of BACE1- and/or BACE2-activating drugs (due to space constrains in the Figure 25 the concurrent treatments with ISR inhibitors and BACE activators are shown as single yellow boxes) results in the effective depletion of *i*Aβ. When both ISR inhibitors and BACE activators are withdrawn, the neuronal ISR is re-elicited by unconventional stressors, and the AβPP-independent *i*Aβ generation pathway is unconventionally re-activated. The accumulation of *i*Aβ resumes de novo from a low baseline but at a high rate and continues unimpeded for certain duration (probably measured in years) before approaching the T1 threshold. At this stage, determined by the occurrence of suitable biomarkers or other indications, the composite therapy is implemented second time and then repeated recurrently as needed for the remaining portion of the lifetime. The T1 threshold is not crossed and AD does not occur for the duration of the therapy.

In panel B of Figure 25, the initial composite therapy is implemented when AD symptoms have already manifested. By this time, the neuronal integrated stress response has been elicited by the unconventional stressors and the AβPP-independent *i*Aβ production pathway has been unconventionally activated at the low sub-T1 levels of AβPP-derived *i*Aβ. As a result, *i*Aβ has rapidly accumulated and crossed the T1 threshold. The AβPP-independent *i*Aβ production pathway became self-sustainable and AD commenced. *i*Aβ has continued its accumulation, reached AD pathology-causing levels and crossed the T2 threshold in a fraction of the neurons. The initial transient composite therapy described above, implemented at this point, results in a substantial depletion of *i*Aβ. Following the withdrawal of both, ISR-suppressing and BACE-activating drugs, the neuronal ISR is re-elicited, the AβPP-independent *i*Aβ production pathway is unconventionally re-initiated, and the de novo accumulation of *i*Aβ resumes from a low baseline but at a high rate and proceeds unimpeded. At the appropriate time, defined by the appearance of suitable biomarkers, before *i*Aβ levels have reached the AD pathology-causing range, the second composite transient treatment is implemented and afterwards repeated recurrently, guided by appropriate biomarkers and other indications, for the duration of the patient’s lifetime. Following the initial composite treatment, no AD pathology-causing levels of *i*Aβ are reached and no symptomatic AD recurs for the duration of the therapy.

## 36. Final Remarks

### 36.1. Potential Therapeutic Outcomes in Unconventional AD Cases Where Unconventional Stressors Are Present Only Transiently

At this point, it should be reminded that in analyzing possible therapeutic options for unconventional AD, only the “worst case scenario” was considered, namely the continuous presence of unconventional stressors capable of eliciting the neuronal ISR (i.e., from the time of their appearance for the remaining lifetime; see Section 29 above). If the occurrence of such unconventional stressor were only transient, the outcomes of the composite therapies considered above for unconventional AD could be different. The decisive factor determining such outcomes is the timing of the exclusion of the unconventional neuronal ISR-eliciting stressors from the neuronal cells. If they were no longer present at the time of the withdrawal of the ISR-suppressing drug, the ISR would not be re-elicited, the AβPP-independent *i*Aβ production pathway would not be unconventionally re-activated, and the outcomes would be the same as described above in the analogous situations for conventional AD. If the unconventional stressors disappear only after *i*Aβ, newly accumulated following the withdrawal of the ISR inhibitor, crosses the T1 threshold, the outcomes would be the same as during their continuous presence (because at this stage the AβPP-independent *i*Aβ production pathway is rendered self-sustainable). Finally, if the unconventional stimuli are excluded following the transient ISR suppression treatment but prior to the T1 crossing by newly accumulated *i*Aβ, the crossing of the T1 threshold (and the commencement or recurrence of AD) would occur sooner than in the analogous conventional AD cases but later than during the continuous presence of the unconventional stressors (because the operation of the AβPP-independent *i*Aβ generation pathway would cease due to the absence of unconventional stressors and to sub-T1 levels of *i*Aβ, and the accumulation of *i*Aβ would be supported only by the slow influx of AβPP-derived *i*Aβ).

### 36.2. The Disease Will Persist as Long as the Levels of iAβ Remain Within the AD Pathology-Causing Range

Another “worst case scenario” considered in the present study is the efficiency (the rate) of the efflux of *i*Aβ by its targeted degradation via the activation of BACE1 and/or BACE2 versus the rate of the influx of *i*Aβ generated in the AβPP-independent pathway. For the purposes of the analysis, it was assumed that the latter exceeds the former. For the reasons elaborated above (Section 19, Section 20, Section 21, Section 22 and Section 23), this assumption is plausible, approaching a certainty if the AβPP-independent *i*Aβ production pathway is underpinned by the asymmetric RNA-dependent amplification of AβPP mRNA. As long as the rate of the influx of *i*Aβ exceeds that of its efflux, the progression of the disease would continue, albeit at a reduced pace. The reduction in the rate of the progression of AD would, in fact, be directly proportional to the efficiency of the removal of *i*Aβ. However, even if the efflux of *i*Aβ were more efficient than its influx, the disease would persist as long as the levels of *i*Aβ remain within the AD pathology-causing range. The key to the successful treatment of AD, therefore, is to deplete *i*Aβ as rapidly as possible and as extensively as possible (with the parameters defined and the consequences described in the preceding sections) and to maintain its levels below the AD pathology-causing range. The cessation of the influx of *i*Aβ generated in the AβPP-independent pathway in concert with its targeted degradation is, apparently, the most effective approach to attain this goal. For the prevention of conventional AD prior to the initiation of the AβPP-independent *i*Aβ production pathway, the targeted degradation of *i*Aβ via the activation of BACE1 and/or BACE2 would be sufficient on its own.

### 36.3. The Importance of Determining the Conventional versus Unconventional Nature of AD Cases

An additional important consideration is determining the conventional or unconventional nature of every case of symptomatic AD for the purposes of its treatment. Since in both cases the AβPP-independent *i*Aβ generation pathway is operational, making such a determination is not simple. Moreover, as mentioned above (Section 27) and discussed elsewhere [8], conventional AD can potentially self-transform into unconventional AD, and it could be challenging to distinguish between the two prior to the treatment. However, it is not exigent to differentiate between the types of AD in order to implement the composite therapy (or the initial round of the recurring composite therapy), which would be the same in both cases. In fact, the nature of the disease would be made apparent by its response to the treatment. If, following the treatment, symptoms (or biomarkers and other indications) do not recur, the disease was, in all likelihood, conventional (or the neuronal occurrence of unconventional stressors was transient). If symptoms do recur relatively soon (i.e., within a few years), the disease is likely unconventional and the recurrent round(s) of treatment are indicated.

On the other hand, the approaches for prevention of conventional and unconventional AD are qualitatively as distinct as are the surrounding circumstances. Prevention of conventional AD would be carried out in the context of the inoperative AβPP-independent *i*Aβ generation pathway, and a single transient depletion of *i*Aβ via the activation of BACE1 and/or BACE2 implemented in mid-life is apparently sufficient to prevent conventional AD and AACD for the remaining lifetime. In contrast, in prevention of unconventional AD the AβPP-independent *i*Aβ production pathway has already been unconventionally activated, and to be effective BACE activation-mediated degradation of *i*Aβ must be combined with the suppression of the neuronal ISR (or with the inhibition of the AβPP-independent *i*Aβ production pathway by other means). Therefore, in implementing the preventive AD therapy, the determination of the operational versus inoperative status of the AβPP-independent *i*Aβ generation pathway could be of considerable importance. The methods for such determination are described in Section 26 above.

### 36.4. The Potential Double Duty of ISR Inhibitors in AD Therapy: (1) Disabling the AβPP-Independent iAβ Generation Pathway and (2) Enabling the Production of BACE1 and BACE2

Some composite AD therapy scenarios, described above in Section 25 (for conventional AD; see Figure 13, panel A) and Section 33 (for unconventional AD; see Figure 23), entail the long-term activation of BACE1 and/or BACE2 following the withdrawal of inhibitors of the integrated stress response, i.e., under ISR conditions. In these scenarios, it is assumed that BACE1 and BACE2 continue to be produced under ISR conditions, when translation of the bulk of cellular proteins is suppressed as a result of the phosphorylation of eIF2α. This may not be the case; the continuous production and presence of BACE1 and BACE2 in human neurons under ISR conditions remain to be ascertained (this can be achieved using the human neuronal cell-based AD model described in [7]). On the other hand, BACE1 and BACE2 are certainly produced in neurons under regular (i.e., non-ISR) conditions. Therefore, even if the continuous production and, consequently, the cellular presence of BACE1 and BACE2 were affected by the ISR (both BACE1 and BACE2 are redundant in the production of *i*Aβ, independently of AβPP, and their absence would impede neither the generation of *i*Aβ in the AβPP-independent pathway nor the progression of AD), the transient administration of their activators in the presence of ISR inhibitors (i.e., under non-ISR conditions), as described in Section 25 for conventional AD (see Figure 13, panel B) and Section 34 and Section 35 for unconventional AD (see Figure 24 and Figure 25), would remain fully applicable. In other words, BACE1 and BACE2 may not be produced in the neurons under ISR conditions. In such a case, their utilization as *i*Aβ degradation agents would be feasible both for the prevention of conventional AD (when no neuronal ISR has yet been elicited; see Section 18 and Figure 9, panel A) and in the presence of ISR inhibitors (in circumstances when the neuronal ISR has already been elicited; the relevant sections and figures are listed in the section above). In this scenario, the inhibition of the neuronal ISR has a double duty. One, it disables the AβPP-independent *i*Aβ generation pathway and, consequently, stops the influx of *i*Aβ that is produced in this pathway. Two, by enabling the production of BACE1 and BACE2, it makes their utilization as *i*Aβ degradation agents possible. Therefore, as an example, even if the rate of degradation of *i*Aβ by activated BACE1 and/or BACE2 were to match or exceed that of its generation in the AβPP-independent pathway (as illustrated in Figure 9, panel B), the therapeutic utilization of both activities may still require the suppression of the neuronal ISR in order to enable their production.

## 37. Conclusions: Alzheimer’s Disease Is Preventable and Treatable

The present study considers two therapeutic strategies for the prevention and treatment of Alzheimer’s disease. One strategy is the suppression of the neuronal integrated stress response (ISR). Another strategy is the depletion of intraneuronal Aβ, *i*Aβ. Each strategy is guided by a theory of AD designated the Amyloid Cascade Hypothesis 2.0 (ACH2.0), and each has a solid rationale. In the ACH2.0, the disease is initiated by the elicitation of the neuronal ISR. In conventional AD, this is triggered by the physiological accumulation of AβPP-derived *i*Aβ over the critical level (the T1 threshold). When this occurs, PKR and/or HRI kinases are activated, eIF2α is phosphorylated at its Ser51 residue and the neuronal ISR ensues. In unconventional AD, the elicitation of the neuronal ISR is triggered by stressors distinct from AβPP-derived *i*Aβ and occurs below the T1 threshold. Under the radical transcriptional/translational ISR-induced reprogramming, the essential components of the AβPP-independent *i*Aβ generation pathway are produced and the pathway becomes operational. The entire Aβ outcome of this pathway is retained within the neurons as *i*Aβ. It rapidly accumulates and reaches AD pathology-causing levels. Subsequently, it crosses the apoptosis- or necroptosis-triggering T2 threshold and neuronal death follows. Thus, *i*Aβ produced independently of AβPP drives AD pathology. Since its levels are maintained over the T1 threshold, it also propagates the ISR conditions and thus perpetuates its own production in the AβPP-independent pathway.

In light of the above, the rationale for the suppression of the integrated stress response in AD is apparent: the long-term inhibition of the ISR would disable the AβPP-independent *i*Aβ generation pathway and potentially stop progression of the disease. This, however, is not the case. First, even if the long-term suppression of the ISR were feasibly implemented, the effect would be only the impediment, slowing down the progression of AD, not its cessation. Second, the long-term suppression of the ISR is unconceivable and unfeasible. This is because the ISR is a fundamental cell survival tool and its suppression would reduce cell viability and could be highly deleterious. This point is exemplified by eIF2α Ser51Ala KI mice. In these mice, alanine (substituting serine) in position 51 of eIF2α cannot be phosphorylated and, consequently, the ISR cannot be elicited. These mice survive no longer than eighteen hours after their birth. On the other hand, as was shown in experimental mouse models, the ISR inhibitors can be employed transiently without noticeable adverse effects. However, as discussed above, the transient suppression of the ISR, implemented as an AD therapy, would, on its own, provide only a minor reprieve and only for the duration of the treatment.

The rationale for the depletion of *i*Aβ as therapeutic strategy for AD is no less apparent: reduce levels of *i*Aβ below those causing AD pathology and the progression of the disease would stop; deplete *i*Aβ to levels below the T1 threshold and the AβPP-independent *i*Aβ generation pathway would be rendered unsustainable and inoperative (unless supported unconventionally). Deplete AβPP-derived *i*Aβ before it reaches the T1 threshold in the healthy individual and the disease would be prevented (or substantially delayed). Physiologically occurring Icelandic and Flemish mutations of Aβ provide both a proof of concept for this notion and an indication for how to implement the targeted degradation of *i*Aβ. Both mutations modify the efficiencies of the physiological intra-*i*Aβ cleaving activities of BACE1 and BACE2. Thus, the Icelandic Aβ mutation increases by only 30% the efficiency of the intra-*i*Aβ cleavage at the β’-site by BACE1 but this is sufficient to protect from both AD and AACD (aging-associated cognitive decline) because it reduces the rate of accumulation of AβPP-derived *i*Aβ and prevents it from crossing the T1 threshold. The Flemish Aβ mutation, on the other hand, reduces the physiologically occurring intra-*i*Aβ cleavage by BACE2. This increases the rate of accumulation of AβPP-derived *i*Aβ, accelerates the T1 crossing and thus causes familial early-onset AD. It is apparent, therefore, that the activation of the intra-*i*Aβ cleaving capabilities of BACE1 and/or BACE2 could be of a substantial therapeutic value.

The effects of the Icelandic and Flemish Aβ mutations are manifested prior to the T1 crossing, when the AβPP-independent *i*Aβ production pathway is inoperative. However, in AD, this pathway is operational and, as discussed above, the rate of the influx of its *i*Aβ product is apparently orders of magnitude higher than the rate of the influx of *i*Aβ generated in the AβPP proteolytic pathway. It is, therefore, highly unlikely that, with the AβPP-independent *i*Aβ generation pathway operational, the rate of the targeted degradation of *i*Aβ (i.e., of its efflux) by activated BACE1 and/or BACE2 or by any other suitable agent can exceed that of its influx, the precondition of its depletion. The apparent solution of this problem is to combine, in a carefully coordinated manner discussed above, the transient suppression of the neuronal integrated stress response with the concurrent activation of BACE1 and/or BACE2 (or with the concurrent utilization of any other suitable *i*Aβ-degrading agent) in one composite therapy for AD. The purpose of the ISR suppression in such combination is to disable the AβPP-independent *i*Aβ generation pathway and to abolish altogether the influx of its *i*Aβ product (and, potentially, to enable the production of BACE1 and BACE2; see the preceding section). This would enable the efficient depletion of *i*Aβ by concurrently activated BACE1 and/or BACE2.

The present study considers the separate and concurrently combined implementations of the suppression of the neuronal integrated stress response and of the targeted degradation of *i*Aβ via the activation of BACE1 and/or BACE2 (or by other suitable agents) in the contexts of both conventional and unconventional Alzheimer’s disease. The principal conclusions of the analysis presented above are the following:

1. One-time-only depletion of *i*Aβ implemented prior to the T1 crossing by AβPP-derived *i*Aβ (at this stage, neither is the ISR elicited nor the AβPP-independent *i*Aβ generation pathway activated) via the activation of BACE1 and/or BACE2 could be sufficient to prevent the occurrence of conventional AD and of AACD for the remaining lifetime of the treated individual;
2. In conventional symptomatic AD, the transient suppression of the neuronal ISR combined concurrently with the activation of BACE1 and/or BACE2 would stop the progression of the disease and prevent its recurrence within the remaining lifespan of the treated patient. The transient administration of the BACE-activating drug in such context may be sufficient for this effect; the long-term activation of BACE1 and/or BACE2 (or the employment of other suitable *i*Aβ-degrading agents) would certainly suffice;
3. The transient suppression of the neuronal ISR combined concurrently with the long-term activation of BACE1 and/or BACE2 could be sufficient to prevent the occurrence of unconventional AD for the remaining lifetime of the treated person. Alternatively, both components of the composite therapy, the suppression of the neuronal ISR, and the simultaneous activation of BACE1 and/or BACE2 can be implemented transiently but recurrently, with the same effect;
4. In unconventional symptomatic AD, the transient suppression of the neuronal integrated stress response implemented concurrently with the long-term administration of the BACE1- and/or BACE2-activating drugs could be sufficient to stop the progression of the disease and to prevent its recurrence within the remaining lifetime of the treated patient. Alternatively, both treatments comprising the composite therapy can be implemented transiently but recurrently, with the same outcome.
